# Quantitative Characterization of Gene Regulatory Circuits Associated With Fungal Secondary Metabolism to Discover Novel Natural Products

**DOI:** 10.1002/advs.202407195

**Published:** 2024-10-28

**Authors:** Xinran Xu, Yanhong Sun, Anxin Zhang, Sijia Li, Shu Zhang, Sijing Chen, Chunbo Lou, Lei Cai, Yihua Chen, Chunxiong Luo, Wen‐Bing Yin

**Affiliations:** ^1^ State Key Laboratory of Mycology Institute of Microbiology Chinese Academy of Sciences Beijing 100101 P. R. China; ^2^ Medical School University of Chinese Academy of Sciences Beijing 100049 P. R. China; ^3^ Center for Quantitative Biology Academy for Advanced Interdisciplinary Studies Peking University Beijing 100871 P. R. China; ^4^ The State Key Laboratory for Artificial Microstructures and Mesoscopic Physics School of Physics Peking University Beijing 100871 P. R. China; ^5^ CAS Key Laboratory of Quantitative Engineering Biology Shenzhen Institute of Synthetic Biology Shenzhen Institutes of Advanced Technology Chinese Academy of Sciences Shenzhen 518055 P. R. China; ^6^ State Key Laboratory of Microbial Resources Institute of Microbiology Chinese Academy of Sciences Beijing 100101 P. R. China; ^7^ Wenzhou Institute University of Chinese Academy of Sciences Wenzhou Zhejiang 325001 P. R. China

**Keywords:** filamentous fungi, gene regulatory circuits, microfluidics, quantification, secondary metabolism

## Abstract

Microbial genetic circuits are vital for regulating gene expression and synthesizing bioactive compounds. However, assessing their strength and timing, especially in multicellular fungi, remains challenging. Here, an advanced microfluidic platform is combined with a mathematical model enabling precise characterization of fungal gene regulatory circuits (GRCs) at the single‐cell level. Utilizing this platform, the expression intensity and timing of 30 transcription factor‐promoter combinations derived from two representative fungal GRCs, using the model fungus *Aspergillus nidulans* are determined. As a proof of concept, the selected GRC combination is utilized to successfully refactor the biosynthetic pathways of bioactive molecules, precisely control their production, and activate the expression of the silenced biosynthetic gene clusters (BGCs). This study provides insights into microbial gene regulation and highlights the potential of platform in fungal synthetic biology applications and the discovery of novel natural products.

## Graphical abstract

An advanced microfluidic platform is created to enable precise characterization of complex multicellular fungal gene regulatory circuits (GRCs) at the single‐cell level. Quantification of GRCs involved in secondary metabolite production provides new insights into gene regulation of fungal secondary metabolism and highlights the potential of synthetic biology and the discovery of novel natural products.

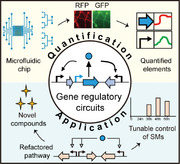

## 1 Introduction

Synthetic biology employs engineering principles to construct, reorganize, and program cellular behaviors, creating “cell factories” that harness microorganisms' robust and diverse biochemical reaction networks to produce various value‐added products.^[^ ^1^, ^2^, ^3^ ^]^ This includes the supply of 1,3‐propanediol (PDO),^[^ ^4^, ^5^, ^6^ ^]^ 1,4‐butanediol (BDO),^[^ ^7^, ^8^ ^]^ and alkane gases^[^ ^9^, ^10^ ^]^ in the energy and chemical industries; artemisinic acid,^[^ ^11^, ^12^ ^]^ tropane alkaloids,^[^ ^13^ ^]^ and cannabinoids^[^ ^14^ ^]^ in the pharmaceutical sector; and starch,^[^ ^15^ ^]^ glucose^[^ ^16^ ^]^ and vitamins^[^ ^17^ ^]^ in the food industry. Harnessing cell factories can serve as an effective strategy for a sustainable, low‐carbon economy, potentially minimizing the requirement for arable land, thereby reducing detrimental effects of animals and plants. Two of the most common cell factories are *Escherichia coli* and *Saccharomyces cerevisiae*, due to their short growth cycles, relatively well‐defined metabolic pathways and gene expression regulatory networks, and the existence of comprehensive databases. The advantages of these two cell factories provide accurate quantifications of standardized gene elements and modular gene regulatory circuits for the construction of engineered cell factories.^[^ ^18^, ^19^, ^20^, ^21^, ^22^, ^23^ ^]^ Despite these advantages, small‐cell factory loads and other challenges still existent.

Filamentous fungi have been employed for a long time in the fermentation of food products and have an exceptional capacity for secondary metabolite synthesis, making them a vital resource for antibiotics production. Filamentous fungi are well suited as cell factories because of their enhanced secretion ability and abundant substrate utilization, as well as their ability to directly express genes of eukaryotic origin.^[^ ^24^, ^25^, ^26^, ^27^ ^]^ However, the development of synthetic biology systems and precise control of filamentous fungi production lines is a relatively nascent field of research,^[^ ^28^ ^]^ and a number of challenges and issues still need to be addressed and resolved. Fungal gene regulatory networks are highly complex, and regulatory genes are interconnected, influencing each other in intricate ways.^[^ ^29^, ^30^ ^]^ Even the slightest change in gene expression can impact the entire system. Therefore, obtaining orthogonalized regulatory elements adapted to synthetic biology is difficult, accurately quantifying gene expression in specific cellular processes is likewise a major challenge.^[^ ^31^, ^32^ ^]^

**Figure 1:**
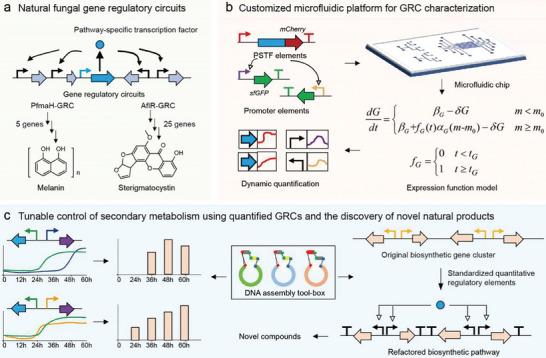
Acquisition, quantification, and application of regulatory elements of gene regulatory circuits. a) Natural fungal gene regulatory circuits (GRCs)‐associated with fungal secondary metabolism. Within the fungal biosynthetic gene clusters (BGCs), there is a category of gene clusters that are regulated by transcription factors within the cluster, resulting in the formation of orthogonal GRCs. b) Customized microfluidic platform for quantitative characterization of GRCs at the single‐cell levels. Pathway‐specific transcription factors (PSTFs) and promoters are the regulatory elements that play a crucial role in GRCs. It is essential to accurately characterize these elements to ensure proper regulation. To achieve this, quantitative methods that meet the criteria for long‐term dynamic monitoring at the single‐cell level are necessary. c) Tunable control of secondary metabolism using quantified GRCs and the discovery of novel natural products. By accurately characterizing the regulatory elements, it is possible for multiple biosynthetic genes fused to promoters to co‐express in the presence of a single PSTF, and to utilize them to precisely control the production of secondary metabolites, including their timing and intensity. Novel natural products can also be obtained by refactoring the secondary metabolite synthesis pathway.

Microorganisms, as drug producers, have evolved a variety of regulatory mechanisms to ensure the accurate production of secondary metabolites. These genes are typically grouped together in biosynthetic gene clusters (BGCs).^[^ ^33^ ^]^ In fungi, secondary metabolism is intricately regulated through a complex hierarchical regulatory network involving numerous regulators that implement pathway‐specific, broad‐domain, and epigenetic regulations.^[^ ^29^ ^]^ Transcription factors (TFs), located in or outside BGCs, mediate pathway‐specific regulation by binding to specific DNA sequences of cluster genes,^[^ ^34^ ^]^ forming gene regulatory circuits (GRCs). Tight regulation of BGC members by TFs ensures gene expression and production of certain secondary metabolites (**Figure** **1**a). Taking *A. fumigatus* as an example, ≈50% of BGCs contain at least one TF (the remaining BGCs lack transcription factors), making them suitable as a model for quantitative characterization.^[^ ^34^ ^]^ Once precisely quantified, these regulatory elements can be used as tools for the rational design of predictable programmable genetic circuits.^[^ ^35^ ^]^

Currently, the usage of quantitative techniques can be broadly categorized into two main groups. The first category combines fluorescence microscopy with flow cytometry or microfluidic techniques to track elements labeled by fluorescent or metabolite markers at the population or single‐cell level. However, for multicellular organisms, the collection of fluorescence signals is significantly affected by the multicellular network. Some studies^[^ ^24^, ^36^, ^37^, ^38^ ^]^ have attempted to break down multicellular cells into single cells by producing protoplasts, but this approach cannot obtain complete data on life processes occurring on a time scale. The second category is single‐cell sequencing‐based global analysis.^[^ ^39^, ^40^, ^41^, ^42^, ^43^, ^44^ ^]^ While live single‐cell sequencing systems have been developed to overcome the temporal limitations of single‐cell sequencing technologies,^[^ ^45^ ^]^ this approach is still highly non‐scalable and covers a limited range of cell types. It is not easy to collect live cell data for a long time without affecting cell growth. To overcome these limitations, microfluidic technology provides a well‐controlled cellular microenvironment to enable continuous growth of single cells and dynamic characterization of regulatory elements.^[^ ^46^, ^47^ ^]^ These tools have been used to investigate cellular inheritance,^[^ ^48^, ^49^ ^]^ and aging.^[^ ^50^, ^51^, ^52^ ^]^

Here, we designed customized microfluidic chips to facilitate the growth of multicellular structures of *Aspergillus nidulans* in the same focal plane, while minimizing disturbance to the normal growth of filamentous fungi. This allowed us to collect dynamic expression data of GRC combinations at the single‐cell level (Figure 1b). Using this approach, we characterized 30 sets of transcription factor‐promoter pairs from two selected GRCs, and quantitatively analyzed their expression patterns. Ultimately, the quantified GRCs were successfully used to control the yields of bioactive metabolites and achieve the production of novel natural product dendrobines by refactored biosynthetic pathways.

## 2 Result

### 2.1 Overview of a Quantified Method for Fungal GRCs Design

The precise quantification of regulatory processes is essential for understanding gene expression and regulation. The orthogonal GRC associated with fungal secondary metabolism provides a simple and effective model for quantification. To systematically evaluate GRCs in fungi, two selected GRCs were analyzed (Figure 1a): one regulates the synthesis of 1, 8‐dihydroxynaphthalene (DHN) melanin in *Pestalotiopsis fici*,^[^ ^53^, ^54^ ^]^ while the other regulates the synthesis of sterigmatocystin in *A. nidulans*.^[^ ^55^ ^]^ Melanin plays a significant role in fungal pathogenesis and defense,^[^ ^56^ ^]^ and the PKS genes that synthesize its precursor are widespread throughout the fungal kingdom.^[^ ^57^, ^58^ ^]^ Within the *P. fici* genome, the DHN melanin synthesis gene cluster is comprised of seven distinct genetic sequences. Five of these genes play a role in the biosynthesis of melanin, while two others serve as transcriptional regulators. One of these transcription factors, PfmaH, functions as a pathway‐specific regulator. Similarly, the GRC for sterigmatocystin in *A. nidulans* comprises 26 genes that are regulated by the transcription factor AflR.^[^ ^59^, ^60^, ^61^ ^]^ The homologous gene cluster containing AflR in many Aspergillus species is conserved for the production of mycotoxin. Due to its complex regulatory mechanisms and the more than 20 genes involved, each with its own unique dynamic pattern, this gene cluster has received great attention.^[^ ^30^ ^]^ These two GRCs together can be used as representations of the majority of GRC types. Accurate quantification of TF elements and promoter elements in GRCs is crucial. To achieve this, we employed microfluidic devices (Figure 1b), which allowed us to precisely measure regulatory combinations. The microfluidic chips are well‐customized for complex multicellular fungi. This resulted in standardized regulatory combinations that were able to reconfigure gene regulatory circuits.

In order to utilize GRCs for BGC refactoring, we optimized the MoClo DNA assembly method, which is based on the type IIS restriction enzyme.^[^ ^62^, ^63^, ^64^ ^]^ A DNA assembly toolbox was developed for filamentous fungi (Figures S1 and S2, Table S1, Supporting Information), which was used to fuse the promoters in GRCs with synthetic genes. The toolbox consists of all standard promoters in the PfmaH gene regulatory circuit (PfmaH‐GRC) and AflR gene regulatory circuit (AflR‐GRC), as well as the most efficient connectors published in scientific literature^[^ ^64^ ^]^ and receptor vectors customized for *A. nidulans*. By using this toolbox, standard promoters and receptor vectors were hierarchically constructed to create vectors that included refactor biosynthetic pathways (Figure 1c).

### 2.2 A Customized Microfluidic Quantitative Method for Complex Multicellular Fungi

**Figure 2:**
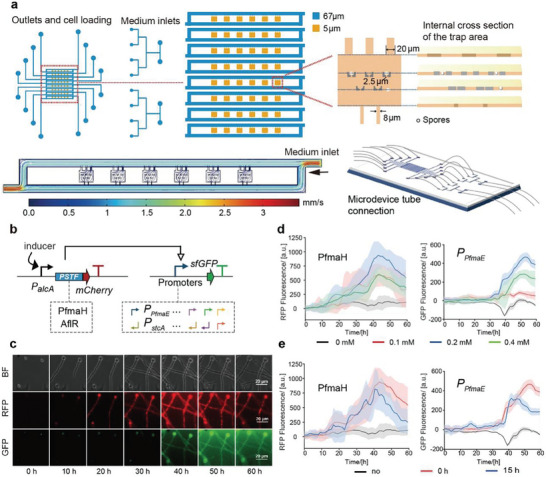
Microfluidic system for quantifying regulatory elements in multicellular fungi. a) A customized microfluidic chip designed for high‐throughput single‐spore observation of filamentous fungi. b) Design of genetic circuits for fluorescent monitoring of regulatory elements in GRCs. c) A diagram illustrating the fluorescence sequence and the collection of single‐cell fluorescence. Fluorescence monitoring was conducted for a total of 60 h. d) Optimization of inducer concentration for culturing filamentous fungi on microfluidic chips. The expression of PfmaH and P*_PfmaE_* at 0, 0.1, 0.2, and 0.4 mm cyclopentanone addition is depicted by the black, red, green, and blue lines, respectively. The shading indicates the range of population fluorescence, while the solid line represents the average of multiple‐cell fluorescence data. The sample sizes on the left graph are 22 (0 mm), 30 (0.1 mm), 37 (0.2 mm), and 30 (0.4 mm). The sample sizes on the right graph are 13 (0 mm), 10 (0.1 mm), 12 (0.2 mm), and 11 (0.4 mm). e) Optimization of the timing of inducer addition for culturing filamentous fungi on microfluidic chips. The expression of PfmaH and P*_PfmaE_* was observed under different conditions. The black, red, and blue lines represent the expression without the addition of cyclopentanone, with cyclopentanone added at 0 h, and at 15 h, respectively. The sample sizes on the left are 22 (no), 37 (0 h), and 12 (15 h). The sample sizes on the right diagram are 13 (no), 12 (0 h), and 10 (15 h). The concentration of cyclopentanone used in all cases was 0.2 mm.

To solve the problem of collecting fluorescence from multicellular layered hyphae and enabling dynamic single‐cell monitoring, we introduced microfluidic microarrays that were used to create a specialized microenvironment for spore germination, where *A. nidulans* conidia was innovatively used as fluorescence collection objects. Compared to protoplasts, which were used in previous quantitative approaches,^[^ ^24^, ^38^ ^]^ conidia were less brittle due to their thick cell walls. For long‐term single‐layer observation, we designed a multicellular microfluidic chip (**Figure** **2**a) with a height of 5 µm based on the diameter of *A. nidulans* conidia (≈4 to 5 µm, enlarged after germination). The entire chip measured 2.5 cm × 2 cm and contained two repeating structures. Each repeated structure was divided into four adjacent U‐shaped channels, enabling us to test eight strains of *A. nidulans* simultaneously. The chip size could be doubled to monitor 16 strains and apply 16 different conditions if necessary. Six chambers were arranged in a U‐shaped channel, with barriers positioned in the center of each chamber. The barrier prohibited the passage of conidia larger than 2.5 µm, but sometimes, the conidia also became trapped in other areas of the chambers (each chamber contained less than 5 cells at the beginning). This design allowed for the spreading and fixation of conidia and prevented the multilayer mycelium from disrupting data collection after spore movement and growth while ensuring spore growth space on the chip.

To develop a quantitative test method for TF‐promoter pairs, pathway‐specific transcription factors (PSTFs) were fused with red fluorescent protein mCherry, and promoters were combined with green fluorescent protein sfGFP. Fused DNA fragments were integrated into the chromosomes of *A. nidulans*, and the inducible promoter *P_alcA_* was used to control PSTF and initiate transcription of the genetic circuit. In such a genetic circuit, PSTF governed promoter intensity, with red fluorescence intensity defining the input value of the genetic circuit, and the output value defined by the fluorescence intensity of the sfGFP linked to the promoter (Figure 2b). A total of 5 dual‐fluorescence strains were created for PfmaH‐GRC, and 25 dual‐fluorescence strains were created for AflR‐GRC. One of the dual‐fluorescent strains (TYXR75) was used as an example to determine the optimal conditions for culturing *A. nidulans* conidia on microfluidic chips (Figures S3 and S4, Supporting Information). According to the findings, conidia exhibited normal germination and growth within a 60 h period on the chip (Figure 2c). However, the addition of conditioned media (CM), which refers to the MM medium after filtering out the mycelium from *A. nidulans* that were cultured for 96 h, was found to be essential for inducing and maintaining microfluidic culture (Figure S4b, Supporting Information).

To identify the best inducer concentration, several concentrations of cyclopentanone were added to the culture medium and monitored continuously for 60 h (Figure 2d). When the concentration of cyclopentanone was 0.2 mm, the peak reached its maximum value before beginning to drop. To determine the timing of inducer addition and to avoid the possible toxicity of the inducer on mycelium, cyclopentanone was injected after 0 and 15 h (Figure 2e). The results showed that the addition of 0.2 mm cyclopentanone at 0 h did not affect the normal growth of mycelium and induced better activation of *P_alcA_*. As a result, the optimized conditions for the expression were found to be 20% conditioned media with 0.2 mm cyclopentanone added at the start of the experiment (0 h) (Figure 2d,e; Figure S4b, Supporting Information).

### 2.3 Quantitative Characterization of Fungal GRCs Associated with Secondary Metabolism

**Figure 3:**
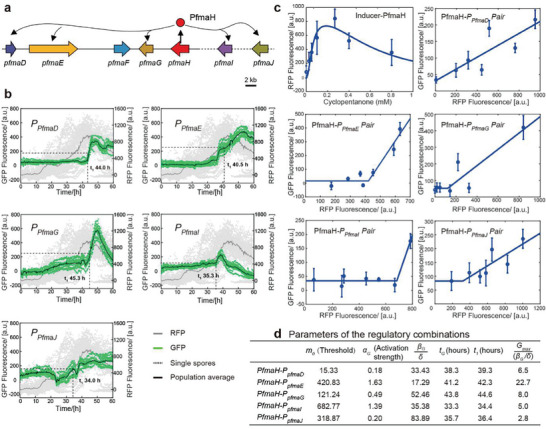
Dynamic analysis and quantification of regulatory elements in the PfmaH gene regulatory circuit. a) The PfmaH‐GRC is comprised of two transcription factors and five functional genes, with PfmaH serving as the specific regulator of this circuit. b) The expression of elements within the PfmaH transcription regulatory unit was monitored over a period of 60 h under optimal culture conditions. In the graph, each dashed line represents the fluorescence data of a single cell, while the solid line represents the average of multiple single‐cell fluorescence data over a period of 60 h. The gray section represents the red fluorescence data output, which indicates the expression level of PfmaH and is reflected on the right y‐axis, the sample sizes for all five graphs are 44. The green section represents the green fluorescence data output, which represents the expression level of the promoter fused with sfGFP. The sample sizes are 11 (*P_pfmaD_*), 12 (*P_pfmaE_*), 10 (*P_pfmaG_*), 11 (*P_pfmaI_*), and 11 (*P_pfmaJ_*) respectively. The t_1_ value indicates the time point when promoter expression reaches half the plateau height under optimal conditions (0.2 mm cyclopentanone). c) The effects of input data (expression of transcription factors) on output data (promoter intensity) are affected by different concentrations of inducers. d) The fitting parameters of regulatory element pairs of the PfmaH‐GRC were obtained by fitting the experimental data through the model, which can be used to characterize the dynamic behavior and interaction between the regulatory elements. Where *m*_0_ represents the threshold value required for activation of the promoters, α_*G*_ represents the activation strength of the promoters, β_*G*_/δ represents the basic value, *t_G_* represents the time needed for the promoters to start expression, and $\frac{G_{max}}{\beta_{G}/\delta }$ calculates the maximum expression relative to the basic value, *t*_1_ here is the fitted time when promoter expression reaches half the plateau height.

After determining the microfluidic conditions, we measured how the PfmaH precisely controlled the gene expression of its members. Conidia of five dual‐fluorescence strains of PfmaH‐GRC (**Figure** **3**a) were cultured in a microfluidic device for 60 h to evaluate the precision of gene expression control using microfluidics. Results showed that the red fluorescence intensity, which was used as a common input signal, had the same change pattern in all five strains. Specifically, the expression level of pfmaH decreased slightly at 15 h, peaked at 20 h, and then stabilized at a higher level (Figure 3b). The patterns of green fluorescence fluctuation varied among the strains (Figure 3b; Figure S5, Supporting Information), with promoter expression initiation occurring after *pfmaH* expression, and high promoter expression initiation time occurring between 34 and 45 h. P_*PfmaG*_ and P_*PfmaE*_ had high expression levels in the PfmaH‐GRC.

In order to gain a better understanding of how PfmaH dynamically regulates the expressions of its members, a simple mathematical model was constructed to illustrate this phenomenon. Our results showed that changes in cyclopentanone concentration resulted in variations in *pfmaH* expression. To obtain data for promoters exhibiting different expression modes, we designed multiple gradients (Figure 3c; Figures S6–S11, Supporting Information). The experimental results indicate that the expression of the transcription factor increases and then decreases with increasing cyclopentanone concentration. The decrease in transcription factor expression at higher cyclopentanone concentrations may be related to biological toxicity. To capture the transcription factor expression characteristics under cyclopentanone induction, a Hill‐type biophysical model^[^ ^65^ ^]^ was adopted which assumes the basal expression rate of transcription factor is zero:

(1) $$\frac{dm}{dt}=f_{m}\left(t\right)\frac{\alpha_{m}{\left(\frac{I}{K}\right)}^{2}}{\left(1+{\left(\frac{I}{K}\right)}^{2}\right)\left(1+{\left(\frac{I}{K_{1}}\right)}^{2}\right)}-\delta m$$

(2) $$f_{m}\left(t\right)=\left\{\begin{array}{cc} 0 & t<t_{m}\\ 1 & t\geq t_{m} \end{array}\right.$$

where *I* represents the external inducer and is a constant, *m* represents the expression level of upstream transcription factors, α represents the maximal transcription factor expression rate, K represents the dissociation constant, *K*_1_ is a constant indicating the biological toxicity of cyclopentanone, *δ* is the dilution rate resulting from cell growth, and *t_m_* indicates the time needed for the transcription factor to start expression after induction. By fitting the experimental data, we obtained α  =  884.55 (a.u.), *K*  =  0.058 (mM), which indicated that the transcription factor expression approximately approached α/2 under a cyclopentanone concentration of 0.058 mM, *K*_1_ =  0.6 (mm), which indicated that the transcription factor expression approximately decreased to α/2 under a cyclopentanone concentration of 0.6 mM. *t_m_* fitted here was 18.4 h (Figure S11, Supporting Information), which indicated that the transcription factor started to express after 18.4 h of induction. The growth rate *δ* can be derived by measuring the area change of single cells, and *δ* derived here was ≈0.498 h^−1^.

Based on the experimental data, it was observed that the downstream promoter had minimal expression when the expression of the transcription factor was below a certain threshold. However, when the expression of the transcription factor exceeded the threshold, the expression of the downstream promoters was almost directly proportional to the expression of the transcription factor (Figure 3c). Therefore, a simple mathematical model was constructed to represent this regulatory relationship.

(3) $$\frac{dG}{dt}=\left\{\begin{array}{cc} \beta_{G}-\delta G & m<m_{0}\\ \beta_{G}+f_{G}\left(t\right)\alpha_{G}\left(m-m_{0}\right)-\delta G & m\geq m_{0} \end{array}\right.$$

(4) $$f_{G}=\left\{\begin{array}{cc} 0 & t<t_{G}\\ 1 & t\geq t_{G} \end{array}\right.$$

where *β_G_* is a constant representing the basal expression rate of protein, *α_G_* represents the activation strength and *m*_0_ represents the threshold level of transcription factor expression needed to activate the promoter, and *δ* represents the dilution rate resulting from cell growth, and *t_G_* represents the time required for downstream *sfGFP* to start expressing after activation of the promoter.

The parameters obtained from fitting (Figure S11, Supporting Information) are listed in Figure 3d. Based on these parameters, we can conclude that the promoter *P_PfmaI_* requires the highest threshold to be activated by PfmaH, while *P_PfmaD_* has the lowest threshold. The threshold values required for activation of the promoters can be arranged in the following order:

(5) $$\begin{array}{ccc} m_{o}\left(P_{PfmaI}\right) & & >m_{o}\left(P_{PfmaE}\right)>m_{o}\left(P_{PfmaJ}\right)\\ & & >m_{o}\left(P_{PfmaG}\right)>m_{o}\left(P_{PfmaD}\right) \end{array}$$

While the activation strength of the downstream promoters could be arranged as follows:

(6) $$a_{G}\left(P_{PfmaE}\right)\geq a_{G}\left(P_{PfmaI}\right)>a_{G}\left(P_{PfmaG}\right)\geq a_{G}\left(P_{PfmaJ}\right)\approx a_{G}\left(P_{PfmaD}\right)$$

And the time needed for the promoters to start expression could be arranged as follows:

(7) $$t_{G}\left(P_{PfmaG}\right)>t_{G}\left(P_{PfmaE}\right)>t_{G}\left(P_{PfmaD}\right)>t_{G}\left(P_{PfmaJ}\right)>t_{G}\left(P_{PfmaI}\right)$$

The maximum expression (*G_max_*) relative to the basic value (β_*G*_/δ) could be arranged as follows:

(8) $$\begin{array}{ccc} \frac{G_{max}}{\beta_{G}/\delta }\left(P_{\mathrm{PfmaE}}\right) & & >\frac{G_{max}}{\beta_{G}/\delta }\;\left(P_{\mathrm{PfmaG}}\right)>\;\frac{G_{max}}{\beta_{G}/\delta }\left(P_{\mathrm{PfmaD}}\right)\\ & & \;\geq \frac{G_{max}}{\beta_{G}/\delta }\;\left(P_{\mathrm{PfmaI}}\right)>\;\frac{G_{max}}{\beta_{G}/\delta }\left(P_{\mathrm{PfmaJ}}\right) \end{array}$$

From the data, we can infer that *P_PfmaE_* is the strongest promoter, with a low basic expression but a high maximum expression. On the other hand, *P_PfmaJ_* is the weakest promoter, with the lowest activation strength and a maximum expression near to its basic expression level, suggesting high leakage in expression. *P_PfmaG_* and *P_PfmaD_* have similar strengths to *P_PfmaJ_*, but *P_PfmaG_* is slightly stronger with a longer time required to start expression and the second lowest leakage in expression. Leakage in expression may occur because the promoters are not exclusively regulated by PfmaH, and other factors such as upstream regulators and environmental conditions can influence promoter expression.

**Figure 4:**
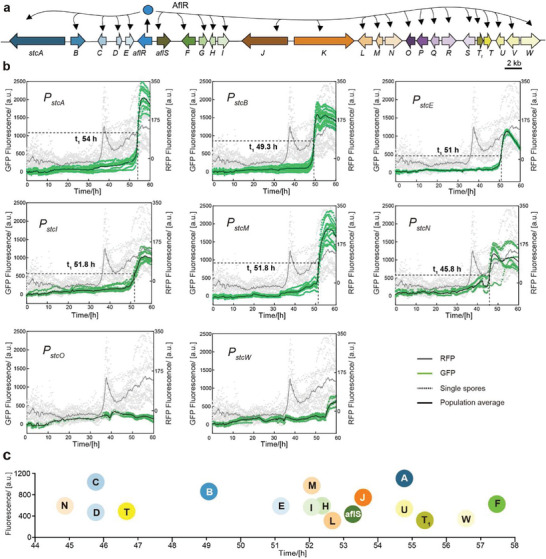
Dynamic analysis and quantification of regulatory elements in the AflR gene regulatory circuit. a) The AflR‐GRC is comprised of two transcription factors and twenty‐five functional genes, with AflR serving as the specific regulator of this circuit. b) Expression data for a portion of the promoter in the AflR‐GRC and another portion of the promoter data was placed in the Figure S12 (Supporting Information). 0.2 mm of cyclopentanone was used to activate AflR. Each dashed line on the graph indicates the fluorescence data for a single cell, while the solid line represents the average of multiple single‐cell fluorescence data over 60 h. The grey part shows the red fluorescence data output which indicates the expression level of AflR, with values reflected on the right *y*‐axis, the sample sizes for all graphs are 14. The green part represents the expression level of the promoter fused with sfGFP, The sample sizes are 10 (*P_stcA_*), 16 (*P_stcB_*), 8 (*P_stcE_*), 12 (*P_stcI_*), 13 (*P_stcM_*), 8 (*P_stcN_*), 8 (*P_stcO_*) and 13 (*P_stcW_*) respectively. t_1_ represents the time point at which the promoter expression reaches half of the plateau under optimal conditions. For some promoters, t_1_ time point was not labeled since they did not show a significant expression increase at 60 h **c)** Differences among promoters in the regulation of gene expression in terms of their expression time and intensity. Some promoters may not show significant increases in expression within the given timeframe of 60 h and, as a result, may not be included in the analysis presented in the graph or other visualizations.

In Figure 3, we can observe that the changes in promoter expression with the addition of 0.2 mm cyclopentanone match the fitted data, and thus 0.2 mm cyclopentanone was chosen as the incubation condition for the quantitative evaluation of the AflR‐GRC (**Figure** **4**a) with 25 genes whose expression is altered by AflR (Figure 4b; Figure S12, Supporting Information). However, unlike the straightforward PfmaH‐GRC, the AflR‐GRC exhibits greater complexity and diversity, with most of the 25 promoters favorably regulated by AflR, but exhibiting varying expression intensity and timing of expression initiation (Figure 4c). This suggests that the regulation of PSTF is more complex in GRCs with more members, confirming why PSTF is frequently found in GRCs with multiple gene members.

### 2.4 Refactoring the Biosynthetic Pathway of Representative Drugs to Test Quantified GRCs

To test the accuracy of our approach, we wonder if these quantified GRCs could be used to control the production of bioactive metabolite precisely. To probe it, we chose to precisely control the titer of beauvericin, an insecticide widely used in agriculture.^[^ ^66^, ^67^ ^]^ The biosynthesis of beauvericin requires a two‐enzyme catalyzed process (Figure S13a, Supporting Information). The first step is that an NADPH‐dependent 2‐ketoisovalerate reductase Kivr converts 2‐ketoisovalerate to D‐2‐hydroxyisovalerate (D‐HIV). Then, a nonribosomal peptide synthetase Beas iteratively catalyzes D‐HIV and L‐phenylalanine (L‐Phe) into a peptidyl alcohol monomer intermediate in a programmed iteration and generates cyclic trimethyl ester beauvericin from this intermediate by an unusual recursive process.^[^ ^68^ ^]^ The simple biosynthetic pathway of beauvericin would be a good model to evaluate the titer by controlling the expression time and intensity of biosynthetic genes.

**Figure 5:**
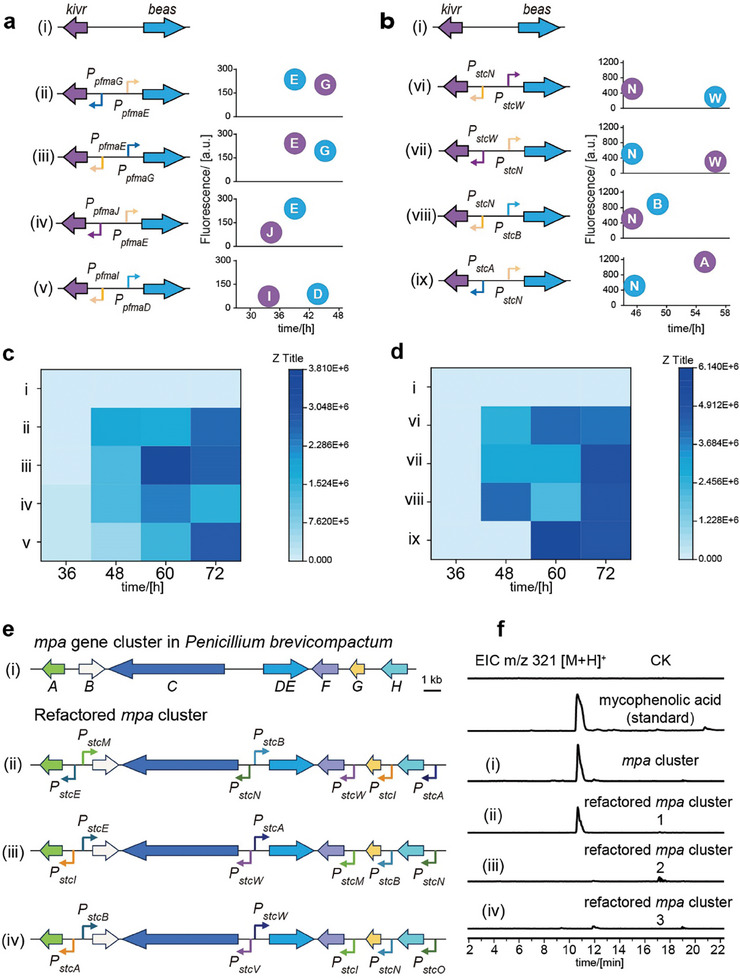
Precise control of beauvericin and mycophenolic acid production a,b) Precise control of beauvericin production by PfmaH‐GRC and AflR‐GRC. We made several combinations based on the timing and intensity of expression of promoters that are regulated by PfmaH and AflR. The promoter strength and expression time in the prediction plots are based on t_1_ in Figures 3b and 4b and Figure S12 (Supporting Information). c) Quantification of beauvericin produced by different promoter combinations from PfmaH‐GRC. i) *kivr* and *beas* with their original promoters. ii) *kivr* and *beas* with promoters *P_pfmaG_* and *P_pfmaE_*. iii) *kivr* and *beas* with promoters *P_pfmaE_* and *P_pfmaG_*. iv) *kivr* and *beas* with promoters *P_pfmaJ_* and *P_pfmaE_*. v) *kivr* and *beas* with promoters *P_pfmaI_* and *P_pfmaD_*. d) Quantification of beauvericin produced by different promoter combinations from AflR‐GRC. (i) *kivr* and *beas* with their original promoters. vi) *kivr* and *beas* with promoters *P_stcN_* and *P_stcW_*. vii) *kivr* and *beas* with promoters *P_stcW_* and *P_stcN_*. viii) *kivr* and *beas* with promoters *P_stcN_* and *P_stcB_*. ix) *kivr* and *beas* with promoters *P_stcA_* and *_PstcN_*. e) The mycophenolic acid gene cluster in *Penicillium brevicompactum* (i) and refactored *mpa* gene cluster. ii) Refactor *mpa* cluster with selected promoters (expression time from early to late). iii) Refactor *mpa* cluster with selected promoters (expression time from late to early). iv) Refactor *mpa* cluster with selected promoters (expression time randomized). f) LC‐MS analysis of the refactored *mpa* cluster. The control strain was TYWL8, which was engineered to disrupt the *wA* locus of *A. nidulans*. This disrupted strain exhibited the same white spore characteristics as the strain that had its mycophenolic acid pathway refactored.

Next, we selected the TF‐promoter combinations to control biosynthetic genes *kivr* and *beas* via the quantified PfmaH‐GRC and AflR‐GRC elements. In the experiment, the expression strength and expression speed (timing) of the promoter will be fully considered, and they will match or cross‐match with each other. The catalytic orders of enzymes are also considered. For PfmaH‐GRC elements, five promoters *P_pfmaD_*_,_ *P_pfmaE_*_,_ *P_pfmaG_*_,_ *P_pfmaI_*_,_ and *P_pfmaJ_* were used (**Figure** **5**a). Combinations of promoters iv (*P_pfmaJ_: :kivr+P_pfmaE_: :beas*), and v (*P_pfmaI_: :kivr+P_pfmaD_: :beas*) exhibited a more rapid onset of expression, with trace amounts of beauvericin being detectable as early as 36 h post‐inoculation. Combination iii (*P_pfmaE_: :kivr+P_pfmaG_: :beas*) was found the highest beauvericin titer in all tests. This result is due to the fact that both promoters are strong and the initiation of expression is consistent with the biosynthetic steps of beauvericin (Figure 5c; Figure S13b, Supporting Information). For AflR‐GRC elements, five promoters *P_stcN_, P_stcW_, P_stcA_*, and *P_stcB_* were selected (Figure 5b). The production of beauvericin was detected at 48 h with the combinations vi (*P_stcN_: :kivr+P_stcW_: :beas*), vii (*P_stcW_: :kivr+P_stcN_: :beas*), and viii (*P_stcN_: :kivr+P_stcB_: :beas*). However, the beauvericin was produced earlier than expected expression time (later than 54 h) in the combinations vi and vii, leading us to suspect the potential involvement of *P_stcW_* leakage (Figure S12, Supporting Information). The combination ix (*P_stcA_: :kivr+P_stcN_: :beas*) yielded the expected beauvericin in the timing and yields which is consistent with the expectation (Figure 5d; Figure S13c, Supporting Information). The results showed that the timing and titer of compound production were generally consistent with promoter strength (*α_G_*) and the time required to start expression (*t_G_*). We also observed an interesting pattern indicating that the production of the target product is faster (*P_stcN_: :kivr+P_stcW_: :beas* compared with *P_stcW_: :kivr+P_stcN_: :beas*) or enhanced (*P_pfmaE_: :kivr+P_pfmaG_: :beas* compared with *P_pfmaG_: :kivr+P_pfmaE_: :beas*) when promoter expression coincides with the biosynthesis step of compound. In addition, we observed that the target product was produced faster when the timing difference in the promoter combination was minimized.

To verify whether the quantified GRCs can be used for the refactoring of multiple‐enzyme (>3) pathways, a gene cluster (*mpa*) of mycophenolic acid with seven genes encoding seven enzymes for its biosynthesis was selected.^[^ ^69^ ^]^ According to the sequence of mycophenolic acid biosynthesis, 7 genes were reconstructed by using promoters (*P_stcN_‐P_stcB_‐P_stcE_‐P_stcM_‐P_stcI_‐P_stcA_*) in AflR‐GRC element with similar expression initiation rate (*mpaC‐mpaDE‐mpaA‐mpaB‐mpaG‐mpaH*) (refactored pathway 1) (Figure S14, Supporting Information). As a control, two other refactored pathways were established, the promoter expression time of this pathway 2 (PstcW‐PstcA‐PstcI‐PstcE‐PstcB‐PstcN) was opposite to the sequence of mycophenolic acid biosynthesis and the promoter in pathway 3 expressed in a random fashion (Figure 5e). The production of mycophenolic acid was detected after 5 days with the refactored pathway 1 but not with refactored pathway 2 and pathway 3 (Figure 5f), indicating the matter of the expression timing of promoters.

### 2.5 Refactoring the Biosynthetic Pathway for Silent Gene Cluster Activation and Novel Natural Production Using Quantified GRCs

A PSTF usually is required for the regulation and activation of a biosynthetic gene cluster. However, about 50% of the gene clusters in the fungus lack PSTF,^[^ ^34^ ^]^ which makes the product difficult to identify. To evaluate the potential application of quantified GRCs in the activation of silent gene clusters in fungi, we chose a previously reported silent gene cluster, which encodes immunosuppressant neosartoricin.^[^ ^70^ ^]^ The cluster was only activated by the overexpression of the original PSTF NscR. We refactored *nsc* gene cluster without NscR by using AflR‐GRC elements, *P_stcA_*, *P_stcN_*, *P_stcI_*, and *P_stcW_* to control four biosynthetic genes, *nscA, nscB, nscC*, and *nscD*, respectively. The resulting biosynthetic gene cluster was then transformed into an *A. nidulans* host. LC‐MS analysis showed that neosartoricins B and C were successfully synthesized by refactored pathway (Figures S15 and S16, Supporting Information).

**Figure 6:**
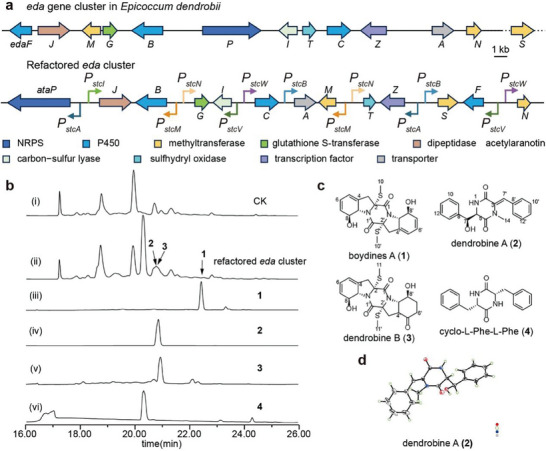
Refactoring the *eda* pathway using AflR‐controlled promoters. a) The *eda* gene cluster in *Epicoccum dendrobii* and the refactored *eda* gene cluster that fused with AflR‐controlled promoters. b) HPLC analysis of metabolic profiles at 254 nm, including i) TYWL8 (CK), ii) strain expressing refactored *eda* gene cluster, and isolated compounds 1, 2, 3, (iii to v) and standard compound 4 (vi). Compounds 1, 2, and 3 were characterized by NMR analysis. c) Structures of the identified compounds. 2 and 3 are new compounds. d) X‐ray crystallographic structure of 2.

Next, we expand the application of quantified GRC for the identification of gene clusters with unknown products. An endophytic fungus *Epicoccum dendrobii* which can lead to widespread alteration of secondary metabolism during cocultivation with other filamentous fungi, cause our attention.^[^ ^71^ ^]^ Analysis of the biosynthetic potential of *E. dendrobii* revealed a biosynthesis gene cluster is similar to the *A. fumigatus* virulence factor gliotoxin, which is a class of epipolythiodioxopiperazines (ETPs) analogs with a diketopiperazine (DKP) as the core skeleton and also containing a sulfur bridge.^[^ ^72^, ^73^, ^74^, ^75^, ^76^ ^]^ However, some of the biosynthetic genes between these two clusters are different (**Figure** **6**a; Figure S17 and Table S6, Supporting Information), indicating the potential new ETP product production. The cluster was named as *eda* gene cluster including 12 genes that were annotated to contain a nonribosomal peptide synthetase (EdaP), 2 cytochrome P450 (EdaF, and EdaC), 2 methyltransferases (EdaM, and EdaN), a major facilitator superfamily (MFS) transporter (EdaA), a Zn_2_Cys_6_ transcription factor (EdaZ), a dipeptidase (EdaJ), a glutathione S‐transferase (EdaG), a carbon−sulfur lyase (EdaI), and a sulfhydryl oxidase (EdaT). To refactor this biosynthetic pathway, we first expressed the core gene (EdaP) in the heterologous host *A. nidulans* and no product was detected either with the quantitative promoter *P_stcA_* or the constitutive promoter *P_gpdA_*. Therefore, we chose another NRPS encoding gene *ataP* from *A. terreus*,^[^ ^77^ ^]^ which is 37% identity to this gene. Meanwhile, two methyltransferase genes, *gtmA* and *ataS*, were respectively present in the genomes of *A*. *fumigatus* and *A*. *terreus* out of cluster, and their homologous gene, *edaS* was also found in *E*. *dendrobii*.

Totally, we refactored 13 genes by using AflR‐GRC elements (Figure 6a). RT‐PCR verified the expressions of all 13 genes (Figure S18, Supporting Information) and several new peaks were found in engineered *A. nidulans* (Figure 6b,c,d). To identify these metabolites, a scale‐up culture of *A. nidulans* strain with engineered *eda* BGC was performed in 5 kg of rice medium for 10 days. Three compounds were isolated from the crude extracts (Figure 6b,c) and their structures were elucidated by HR‐ESI‐MS, NMR and X‐ray crystal diffraction (Tables S7–S9 and Figures S19–S46, Supporting Information). Compound **1** was elucidated as known boydines A.^[^ ^78^ ^]^ Compound **2**, named dendrobine A, was elucidated as the derivative of diphenylalazine. The structure of **2** was further confirmed via X‐ray crystallographic analysis (CCDC 2 297 622) (Figure 6d). Compound **3**, sharing the same skeleton as boydines A, was established validly and named dendrobine B. Compounds **2** and **3** are novel natural products.

## 3 Discussion

The ability to precisely control genetic circuits is essential for manipulating gene expression and the construction of synthetic biology systems. However, quantifying gene expression in specific cellular processes is often challenging, especially in complex multicellular organisms such as fungi. Microfluidics has previously been used for filamentous fungi^[^ ^79^ ^]^ to establish platforms for detecting pathogenic process detection,^[^ ^80^ ^]^ identifying metabolites,^[^ ^81^, ^82^ ^]^ and manipulating genes on a high‐throughput basis.^[^ ^83^ ^]^ Here, we introduce a state‐of‐the‐art microfluidic platform for characterizing the gene regulatory circuits involved in the biosynthesis of secondary metabolites in multicellular fungi at the single‐cell level. Our microfluidic platform offers several advantages over traditional methods of gene expression analysis. First, it enables the precise measurement of gene expression levels in complex multicellular fungi, which is not possible using conventional methods such as bulk RNA sequencing.^[^ ^84^, ^85^, ^86^ ^]^ Second, our platform allows for the analysis of multiple regulatory combinations simultaneously, which provides a more comprehensive understanding of gene regulatory circuits. Finally, our approach offers high throughput, enabling the analysis of large datasets in a short amount of time. Additionally, the customized microfluidic chips were securely affixed for single‐cell monitoring, and fluorescence data were collected in real‐time for up to 60 h (Figure 2c). This makes our platform highly suitable for evaluating biosynthetic genes involved in producing valuable compounds.

By utilizing a microfluidic platform, we were able to quantify two representative GRCs involved in the biosynthesis of sterigmatocystin and melanin in *A. nidulans*. The fluorescent monitoring and mathematical model enabled us to determine the expression data of 30 regulatory combinations from the two GRCs. This data provided us with more comprehensive insights into how TFs control the gene expressions of their members in the PfmaH‐melanin pathway or AflR‐sterigmatocystin pathway. Using precisely quantified GRCs, we were able to precisely control biosynthesis and titer of drug molecules, beauvericin, and mycophenolic acid. In our study, we found that the probability of successful compound production is increased when the promoter initiation time in the combination is closer to each other. We further observed that the probability of successfully creating a compound is higher when the promoter initiation time is used in alignment with the biosynthetic steps of the compound. Next, we tried to apply quantified GRCs to refactored clusters of unknown products. By utilizing a combination of AflR‐GRC elements, we were able to successfully activate the *nsc* and *eda* biosynthetic pathway (Figure 6; Figures S15–S19, Supporting Information). This outcome implies that quantitative GRCs could be a valuable resource for the activation of silent gene clusters, particularly because nearly half of all fungal BGCs lack a PSTF in nature. In this research process, it is crucial to delve deeper into factors such as precursor toxicity and bottleneck pathways, which require additional studies.

In summary, this study demonstrates the potential of microfluidic platforms for investigating gene quantification in complex multicellular fungi, and highlights the importance of controlled gene expression in the production of high‐value‐added metabolites using filamentous fungal cell factories. Our findings provide a valuable resource for researchers interested in studying and manipulating gene expression in multicellular fungi and offer new opportunities for developing innovative approaches in drug development and synthetic biology.

## 4 Experimental Section

### Sequences, Strains, Plasmids, and Growth Conditions

All transcription factors and promoters that were used were obtained from public databases and can be searched in the National Center for Biotechnology Information (NCBI). AflR gene regulatory circuit sequences were obtained from the genome of Aspergillus nidulans FGSC A4 (AN7804‐AN7825), and PfmaH gene regulatory circuit sequences were derived from the genome of strain Pestalotiopsis fici W106‐1 (PFICI_07100‐07104, PFICI_0 2498 and PFICI_0 5460).

The plasmids and strains used in this study are listed in Tables S1–S4 (Supporting Information). Oligonucleotides used in this work are listed in Table S5 (Supporting Information). The oligonucleotides were ordered from Sangon Biotech. Both Q5 high‐fidelity DNA polymerase (New England Biolabs) and TransStart FastPfu DNA polymerase (Transgene Biotech) were used for PCR. 2 × TSINGKE Master Mix (TSINGKE) was used for PCR transformation screening. A Zymoclean Gel DNA Recovery Kit (D4007, ZYMO RESEARCH, USA) was used to purify amplified fragments. The restriction enzymes used in this study were purchased from New England Biolabs. Plasmids were extracted using the E.Z.N.A. Plasmid Midi Kit (OMEGA) or Zymoprep (D2001) Kit (Zymo Research). Nano DropH Spectrophotometer ND‐2000 (Peqlab, Erlangen, Germany) was used to measure the quantities of plasmids.

*Escherichia coli* strains DH5α and DH10B were used for plasmid construction and enrichment. All *E. coli* strains were cultured in LB medium containing the relevant antibiotics at 37 °C. *Saccharomyces cerevisiae* strain BJ5464‐NpgA (*MATα ura3‐52 his3‐Δ200 leu2‐Δ1 trp1 pep4: :HIS3 prb1Δ1.6R can1 GAL*) was cultured on yeast extract peptone dextrose medium (YPD). After transformation, *S. cerevisiae* was selected on synthetic dextrose complete medium (SDCt) with auxotrophic marker‐specific supplements. *Aspergillus nidulans* LO8030 strain was the recipient host for transformation experiments.^[^ ^87^, ^88^, ^89^ ^]^ Sorbitol minimum medium (SMM) and glucose minimal medium (GMM) were cultured for sporulation and transformation at 37 °C with the addition of 0.56 g uracil L^−1^, 1.26 g uridine L^−1^, 0.5 µm pyridoxine HCl, and 2.5 µm riboflavin. Preparation and transformation of fungal protoplasts were performed as described by Yin et al.^[^ ^70^ ^]^ Transformants were validated by diagnostic PCR using the proper primers (Table S5, Supporting Information). *Pestalotiopsis fici* CGMCC3.15140 was cultivated at 25 °C in PDA or potato dextrose broth (PDB). For induction of *P_alcA_* in liquid medium, lactose minimal medium (MM, 20 g L^−1^ lactose, 6 g L^−1^ NaNO_3_, 0.52 g L^−1^ KCl, 0.52 g L^−1^ MgSO_4_·7H_2_O, 1.52 g L^−1^ KH_2_PO_4_, and 1 mL L^−1^ trace element solution supplemented with cyclopentanone at the concentrations indicated in the figure legends) were used. For induction of *P_alcA_* on a solid medium, the inducer was replaced with 3.1 mm threonine, and 16 g L^−1^ agar was added. Repressing solid media were YAG (5 g L^−1^ yeast extract, 20 g L^−1^ D‐glucose, 16 g L^−1^ agar, and 1 mL L^−1^ trace element solution).

### Dual‐Fluorescence Labeled *Aspergillus nidulans* Strain Construction

Two fluorescent tags, mCherry and sfGFP, were fused with transcription factors and promoters in GRC, respectively (Table S3, Supporting Information). To build the insertion cassettes of AflR‐GRC, a yeast recombination strategy^[^ ^90^ ^]^ was utilized to assemble the upstream 1.03 kb fragment and the downstream 1.1 kb fragment of the ST gene cluster (AN7804‐AN7825), *P_alcA_* (0.47 kb), *aflR* (1.3 kb), *mCherry* (0.711 kb), *T_trpC_* and *AfpyroA* (2.8 kb) into the plasmid pYXR121. The complete DNA fragment was subsequently amplified and transformed into *A. nidulans* to generate TYXR111. To generate dual‐fluorescence labeled strains of PfmaH‐GRC, yeast recombination was utilized to assemble *AfRibo*, *PalcA*, *pfmaH*, and *mCherry* into plasmid pYXR84, which was subsequently purified for transformation into *A. nidulans* strain LO8030 to yield TYXR113. To produce fusion plasmids of promoters and *sfGFP*, 30 promoters from the two GRCs were amplified and recovered and then put into plasmid pYZM8 with *sfGFP* using the Quick‐change method.^[^ ^91^ ^]^ These plasmids were validated by endonuclease digestion and linearized by SwaI for fungal transformation. Receiving hosts were TYXR111 and TYXR113, respectively, to generate a series of strains with two fluorescent labels (TYXR74‐TYXR79, TYXR84‐TYXR108). Plasmids pYPZ78 and pYPZ79 were transformed into host TYXR27, to generate two strains labeled with sfGFP only.

The dual‐fluorescence strains were cultured in a medium supplemented with the inducer in a shaking flask for three days (Figure S3a, Supporting Information). As expected, both the red and green fluorescence of the dual‐fluorescence strain were illuminated. In the control strains, green fluorescence was observed, indicating that *A. nidulans* produces some of its own green fluorescence; therefore, the fluorescence value of this component should be subtracted from the data to avoid interference (Figures S3b and S4a, Supporting Information). When cultured in microfluidic chips, conditioned media (CM) was generally added to mimic the natural growth environment of population cells (Figure S3b, Supporting Information). CM was the MM medium after culturing *A. nidulans* LO8030 for a period of time and filtering out the mycelium. When pre‐experiments were performed with TYXR75 as an example, it is found that the adding of CM is necessary for the induction of the transcription factor. A 20% conditioned media was chosen for microfluidic induction experiments based on previous experiment results (Figure S4b, Supporting Information).

### Microfluidic Device and Microscope Set‐Up

Standard three‐layer lithography with Su8 photoresist (MicroChem, US) was utilized to produce the chip mold. Standard soft‐lithography methods were used to produce the PDMS layer. The cell‐loading wells and medium inlets were punched using a sharp metal puncher with a 1 mm diameter after the hardened PDMS layer (6–8 mm thickness) was scraped from the mold. Then, the PDMS layer was bonded to a specially ordered coverglass (24 mm × 60 mm × 0.17 mm) using air plasma.

Dual‐fluorescence labeled strains were inoculated on SMM plates and cultured at 37 °C for four days, and then the conidia were collected with 1% Tween‐H_2_O. Using a blood‐counting plate, the number of conidia per mutant was controlled at 1 × 10^8^ mL^−1^. Five hundred microliters of spore solution (1 × 10^8^ mL^−1^) were added to 3 mL of lactose minimum medium (MM) supplemented with 0.1% pyridoxine hydrochloride. To increase their size, the first 4 h were spent growing them in the test tube with MM. To place the conidia on the chip, tips were used to put them in their respective channels. The conidia were loaded into the corresponding channel on the chip using tips. The medium used on the chip was MM supplemented with cyclopentanone and conditional medium. The temperature of the microscope platform was set to 37 °C. The flow control program written in MATLAB software was used to edit the running time and flow rate of each injection pump. The injection time was set to 60 h, and the flow rate was set to 150 µL h^−1^.

The time‐delay imaging experiment utilized a Nikon Ti‐E inverted fluorescence microscope with an EMCCD camera (Andor iXon×3 DU897) and a CFI Plan Apochromat Lambda DM 60× oil‐immersed objective (NA 1.40 WD 0.13 mm). NIS‐Elements advanced research software was used to select observation points for each culture chamber, and six points were selected for each strain with different genotypes. Each culture chamber should contain at least two conidia, and the overall conidia should range between 10 and 30. The images were taken with a 15 min resolution at each observation site. To ensure that fluorescence irradiation would not interfere with cell development over an extended period, exposure time, intensity, and camera gain were adjusted to be the same for each study batch. The custom microfluidic chip for filamentous fungi can simultaneously load up to eight strains at a time.

### Microfluidic Data Analysis

Time‐lapse microscopy was used to capture time series images of spore growth, and ImageJ and MATLAB software were used to quantitatively interpret the data. Using software to bring computing function to obtain the circle spore image average fluorescence intensity $\bar{I}$, all photographs were captured in the same spore circle within 60 h (241 images), and the average fluorescence intensity curve was obtained. It is vital to manually circle the spore position on each photograph to prevent data loss due to spore movement, as conidia may move during growth. Due to the background fluorescence of *A. nidulans*, the average of spore fluorescence data was used from 10 control strains as the reference value for *A. nidulans* (Figure S4a, Supporting Information). This value was classified as background fluorescence *I_b_* for this fungus. Spore fluorescence intensity was quantified as follows:

(9) $$I=\bar{I}-I_{b}$$

Fluorescence data was collected from at least 8 single conidia within 60 h for each dual‐fluorescence strain, sorted the maximum fluorescence value of each spore during this period, and plotted fluorescence data of strains within ±25% of the median. Data from multiple identical conditions were averaged. Normalization was performed on the conditional exploration part data, and fluorescence data of five adjacent time points were averaged for plotting. For transcription factor data in the same GRC, RFP data from multiple dual‐fluorescence labeled strains were processed together. Data were analyzed using Origin Pro 2021 and GraphPad Prism 8.

### Fungal MoClo DNA Assembly Tool‐Box Construction

In order to seamlessly assemble diverse types of biological elements, the MoClo assembly approach taken in *E. coli^[^* ^62^, ^63^ ^]^ to develop a DNA assembly toolbox (Figures S1 and S2, Table S1, Supporting Information) suitable for filamentous fungal hosts, including a library of GRC promoter elements and vectors optimized for multistage assembly was resorted. MoClo is derived from the Golden Gate cloning process, in which type IIS endonucleases are utilized to cut outside of their recognition sequence and can yield any potential 4‐bp sticky ends. These sticky ends can then anneal and ligate with their respective sticky ends, resulting in the appropriate order of DNA assembly.

The promoters of GRC were attached as basic elements to a specific level 0 vector. All level 0 destination vectors were pUC19‐based, conferred ampicillin resistance (*Amp^R^*), and encoded a *lacZα* fragment for blue/white selection. For Bsa I restriction site on *Amp^R^* gene, synonymous mutation of GGTCTC into GGTACC by Quick change method. The recognition sites of Bsa I and Bbs I were positioned on each side of the *lacZα* segment, and cleavage sites were designed to be complementary to the promoter cleavage sites. For cloning level 0 modules, the prescribed sequences were PCR‐amplified with the corresponding fusion site and a Bbs I recognition site as part of the amplification primers and then cloned using the Golden Gate cloning procedure. During this phase, any internal type IIS recognition site for enzymes used in the MoClo method (BsaI, BbsI, and eventually Esp3I) can be deleted from the cloned fragment by using primers that overlap but contain a single silent nucleotide mismatch within the recognition site. Since all level 0 modules of the same kind were bordered by identical fusion sites, they were freely interchangeable, enabling the creation of any transcription unit by simply selecting the required modules.

Compatible sets of sequenced level 0 modules were combined into a level 1 destination vector using a second Golden Gate reaction involving the enzyme Bsa I. In contrast to level 0 modules, level 1 destination vectors were constructed with pET28a backbone and confer kanamycin resistance (Kan^R^), providing efficient counter selection against the level 0 module backbone. The type IIS recognition sites on pET28a were altered similarly to the level 0 destination vectors. The matching type IIS recognition sites (Esp 3I and BsaI) and fusion sites were added to the level 1 vectors. According to the design guidelines of the Molco method, 7 level 1 destination vectors with BbsI restriction sites that generate two fusion sites with new specificities were designed. Due to this design, a maximum of 6 transcription units can be cloned in a single step. The combination of the two sets permits the cloning of transcription units in either orientation at any position in level 2 constructs.

A set of seven level 2 destination vectors was constructed using XW55 backbone modified to impart chloramphenicol resistance (Cm^R^), the matching type IIS recognition sites (BsaI and Esp 3I), and fusion sites. To assemble multiple level 2 vectors and express them in *A. nidulans*, 3 end‐linker vectors and a suitable vector for *A. nidulans* pYH‐wA were also modified. End‐linker vectors provide kanamycin resistance, similar to level 1 modules, and are flanked by Bsa I sites. Bsa I was then used to assemble the desired multigene express vectors from the chosen level 1 modules, a corresponding end‐linker, and a level 2 destination vector. All fusion sites used in this study were selected based on information published by Mohammad Hamedi Rad et al. in 2019.^[^ ^64^ ^]^ The fusion sites have a high level of specificity, a more significant proportion of successful assembly, a greater affinity, and a greater number of plate colonies.

### Heterologous Expression of Refactored Beauvericin Biosynthetic Pathway

Two genes, *beas* and *kivr*, are required for the biosynthesis of beauvericin. Based on the results of the microfluidics, the PfmaH‐GRC and AflR‐GRC elements were ordered in time and intensity, and made the following eight combinations: including *P_stcN_‐P_stcW_*, *P_stcW_‐P_stcN_*, *P_stcN_‐P_stcB_*, *P_stcA_‐P_stcN_*, *P_pfmaE_‐P_pfmaG_*, *P_pfmaG_‐P_pfmaE_, P_pfmaJ_‐P_pfmaE_* and *P_pfmaI_‐P_pfmaD_*. Each set of promoters was fused to *kivr* and *beas*, respectively, and all the vectors were constructed by yeast assembly.

### Heterologous Expression of Refactored *mpa* Gene Clusters

A total of 7 genes within *mpa* gene cluster need to be fused with AflR‐GRC promoters. The yeast assembly method was used for the construction of mycophenolic acid biosynthetic pathway reconstruction vectors. pYLSJ5 and pYLSJ6 can be integrated in the *wA* locus at the same time by using the homology arm 15‐HA. pYLSJ5 fused *mpaA*, *mpaB*, *mpaC*, and *mpaDE* to *P_stcE_*, *P_stcM_*, *P_stcN_*, and *P_stcB_*, pYLSJ6 fused *mpaF*, *mpaG*, and *mpaH* to *P_stcW_*, *P_stcI_*, and *P_stcA_*, respectively. Two vectors were subsequently transformed simultaneously into a constitutive *A. nidulans* host that had overexpressed *aflR* to obtain TYLSJ11. pYLSJ7 and pYLSJ8 can be integrated into the *wA* locus at the same time by using the homology arm 15‐HA. pYLSJ7 fused *mpaA*, *mpaB*, *mpaC*, and *mpaDE* to *P_stcI_*, *P_stcE_*, *P_stcW_*, and *P_stcA_*, pYLSJ8 fused *mpaF*, *mpaG*, and *mpaH* to *P_stcM_*, *P_stcB_*, and *P_stcN_*, respectively. Two vectors were subsequently transformed simultaneously into a constitutive *A. nidulans* host that had overexpressed *aflR* to obtain theTYLSJ12.

### Heterologous Expression of Refactored *nsc* Gene Clusters

For the construction of *nsc* refactoring gene cluster expression vectors (Figure S14, Supporting Information), the DNA assembly toolbox was used in steps (Figures S1 and S2, Table S1, Supporting Information), starting with the construction of the first level vector. The four functional genes in the *nsc* gene cluster were combined with promoters *P_stcA_*, *P_stcW_*, *P_stcI_*, and *P_stcN_* in AflR‐GRC according to gene orientation to obtain the level 1 vectors pYXR114, pYXR115, pYXR116, and pYXR117. Then, four level 1 vectors were linked end‐to‐end by type IIS restriction endonuclease Esp 3I and T4 ligase to form level 2 vector pYXR119. Level 2 vector and ligating vector were ligated to level 2i recipient vector in the presence of type IIS restriction endonuclease and T4 ligase. This vector carries the homologous arm of the *A. nidulans wA* locus and is integrated into the chromosome to form strain TYXR115. The control strains were TWY1.1,^[^ ^70^ ^]^ which overexpressed PSTF in the *nsc* gene cluster, and TYXR114, which only expressed four functional genes of *nsc* cluster.

### Heterologous Expression of Refactored *eda* Gene Clusters

A total of 13 genes within *eda* gene cluster need to be fused with AflR‐GRC promoters, and split them into three vectors for assembly. pYLSJ1 and pYXR210 can be integrated in the *wA* locus at the same time by using the homology arm 15‐HA, and the backbone of pYXR209 was the AMAI that can be replicated autonomously in *A. nidulans*. pYLSJ1 fused *ataP* to *P_stc_*_A_, pYXR210 fused *edaJ*, *edaB*, *edaG*, *edaI*, *edaC*, and *edaA*, to *P_stcI_*, *P_stcM_*, *P_stcN_*, *P_stcV_*, *P_stcW_*, and *P_stcB_*, respectively, and pYXR209 in which the skeleton was composed of *P_stcM_*, *P_stcN_*, *P_stcA_*, *P_stcB_*, *P_stcV_*, and *P_stcW_* to activate *edaM*, *edaT*, *edaZ*, *edaS*, *edaF*, and *edaN*, respectively. Three vectors were subsequently transformed simultaneously into a constitutive *A. nidulans* host that had overexpressed *aflR* to obtain the recombinant eda gene cluster in strain TYXR280.

### Transcriptional Analysis by RT‐PCR

For RNA extraction, *A. nidulans* with reconfigured *eda* gene clusters (TYXR280) were shaken in 30 mL LMM medium at 25 °C for 3 days. Total RNA was isolated using TRIzol (TransGen Biotech, China) reagent. Reverse transcription of 1 mg of RNA was performed with the TIANScript II RT Kit (TIANGEN, China). β‐actin gene (AN6542) from *A. nidulans* served as an internal standard. The oligonucleotide sequences for PCR primers are given in Table S5 (Supporting Information).

### HPLC and LC‐MS Analysis of Secondary Metabolites

HPLC analyses were conducted on a Waters HPLC system (Waters e2695, Waters 2998, Photodiode Array Detector) with an ODS column (C18, 250 × 4.6 mm, YMC Pak, 5 um) with a flow rate of 1 mL min^−1^. LC‐MS analysis was performed on an Agilent HPLC 1200 series system equipped with a single quadrupole mass selective detector and an Agilent 1100LC MSD model G1946D mass spectrometer by using a Venusil XBP C18 column (3.0 by 50 mm, 3 µm, Bonna‐Agela Technologies, China). Water (A) with 0.1% (v/v) formic acid and ACN (B) were used as the solvents at a flow rate of 0.5 mL min^−1^. The substances were eluted with a linear gradient from 5 to 100% B in 30 min, then washed with 100% (v/v) solvent B for 5 min, and equilibrated with 5% (v/v) solvent B for 10 min. The mass spectrometer was set in electrospray positive ion mode for ionization. For secondary metabolite analysis of the reconstructed biosynthesis pathway of neosartoricin,^[^ ^70^ ^]^ *A. nidulans* strains were cultivated at 25 °C in 25 mL liquid LMM (supplemented with appropriate supplement) per plate. After 4 days of static submerged culture, 20 mL of each strain's culture was harvested and extracted with 20 mL of ethyl acetate/methanol/acetic acid (89:10:1). For HPLC analysis, the organic phase was dried in a vacuum and re‐dissolved in 200 µL methanol. HPLC analysis was performed at a flow rate of 1 mL min^−1^ and a linear gradient of 20–100% (0–20 min), 100% MeOH (20–25 min), 20% MeOH (25.1–30 min). For secondary metabolite analysis of the reconstructed biosynthesis pathway of beauvericin, *A. nidulans* strains were cultivated at 25 °C in 100 mL liquid LMM (supplemented with appropriate supplement). Starting at 36 h, 20 mL was taken from each 100 mL of culture solution and extracted with an equal amount of ethyl acetate. For HPLC analysis, the organic phase was dried in a vacuum and re‐dissolved in 100 µL methanol. UV absorptions at 210 nm are illustrated. The peak area of beauvericin was used as relative quantitative data for heatmapping. Three parallels were made for each sample. For secondary metabolite analysis of the reconstructed biosynthesis pathway of mycophenolic acid, *A. nidulans* strains were cultivated at 25 °C in 10 g rice for 5 days, and then extracted with 20 mL of ethyl acetate: dichloromethane: methyl alcohol = 3:2:1 (add 1% formic acid). HPLC analysis was performed at a flow rate of 1 mL min^−1^ and a linear gradient of 20–100% (0–20 min), 100% ACN (20–25 min), 20% ACN (25.1–30 min). UV absorptions at 300 nm are illustrated. For secondary metabolite analysis of the strains with reconstructed eda cluster, *A. nidulans* strains were cultivated at 25 °C in rice medium for 5 days, and then extracted with 20 mL of ethyl acetate. HPLC analysis was performed at a flow rate of 1 mL min^−1^ and a linear gradient of 20–100% (0–20 min), 100% ACN (20–25 min), 20% ACN (25.1–30 min).

### Large‐Scale Fermentation, Extraction, and Isolation of Secondary Metabolites

To isolate **1** to **3**, *A. nidulans* with reconfigured *eda* gene cluster (TYXR276) was inoculated into 100 × 500 mL flask containing 50 g rice at 25 °C for 10 days. The cultures were extracted with equal volumes of ethyl acetate for three times and evaporated under reduced pressure to give a crude extract (30.00 g). Subsequently, the crude extract was subjected to silica gel column chromatography and eluted withpetroleum ether, CHCl_2_: MeOH (1:0, 250:1, 100:1, 50:1, 25:1, 10:1, 2:1, and 0:1 in gradient, v/v) to give nine fractions. All fractions were analyzed by LC‐UV, which showed that differentiated compounds were mainly enriched in Fr. 5. Fr. 5 (1.3 g), was further purified by semi‐preparative HPLC (50% CH_3_CN in H_2_O) to yield **1** (6.4 mg), **2** (8.9 mg) and **3** (8.5 mg).

### NMR Analysis

NMR spectra were recorded on a Bruker Avance‐500 MHz spectrometer at room temperature (Bruker Corporation, Karlsruhe, Germany). All spectra were processed with MestReNova 12.0.3 (Metrelab). Chemical shifts are referenced to those of the solvent signals. NMR data are given in Tables S7–S9 (Supporting Information) and spectra in Figures S20–S46 (Supporting Information).

## Conflict of Interest

The authors declare no conflict of interest.

## Author Contributions

X.X., Y.S., and A.Z. contributed equally to this work. W.‐B. Y., C.X. L., and X. X. conceived and designed the project and wrote the manuscript. C.X. L. designed the microfluidic chip. Y. S. made the microfluidic chip. A. Z. contributions to the isolation and structural elucidation of compounds. X. X. designed all the vectors and strains. S. L. and S. Z. were involved in vector construction and genetic manipulation. X. X., Y. S., and S. C. performed microfluidic experiments. C.X. L and Y. S. constructed the mathematical model. C.B. L., L. C., and Y. C. provided guidance for data collection and revised the manuscript. All authors read and approved the final manuscript.

## Supporting information



Supporting Information

## Data Availability

The data that support the findings of this study are available in the supplementary material of this article.
