# Supplementary material for: Quantitative Characterization of Gene Regulatory Circuits Associated With Fungal Secondary Metabolism to Discover Novel Natural Products
Source: Adv Sci (Weinh). 2024 Oct 28;11(47):2407195. doi: 10.1002/advs.202407195 (PMC11653720; doi:10.1002/advs.202407195)
Supplement: Supplementary file 1 — Supporting Information [file ADVS-11-2407195-s001.pdf]

ADVANCED SCIENCE
Open Access

Supporting Information

for *Adv. Sci.*, DOI 10.1002/advs.202407195

Quantitative Characterization of Gene Regulatory Circuits Associated With Fungal Secondary Metabolism to Discover Novel Natural Products

Xinran Xu, Yanhong Sun, Anxin Zhang, Sijia Li, Shu Zhang, Sijing Chen, Chunbo Lou, Lei Cai, Yihua Chen, Chunxiong Luo and Wen-Bing Yin**

Supporting Information

Quantitative Characterization of Gene Regulatory Circuits Associated with Fungal Secondary Metabolism to Discover Novel Natural Products

Xinran Xu[1,2]†, Yanhong Sun[3,4]†, Anxin Zhang[1,2]†, Sijia Li[1], Shu Zhang[1], Sijing Chen[4], Chunbo Lou[5], Lei Cai[1], Yihua Chen[2, 6], Chunxiong Luo[3,4,7], Wen-Bing Yin [1, 2]**

Xinran Xu, Anxin Zhan, Sijia Li, Shu Zhang, Lei Cai, Wen-Bing Yin
State Key Laboratory of Mycology, Institute of Microbiology, Chinese Academy of Sciences, Beijing, PR China
E-mail: yinwb@im.ac.cn, ORCID: 0000-0002-9184-3198

Xinran Xu, Yihua Chen ,Wen-Bing Yin
Medical School, University of Chinese Academy of Sciences, Beijing, PR China.

Yanhong Sun, Chunxiong Luo
Center for Quantitative Biology, Academy for Advanced Interdisciplinary Studies, Peking University, Beijing, PR China.
E-mail: pkuluocx@pku.edu.cn, ORCID: 0000-0001-8974-7693

Yanhong Sun, Sijing Chen, Chunxiong Luo
The State Key Laboratory for Artificial Microstructures and Mesoscopic Physics, School of Physics, Peking University, Beijing, PR China

Chunbo Lou
CAS Key Laboratory of Quantitative Engineering Biology, Shenzhen Institute of Synthetic Biology, Shenzhen Institutes of Advanced Technology, Chinese Academy of Sciences, Shenzhen 518055, China.

Yihua Chen
State Key Laboratory of Microbial Resources, Institute of Microbiology, Chinese Academy of Sciences, Beijing, 100101, PR China.

Chunxiong Luo
Wenzhou Institute University of Chinese Academy of Sciences, Wenzhou, Zhejiang 325001, PR China.

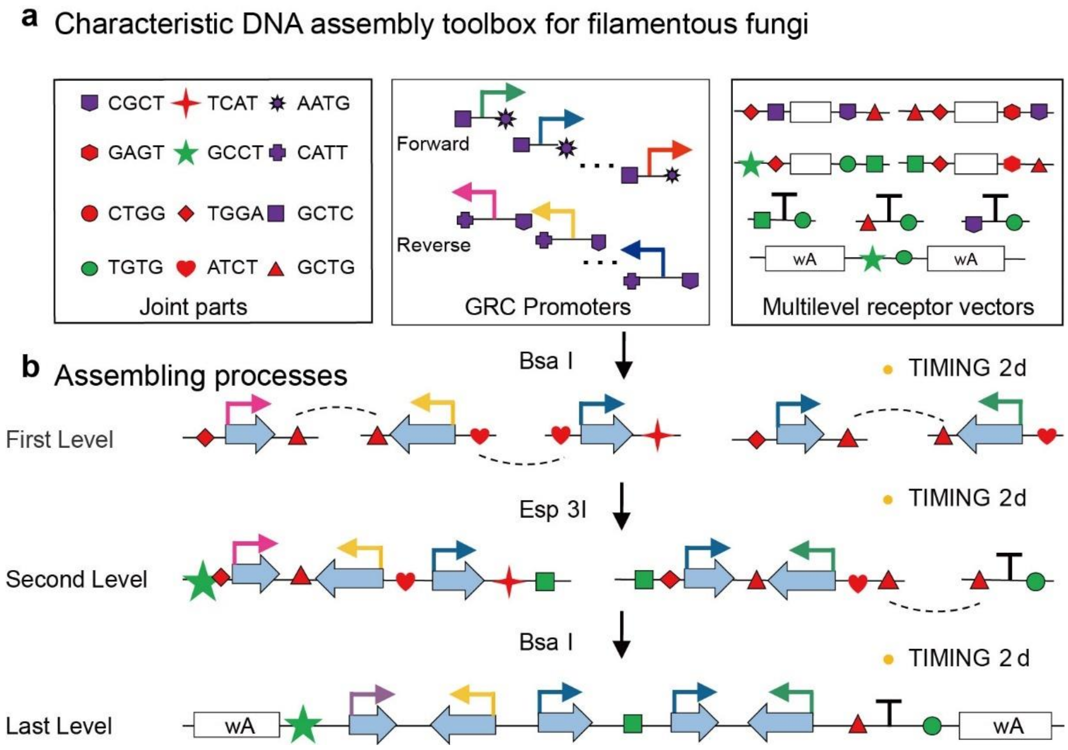

Figure S1. Characteristic DNA assembly toolbox for filamentous fungi. Method creation and joint selection refer to previous methods applications in prokaryotes[62-64].

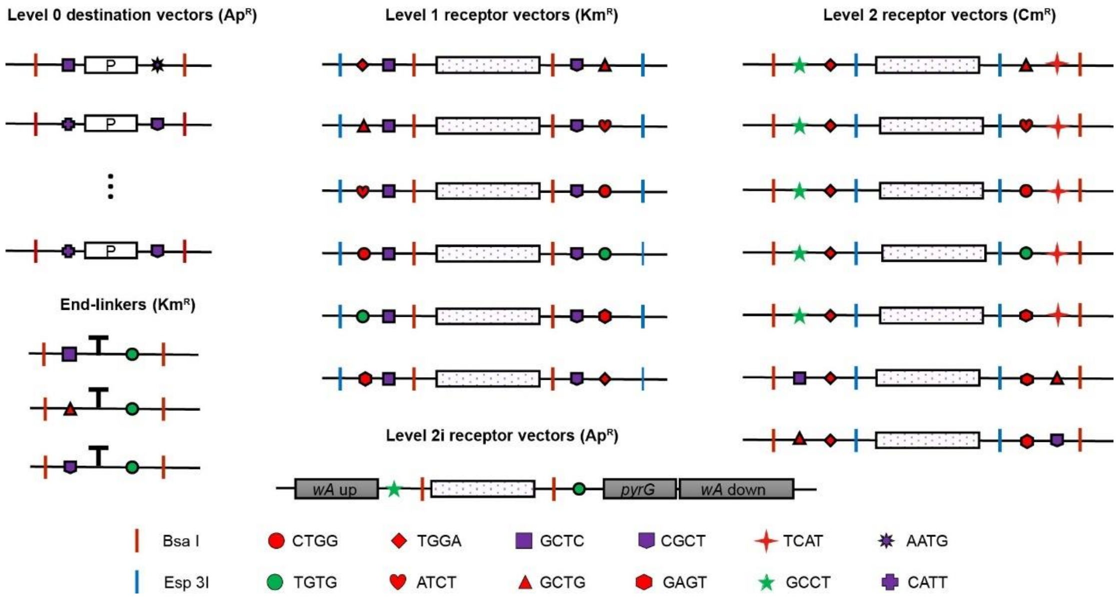

Figure S2. Vectors constructed for DNA assembly of genetic elements of filamentous fungi in this study. The DNA assembly toolbox includes 30 level 0 destination vectors, 7 level 1 receptor vectors, 8 level 2 receptor vectors, 3 linker vectors, and 1 final expression vector. Different levels of vectors were used to complete seamless ligation between multiple DNA fragments by combining recognition sites and cleavage sites using type IIS restriction endonucleases and screening markers.

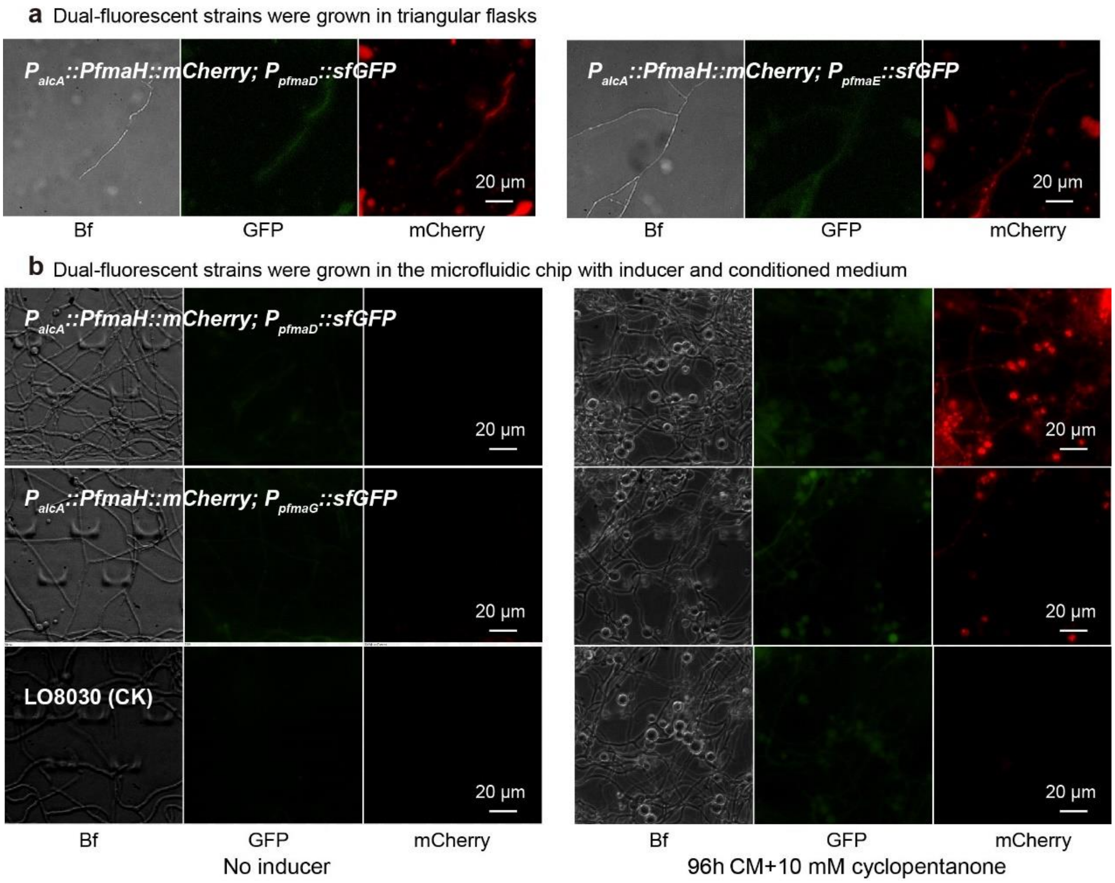

Figure S3. Culture conditions of dual-fluorescent strain on microfluidic chip. (a) Red fluorescence and green fluorescence were detected in the triangle flask. (b) Red fluorescence and green fluorescence were detected by adding inducer and conditioned media (CM) to microfluidic medium.

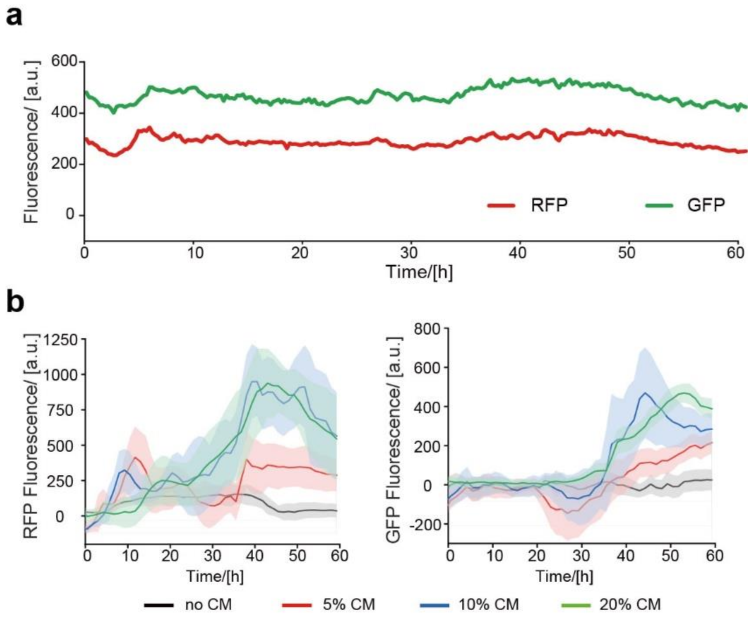

Figure S4. Preliminary study on culture conditions used in microfluidic system. (a) Background fluorescence production of *Aspergillus nidulans*. The sample sizes are 10. (b) Determination of the optimal amount of conditional media addition. The black, red, green, and blue lines represent the expression of PfmaH and P_{PfmaE} when different volumes of conditioned media were added, and the addition amount of cyclopentanone was 0.2 mM. Shading represents the range of population fluorescence and the solid line represents the average of multiple cell fluorescence data. The sample sizes on the left diagram are 12 (no), 9 (5%), 8 (10%), and 37 (20%). The sample sizes on the right diagram are 10 (no), 8 (5%), 13 (10%), and 12 (20%).

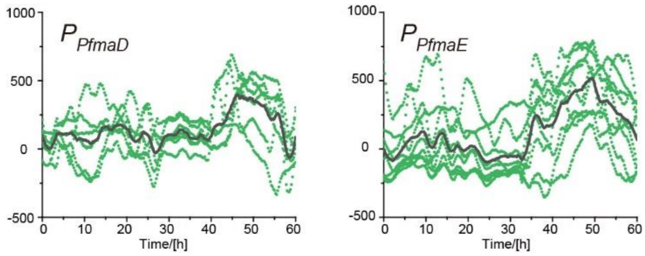

Figure S5. Fluorescence was monitored for 60 h in strains in which the transcription factor pfmaH was not fused to mCherry and the promoter manipulated GFP. The green section represents the green fluorescence data output, which represents the expression level of the promoter fused with sfGFP. The sample sizes are 8 (P_{pfmaD}), and 9 (P_{pfmaE}).

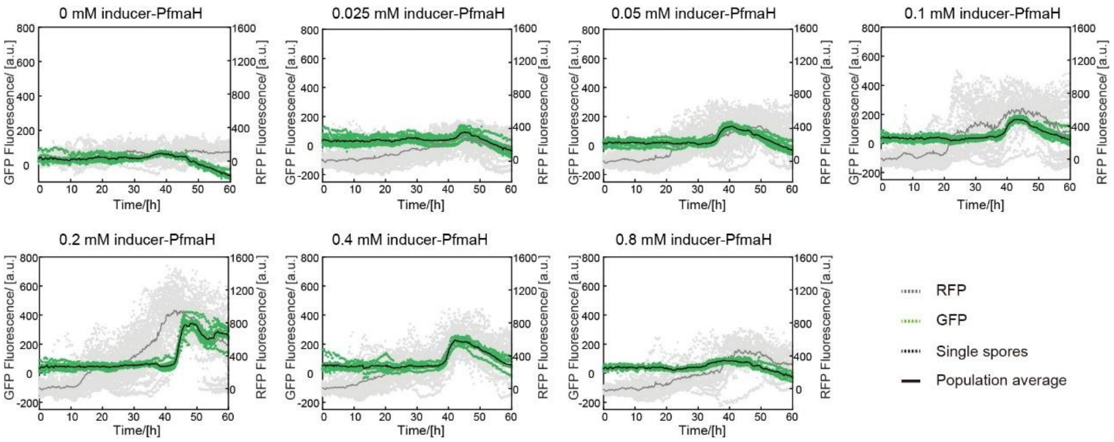

Figure S6. Effect of different concentrations of cyclopentanone on the expression of PfmaH-P_{pfmaD} pairs. The gray and green lines indicate the expression of PfmaH and P_{PfmaD} when seven different concentrations of cyclopentanone were added. Sample sizes for 0 mM treatment were 22 (PfmaH) and 8 (P_{pfmaD}), 39 (PfmaH) and 12 (P_{pfmaD}) for 0.025 mM treatment, 49 (PfmaH) and 11 (P_{pfmaD}) for 0.05 mM treatment, 30 (PfmaH) and 10 (P_{pfmaD}) for 0.1 mM treatment, 44 (PfmaH) and 11 (P_{pfmaD}) for 0.2 mM treatment, 30 (PfmaH) and 8 (P_{pfmaD}) for 0.4 mM treatment, and 20 (PfmaH) and 8 (P_{pfmaD}) for 0.8 mM treatment, respectively. The dashed line indicates the fluorescence signal of individual cells, and the solid line is the average of the fluorescence signal of the population.

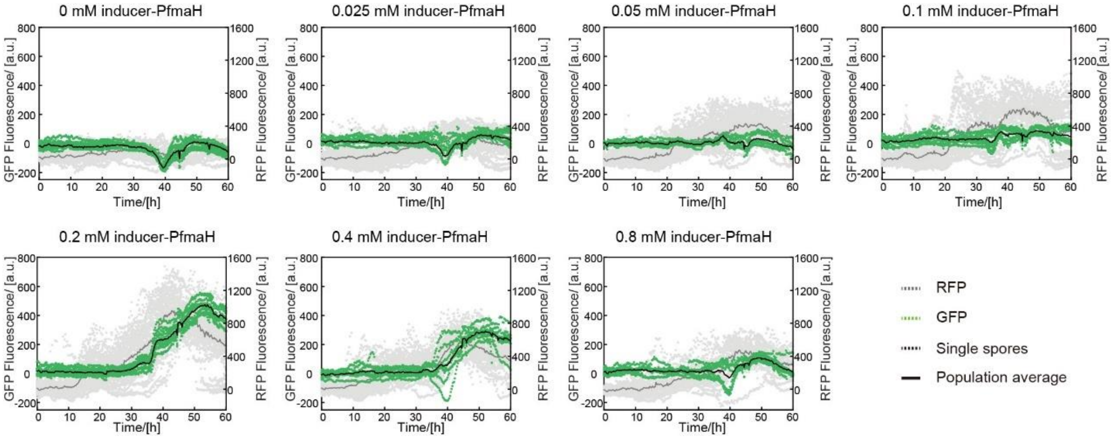

Figure S7. Effect of different concentrations of cyclopentanone on the expression of PfmaH-P_{pfmaE} pairs. The gray and green lines indicate the expression of PfmaH and P_{PfmaE} when seven different concentrations of cyclopentanone were added. Sample sizes for 0 mM treatment were 22 (PfmaH) and 13 (P_{pfmaE}), 39 (PfmaH) and 9 (P_{pfmaE}) for 0.025 mM treatment, 49 (PfmaH) and 8 (P_{pfmaE}) for 0.05 mM treatment, 30 (PfmaH) and 10 (P_{pfmaE}) for 0.1 mM treatment, 44 (PfmaH) and 12 (P_{pfmaE}) for 0.2 mM treatment, 30 (PfmaH) and 8 (P_{pfmaE}) for 0.4 mM treatment, and 20 (PfmaH) and 9 (P_{pfmaE}) for 0.8 mM treatment, respectively. The dashed line indicates the fluorescence signal of individual cells, and the solid line is the average of the fluorescence signal of the population.

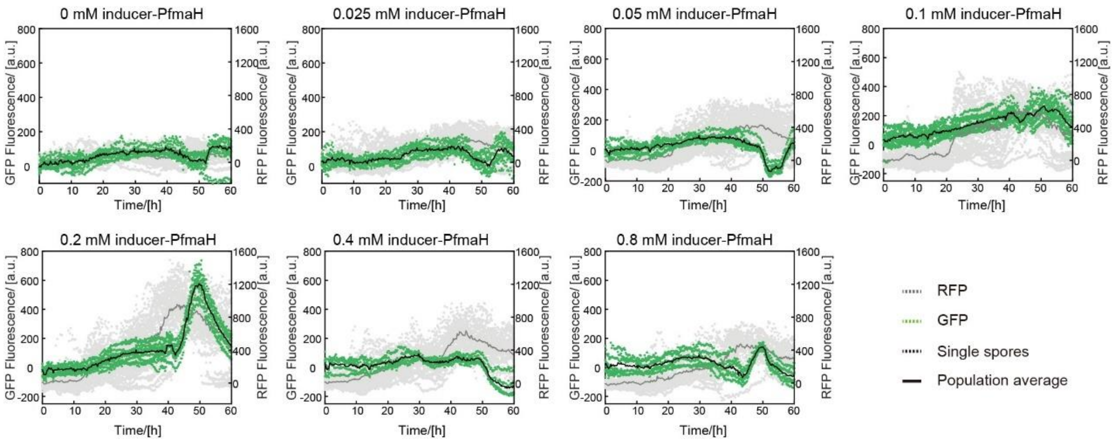

Figure S8. Effect of different concentrations of cyclopentanone on the expression of PfmaH-P_{pfmaG} pairs. The gray and green lines indicate the expression of PfmaH and P_{PfmaG} when seven different concentrations of cyclopentanone were added. Sample sizes for 0 mM treatment were 22 (PfmaH) and 8 (P_{pfmaG}), 39 (PfmaH) and 8 (P_{pfmaG}) for 0.025 mM treatment, 49 (PfmaH) and 8 (P_{pfmaG}) for 0.05 mM treatment, 30 (PfmaH) and 12 (P_{pfmaG}) for 0.1 mM treatment, 44 (PfmaH) and 10 (P_{pfmaG}) for 0.2 mM treatment, 30 (PfmaH) and 8 (P_{pfmaG}) for 0.4 mM treatment, and 20 (PfmaH) and 8 (P_{pfmaG}) for 0.8 mM treatment, respectively. The dashed line indicates the fluorescence signal of individual cells, and the solid line is the average of the fluorescence signal of the population.

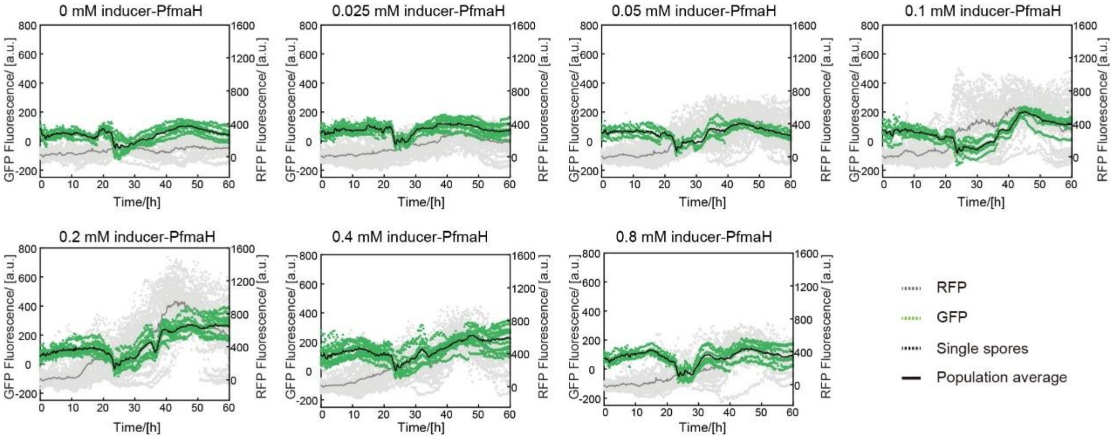

Figure S9. Effect of different concentrations of cyclopentanone on the expression of PfmaH-P_{pfmaJ} pairs. The gray and green lines indicate the expression of PfmaH and P_{PfmaJ} when seven different concentrations of cyclopentanone were added. Sample sizes for 0 mM treatment were 22 (PfmaH) and 8 (P_{pfmaJ}), 39 (PfmaH) and 11 (P_{pfmaJ}) for 0.025 mM treatment, 49 (PfmaH) and 8 (P_{pfmaJ}) for 0.05 mM treatment, 30 (PfmaH) and 8 (P_{pfmaJ}) for 0.1 mM treatment, 44 (PfmaH) and 11 (P_{pfmaJ}) for 0.2 mM treatment, 30 (PfmaH) and 12 (P_{pfmaJ}) for 0.4 mM treatment, and 20 (PfmaH) and 8 (P_{pfmaJ}) for 0.8 mM treatment, respectively. The dashed line indicates the fluorescence signal of individual cells, and the solid line is the average of the fluorescence signal of the population.

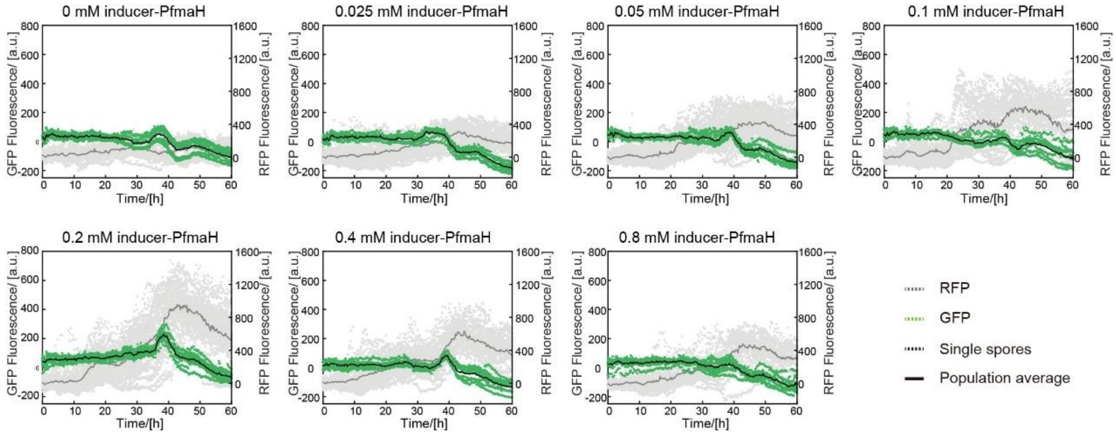

Figure S10. Effect of different concentrations of cyclopentanone on the expression of PfmaH-P_{pfmaI} pairs. The gray and green lines indicate the expression of PfmaH and P_{PfmaI} when seven different concentrations of cyclopentanone were added. Sample sizes for 0 mM treatment were 22 (PfmaH) and 9 (P_{pfmaI}), 39 (PfmaH) and 9 (P_{pfmaI}) for 0.025 mM treatment, 49 (PfmaH) and 10 (P_{pfmaI}) for 0.05 mM treatment, 30 (PfmaH) and 8 (P_{pfmaI}) for 0.1 mM treatment, 44 (PfmaH) and 11 (P_{pfmaI}) for 0.2 mM treatment, 30 (PfmaH) and 8 (P_{pfmaI}) for 0.4 mM treatment, and 20 (PfmaH) and 9 (P_{pfmaI}) for 0.8 mM treatment, respectively. The dashed line indicates the fluorescence signal of individual cells, and the solid line is the average of the fluorescence signal of the population.

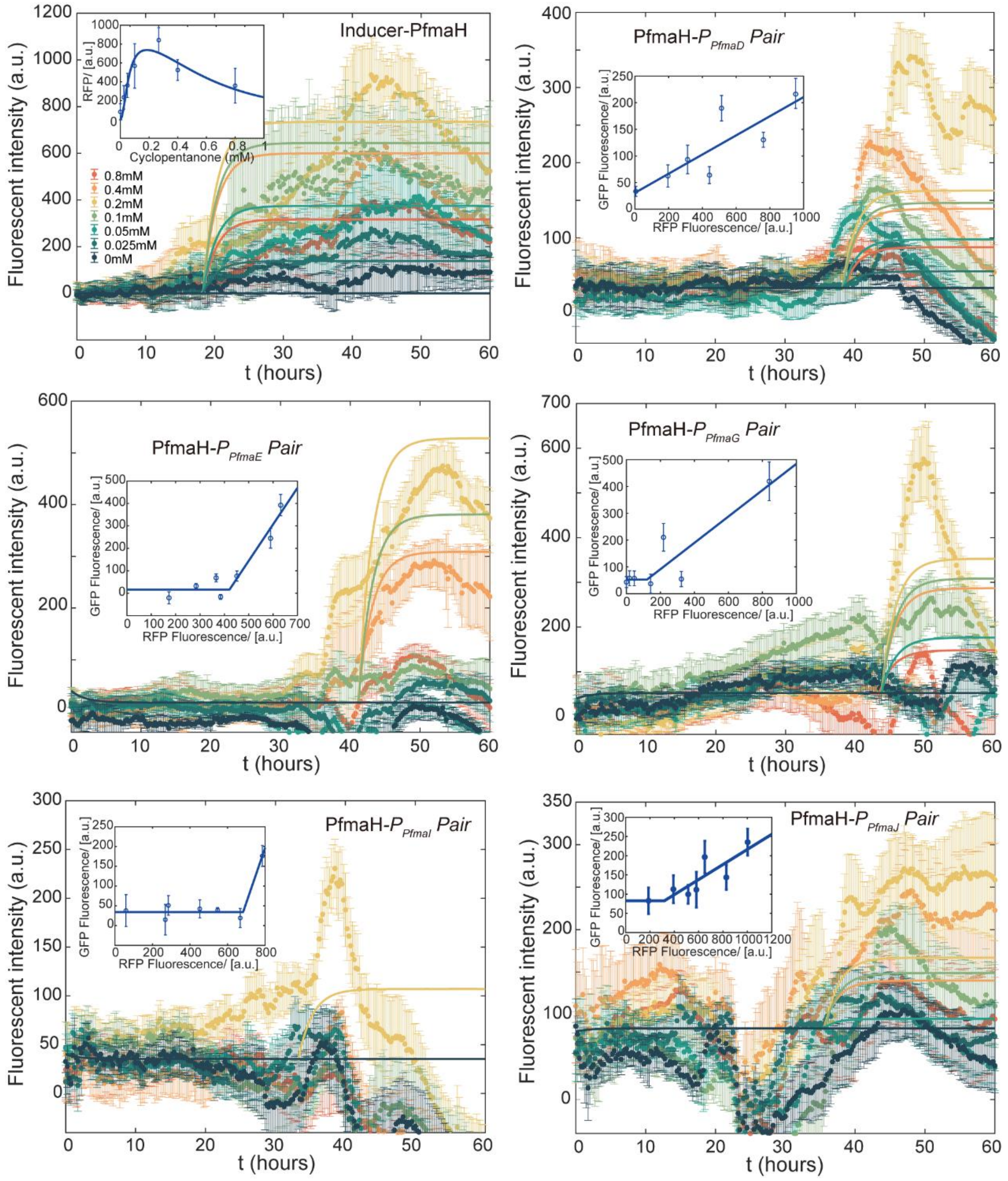

Figure S11. Fitting to determine the time needed for the transcription factor PfmaH or the downstream promoters to start express after induction. Different colors mean different cyclopentanone concentrations.

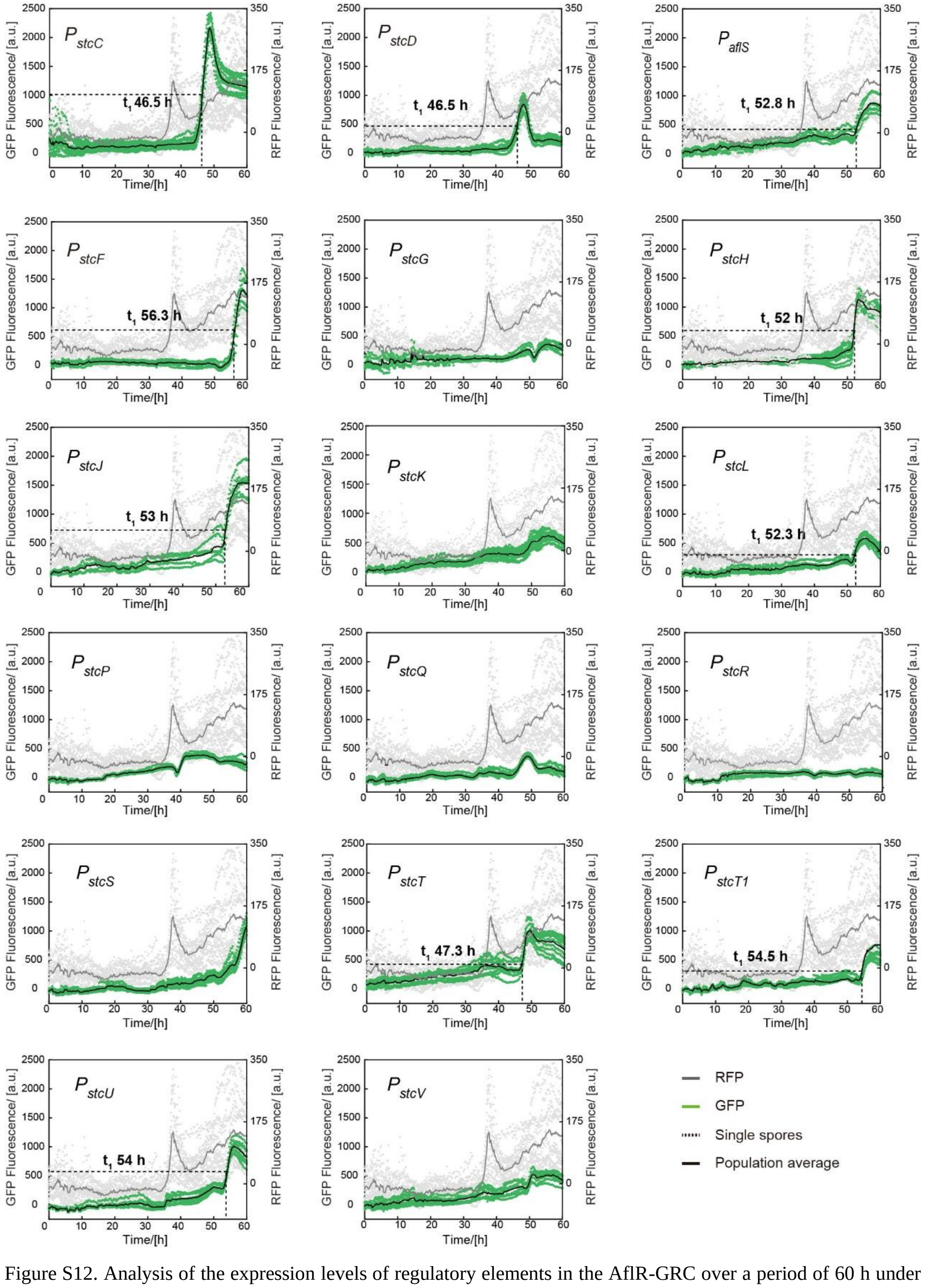

Figure S12. Analysis of the expression levels of regulatory elements in the AflR-GRC over a period of 60 h under optimal culture conditions. This figure displays data for 17 of the promoters, with the remaining 8 shown in Figure 4b. Each dashed line represents the fluorescence data of a single cell, while the solid line indicates the average of multiple single cell fluorescence data. The gray area displays the red fluorescence data output representing AflR expression level, with values reflected on the right y-axis, the sample sizes for all graphs are 14. The green area displays the green fluorescence data output indicating the promoter expression level fused with sfGFP. The sample sizes are 14 (P_{stcC}), 11 (P_{stcD}), 8 (P_{aflS}), 8 (P_{stcF}), 8 (P_{stcG}), 19 (P_{stcH}), 8 (P_{stcJ}) , 13 (P_{stcK}) , 9 (P_{stcL}) , 8 (P_{stcP}) , 8 (P_{stcQ}) ,

8 (P_{stcR}) , 12 (P_{stcS}) , 10 (P_{stcT}) , 9 (P_{stcT1}) , 10 (P_{stcU}) and 9 (P_{stcV}) respectively. t_1 represents the time point at which the promoter expression reaches half of the plateau under optimal conditions. For some promoters, t_1 time point was not labeled since they did not show significant expression increase at 60 h.

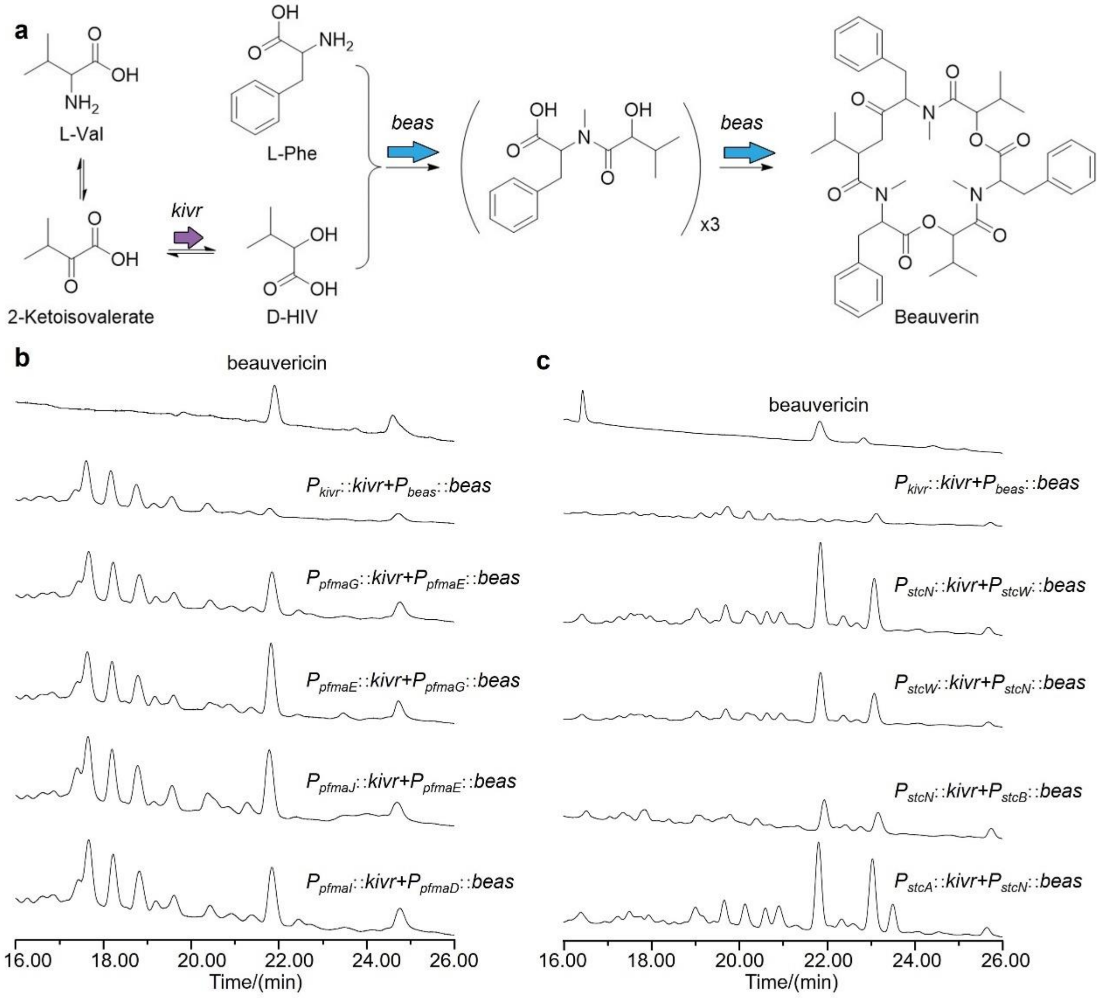

Figure S13. Biosynthetic pathway of beauvericin [68] (a). HPLC metabolic profiles of culture extracts of the recombinant biosynthetic pathway of beauvericin in PfmaH-GRC (b) and AflR-GRC (c). UV absorptions at 210 nm are illustrated.

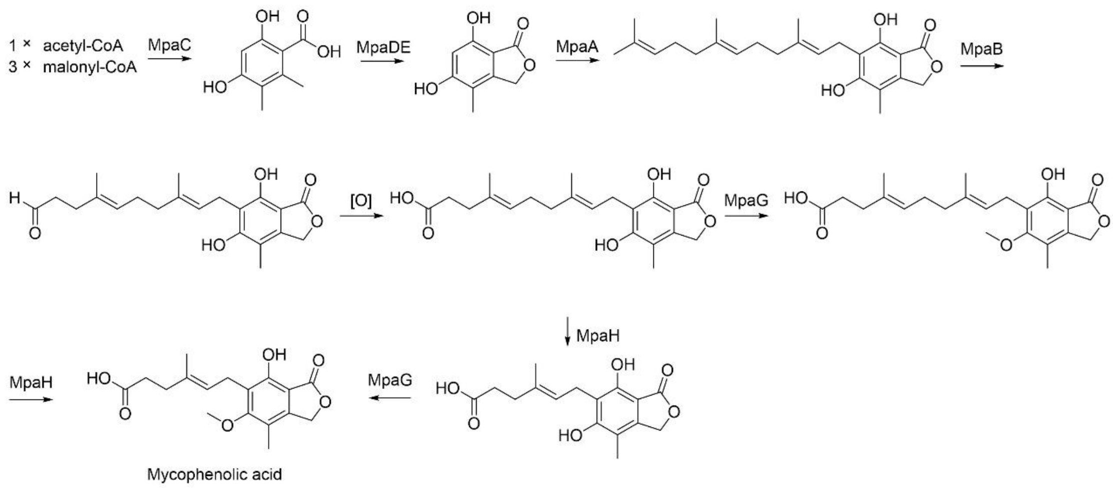

Figure S14. Biosynthetic pathway of mycophenolic acid[69].

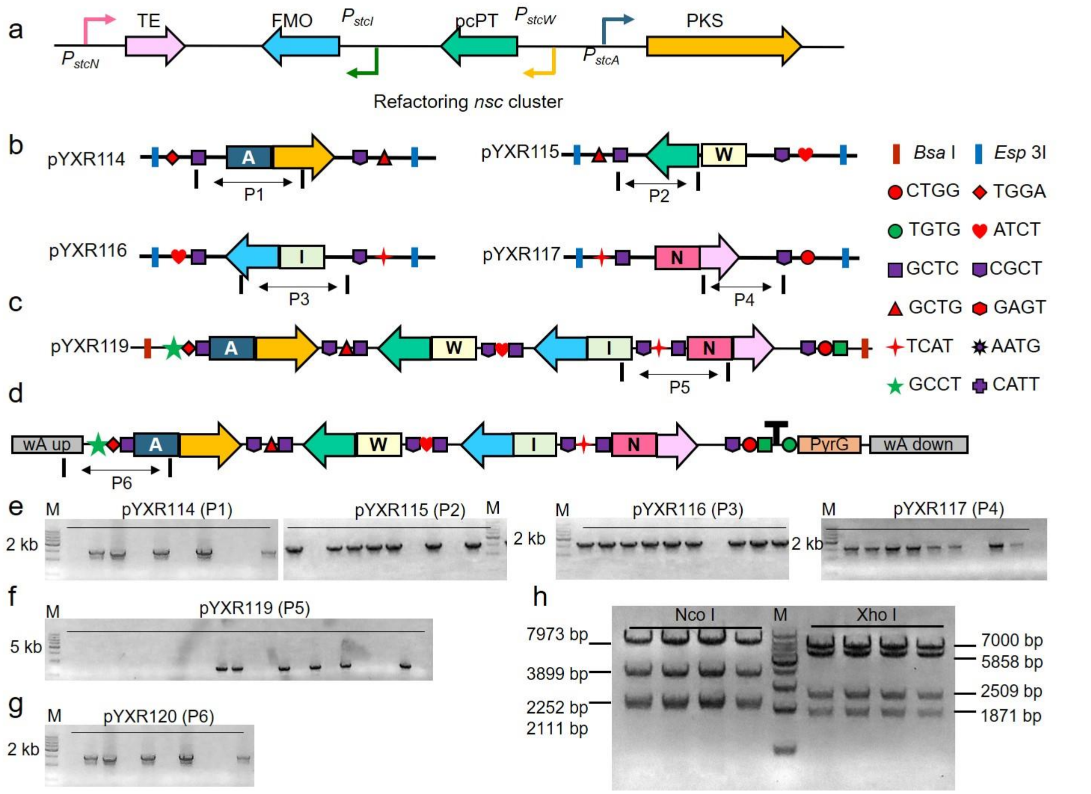

Figure S15. Construction of the expression vector of *nsc* refactoring cluster. (a) Schematic diagram of the *nsc* refactoring gene cluster. (b) Construction of level 1 vector. The four functional genes in the *nsc* gene cluster were combined with P_{stcA}, P_{stcN}, P_{stcI}, and P_{stcW} in AflR-GRC according to gene orientation to obtain the level 1 vectors pYXR114, pYXR115, pYXR116 and pYXR117. (c) Construction of level 2 vector. Four level 1 vectors were linked end-to-end by type IIS restriction endonuclease Esp 3I and T4 ligase to form level 2 vector pYXR119. (d) Construction of *Aspergillus nidulans* heterologous expression vector with refactoring *nsc* cluster. Level 2 vector and ligating vector were ligated to level 2i recipient vector in the presence of type IIS restriction endonuclease and T4 ligase. (e-g) *E. coli* colony PCR to verify the correctness of multilevel vectors. (h) Validation of plasmids (pYXR120 *nsc* refactoring cluster) by enzymatic digestion. Restriction endonuclease Nco l was used with target band sizes of 7973, 3899, 2252, and 2111 bp, respectively. Restriction endonuclease Xho I was used with target band sizes of 7000, 5858, 2509, and 1871 bp, respectively.

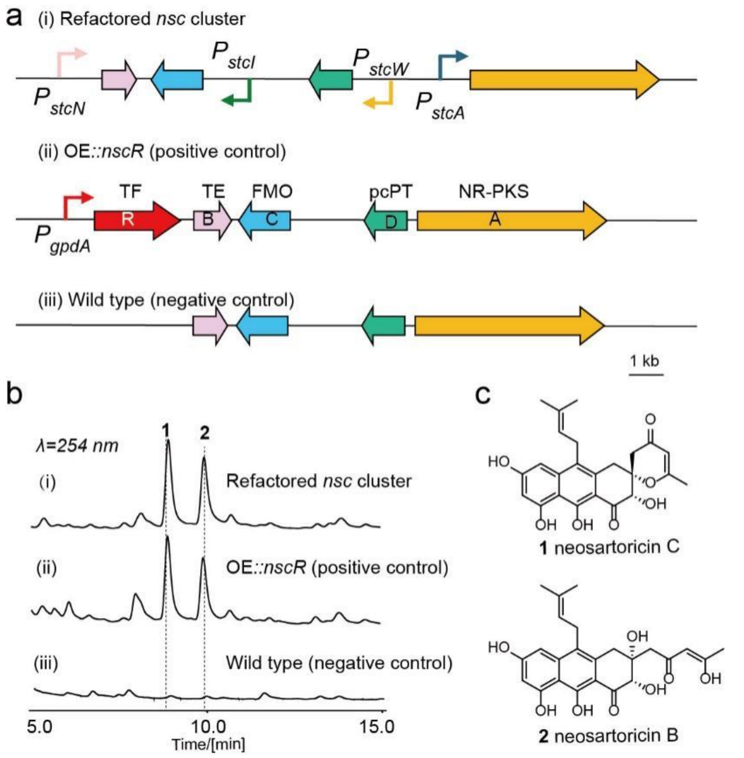

Figure S16. Refactoring the silent *nsc* pathway using AflR-controlled promoters from AflR-GRCs. (**a**) The neosartoricin biosynthetic pathway was refactored by constructing three strains: one with AflR-controlled promoters fused and recombined, one with PSTF overexpressed in the *nsc* gene cluster and synthetic genes, and one with the original *nsc* gene cluster without PSTF. (**b**) HPLC analysis (at 254 nm) of the reconstructed pathway for neosartoricin activation. (**c**) Identified compounds from the refactored biosynthetic pathway of *nsc*.

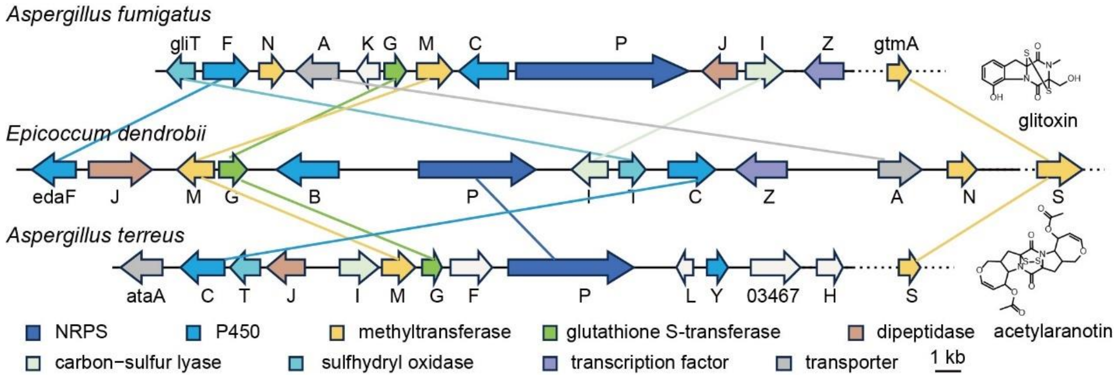

Figure S17. The *eda* gene cluster in *Epicoccum dendrobii* and its homologous gene cluster which synthesize gliotoxin and acetylaranotin, respectively.

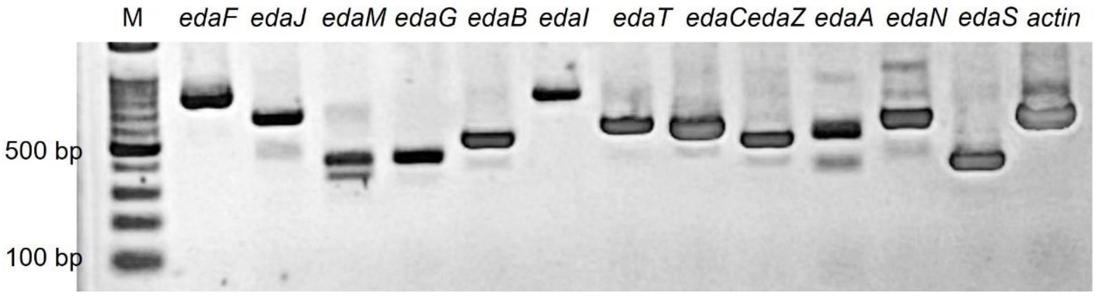

Figure S18. Reverse transcription polymerase chain reaction (RT-PCR) analysis of genes in *eda* cluster.

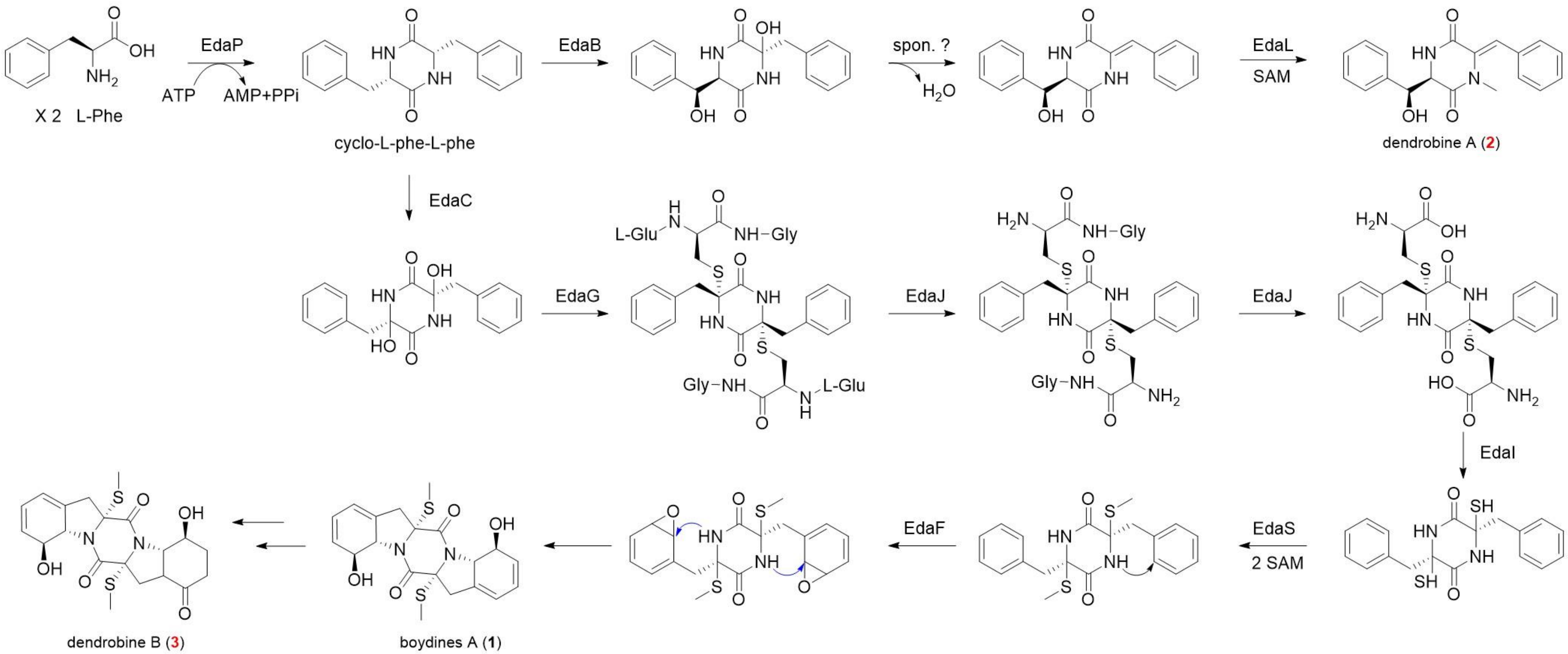

Figure S19. Proposed biosynthetic pathway of ETP derivatives. New compounds **2** and **3** are highlighted in red

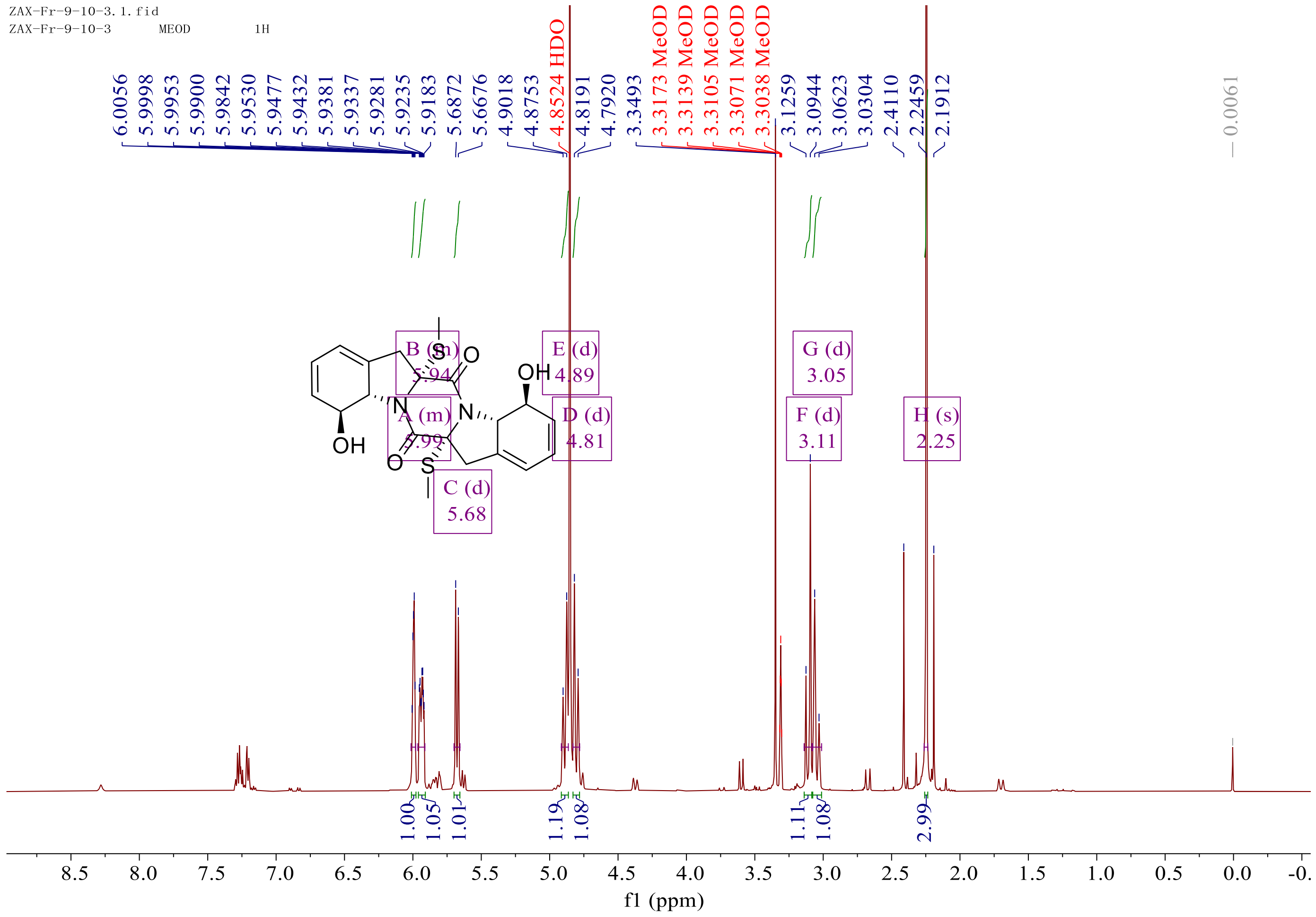

Figure S20. ^{1}H-NMR spectrum (500 MHz) of boydines A (**1**).

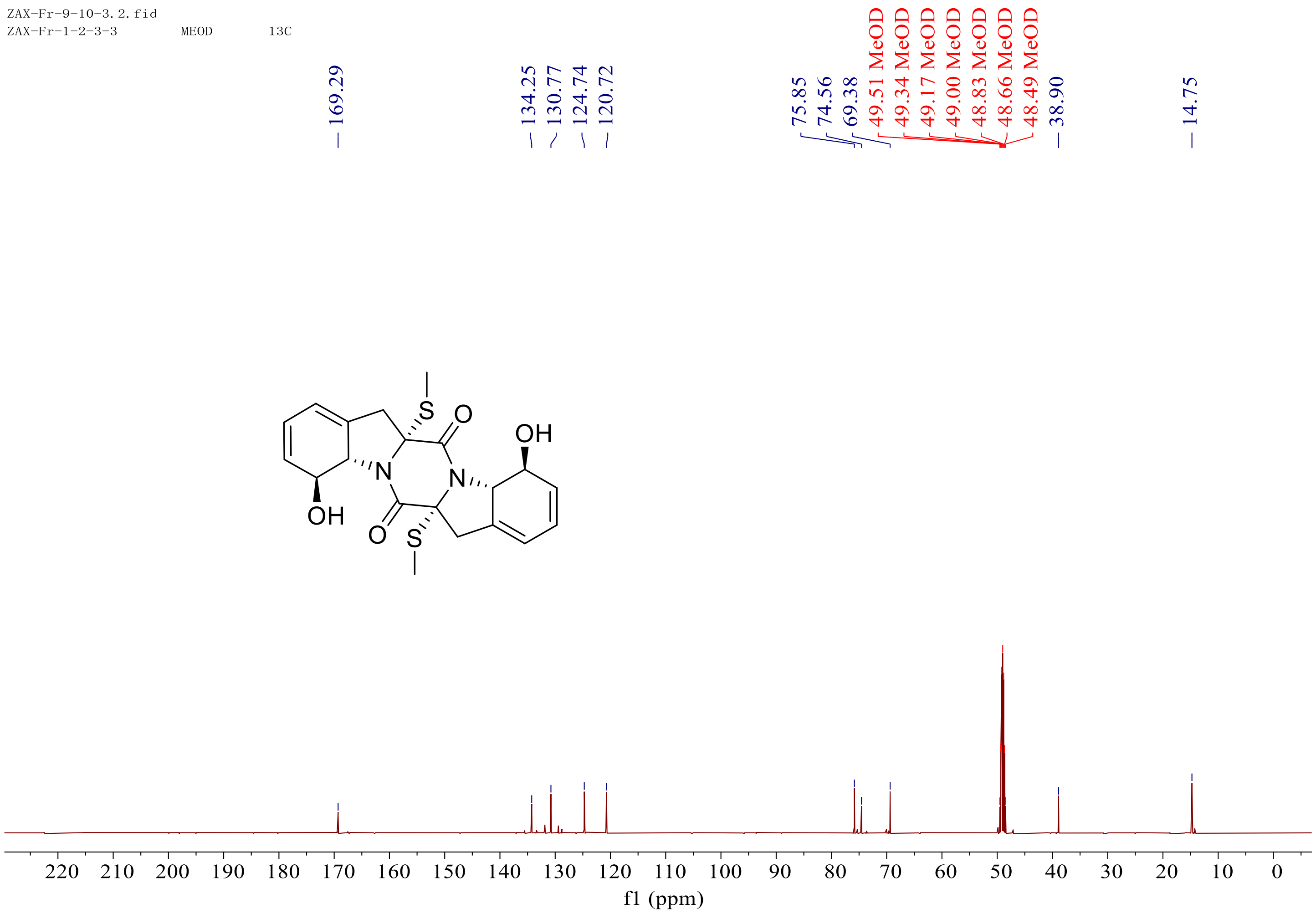

Figure S21. ^{13}C-NMR spectrum (125 MHz) of boydines A (**1**).

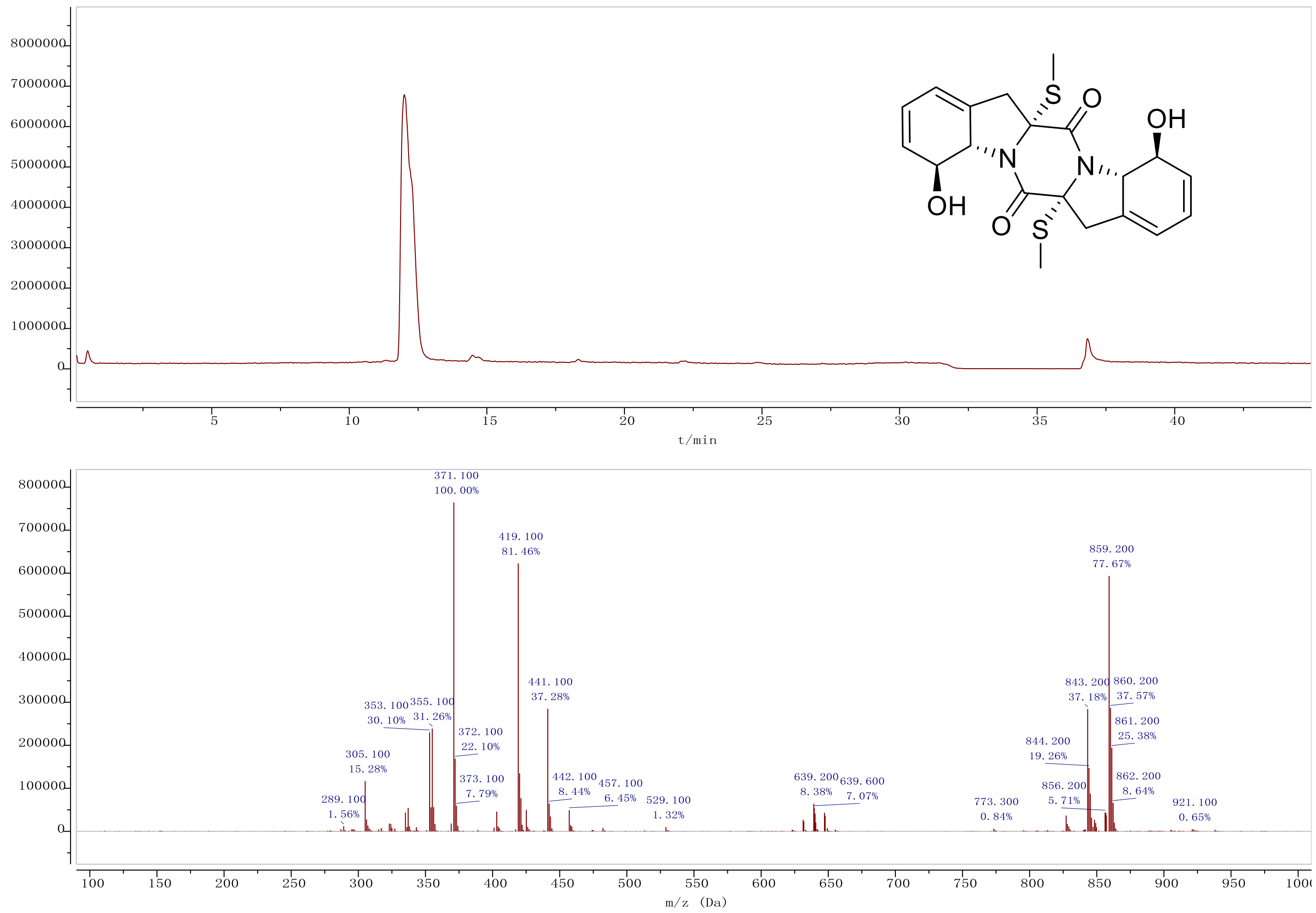

Figure S22. LC-MS spectrum of boydines A (**1**).

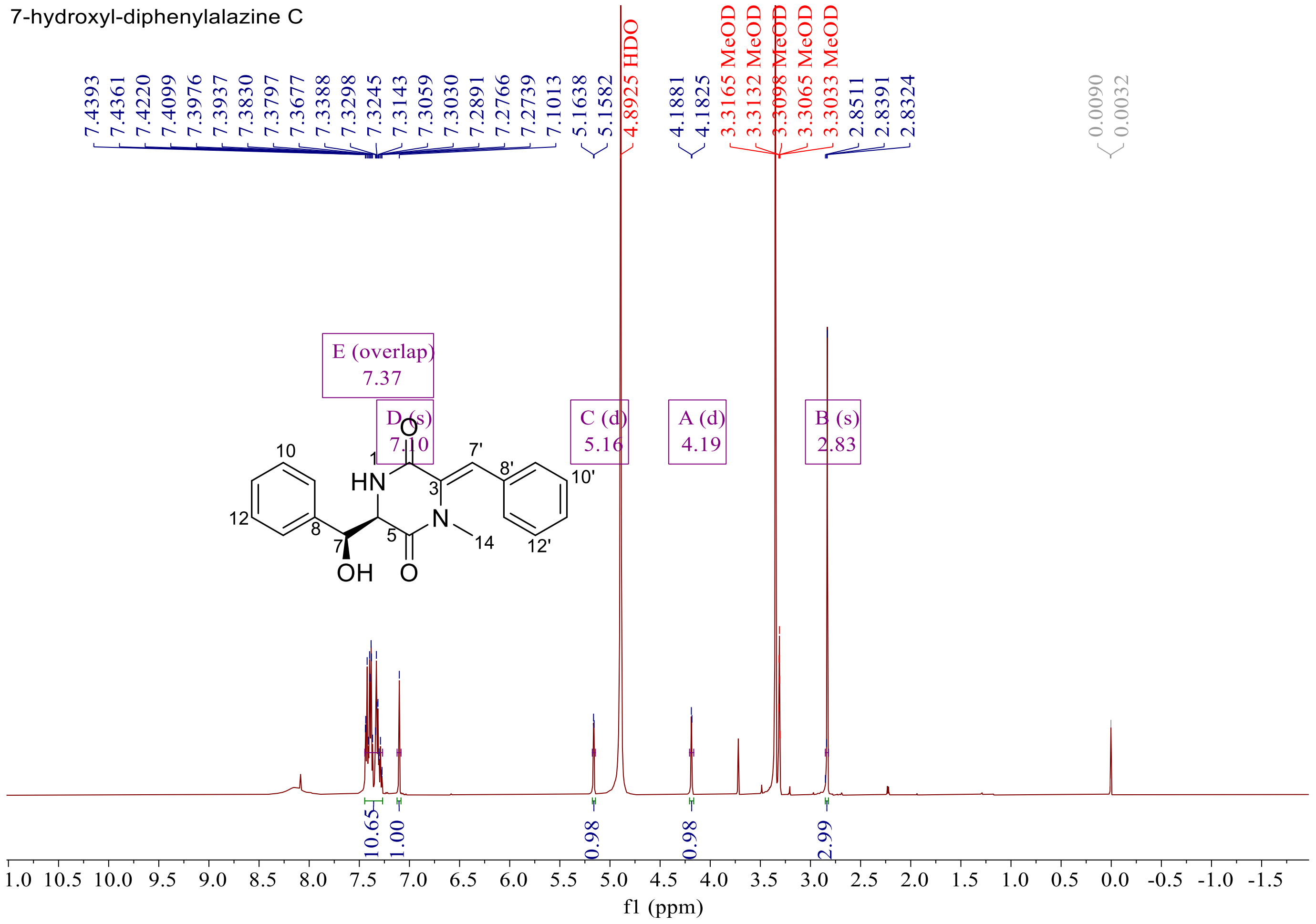

Figure S23. ^{1}H-NMR spectrum (500 MHz) of dendrobine A (**2**).

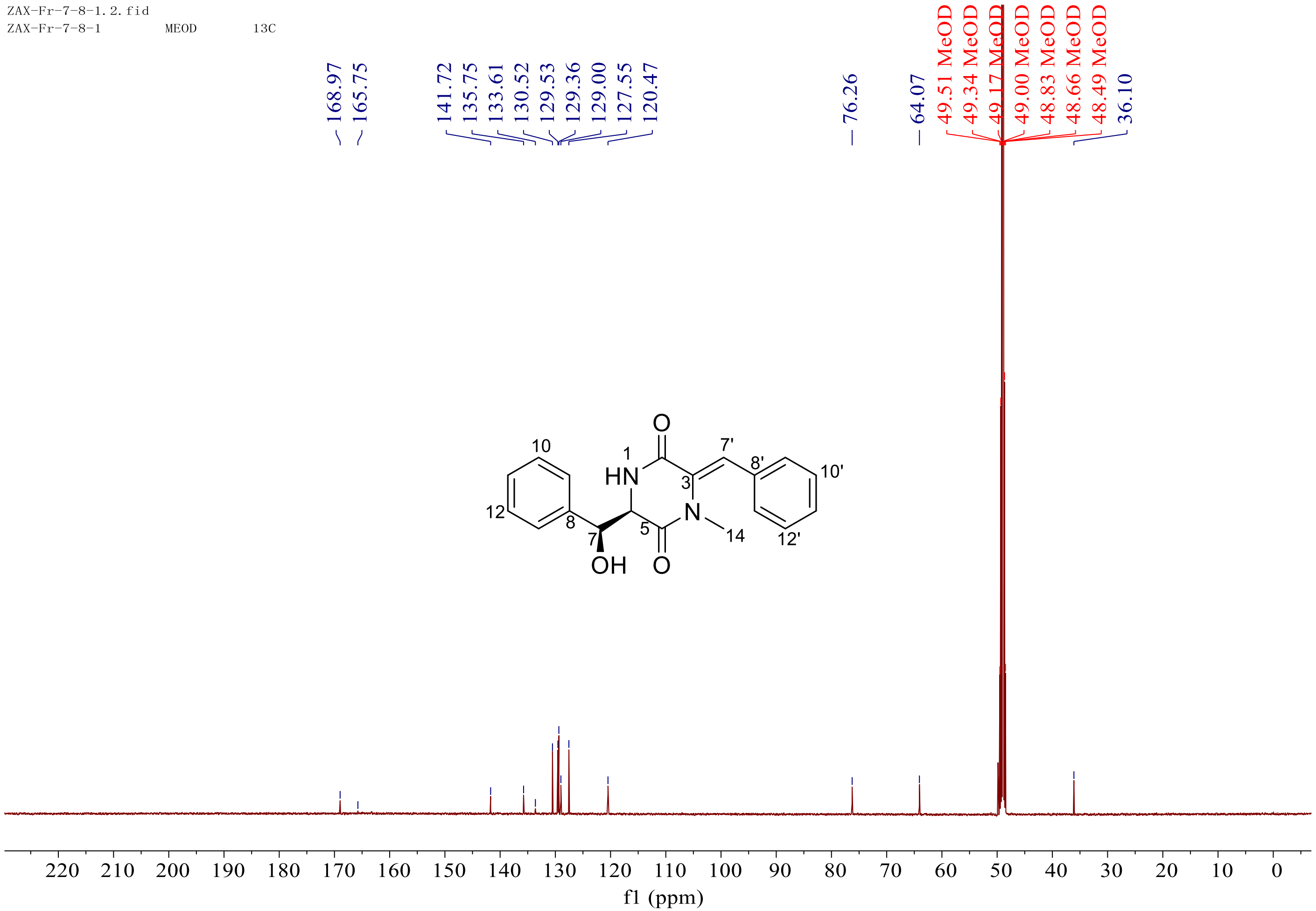

Figure S24. ^{13}C NMR spectrum (125 MHz) of dendrobine A (**2**).

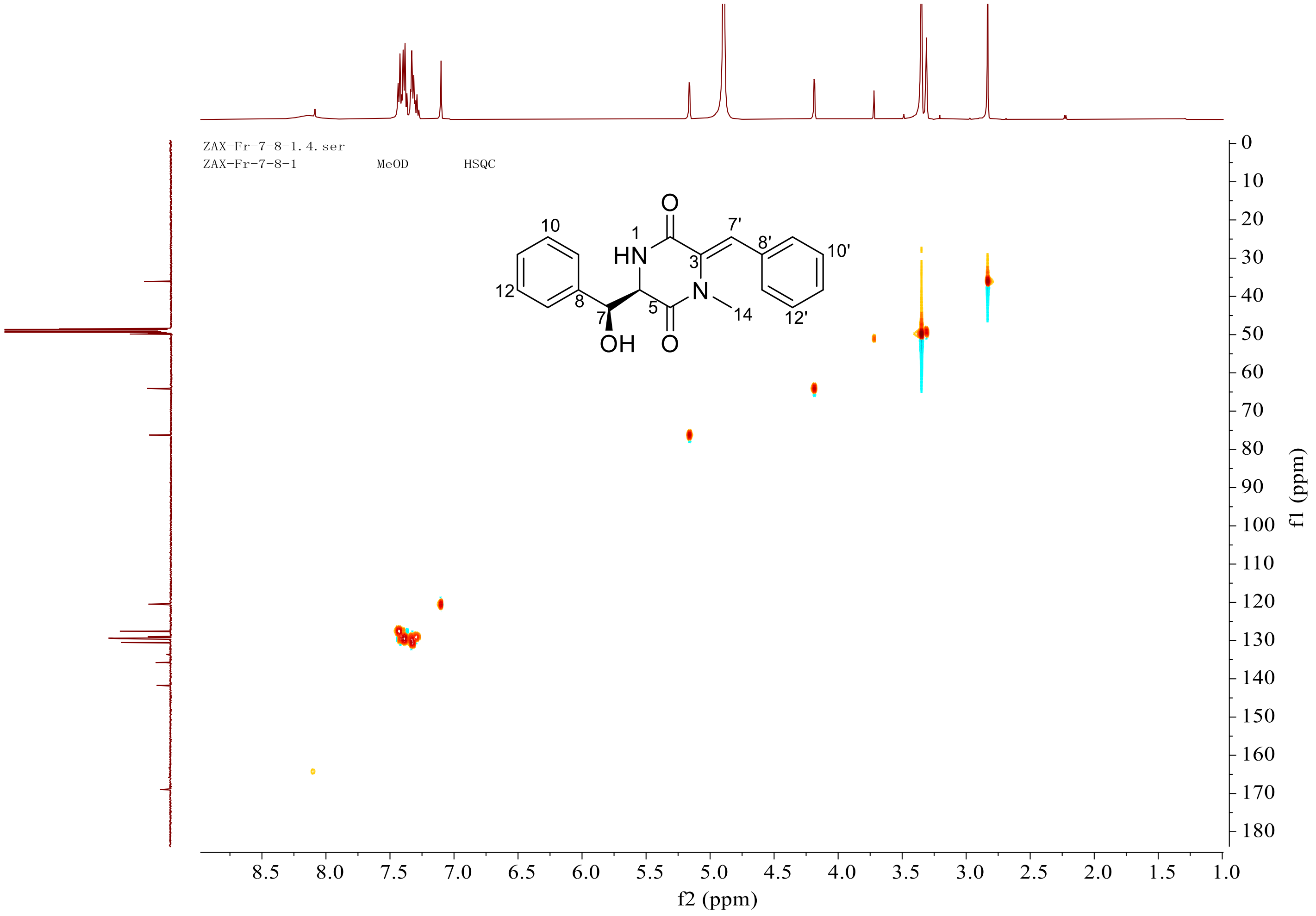

Figure S25. HSQC spectrum of dendrobine A (**2**)

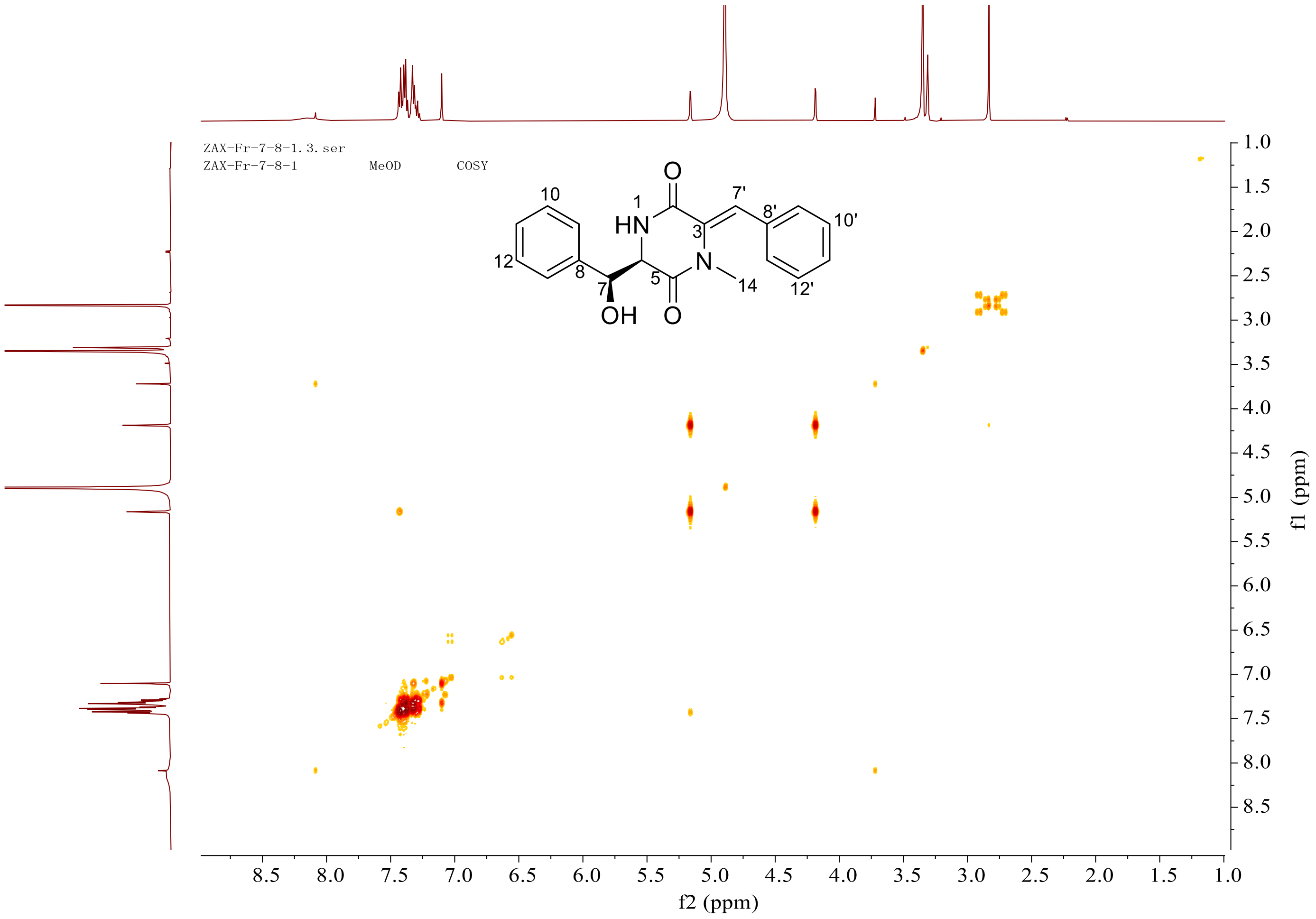

Figure S26. COSY spectrum of dendrobine A (**2**).

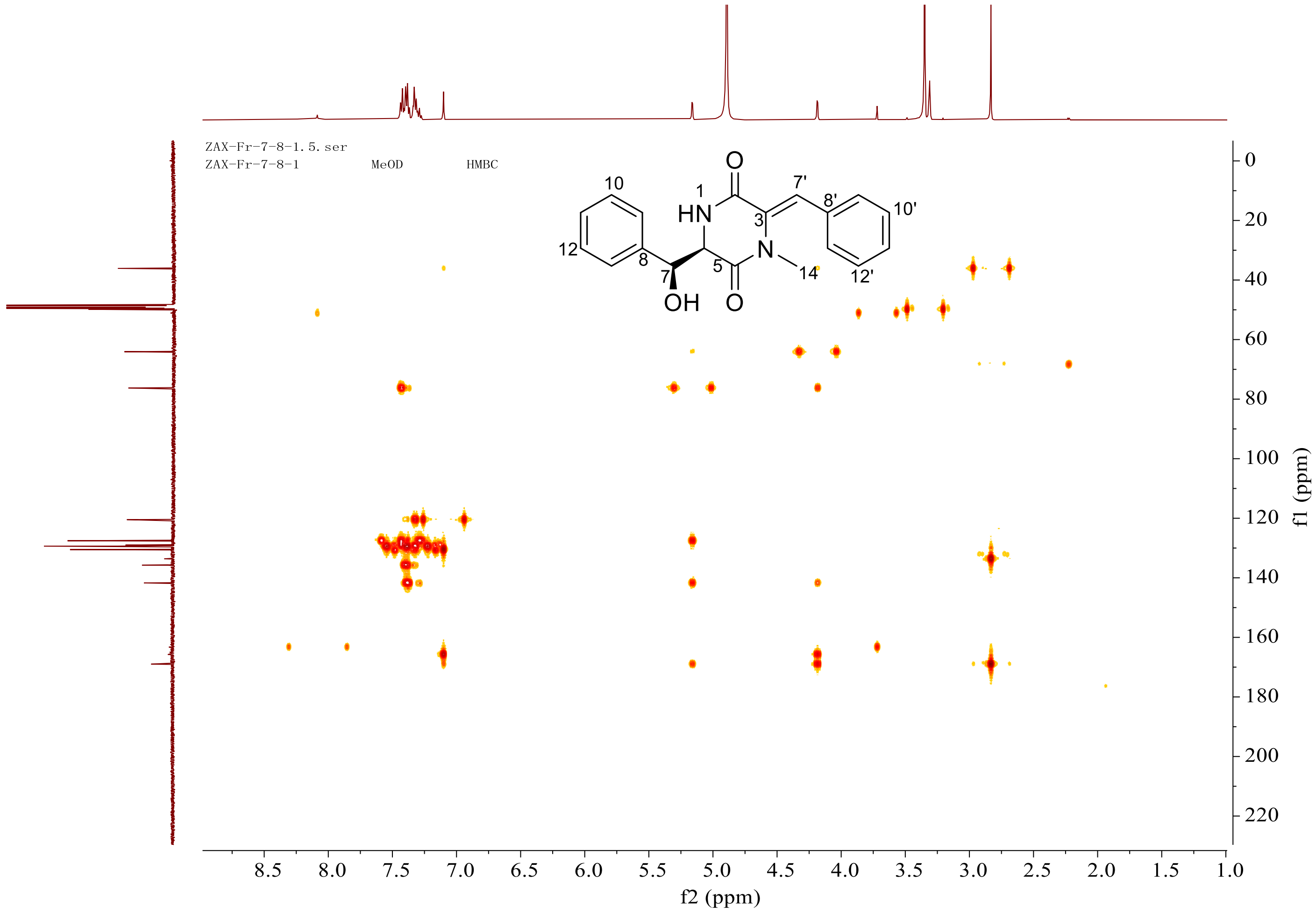

Figure S27. HMBC spectrum of dendrobine A (**2**).

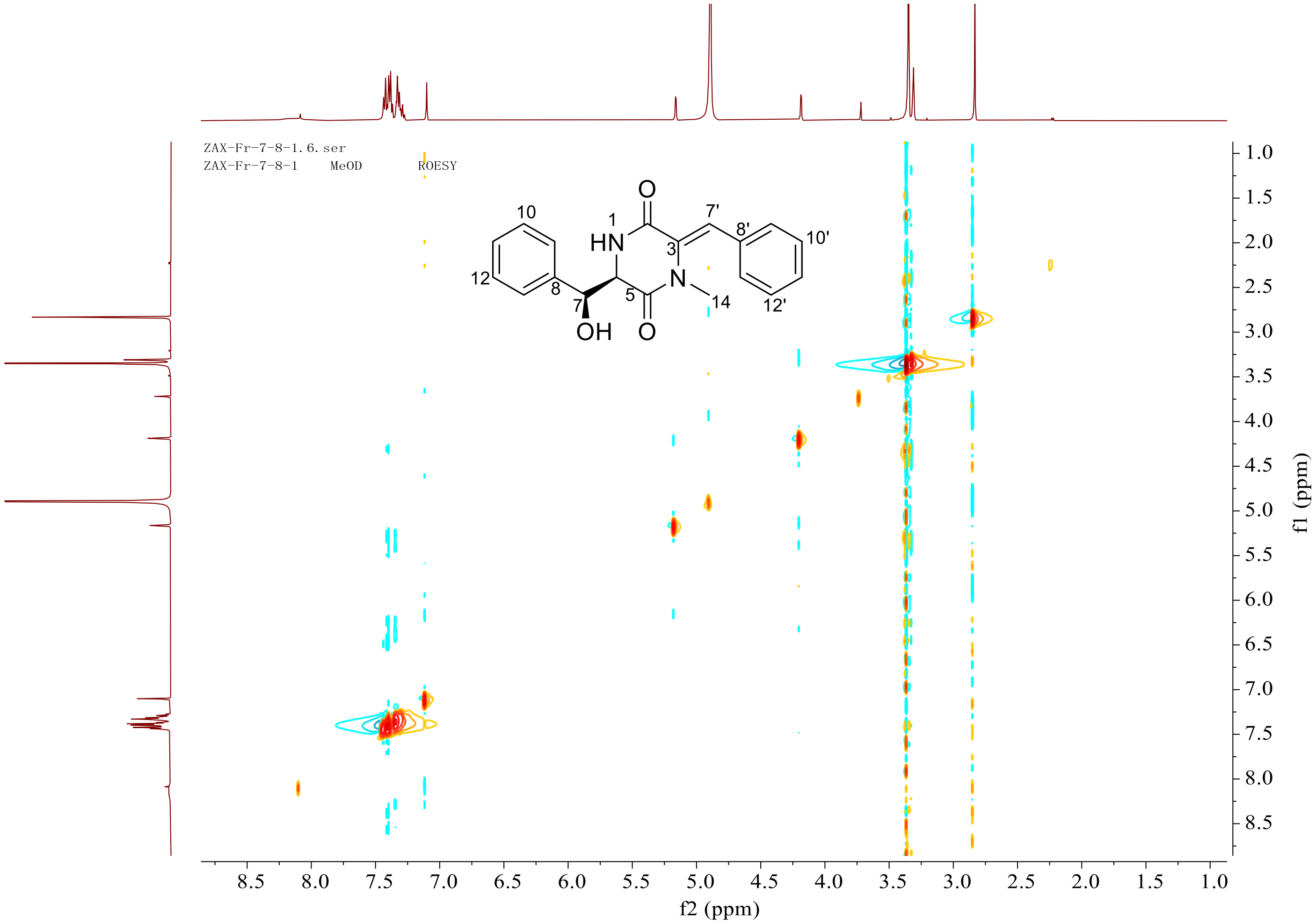

Figure S28. ROESY spectrum of dendrobine A (**2**).

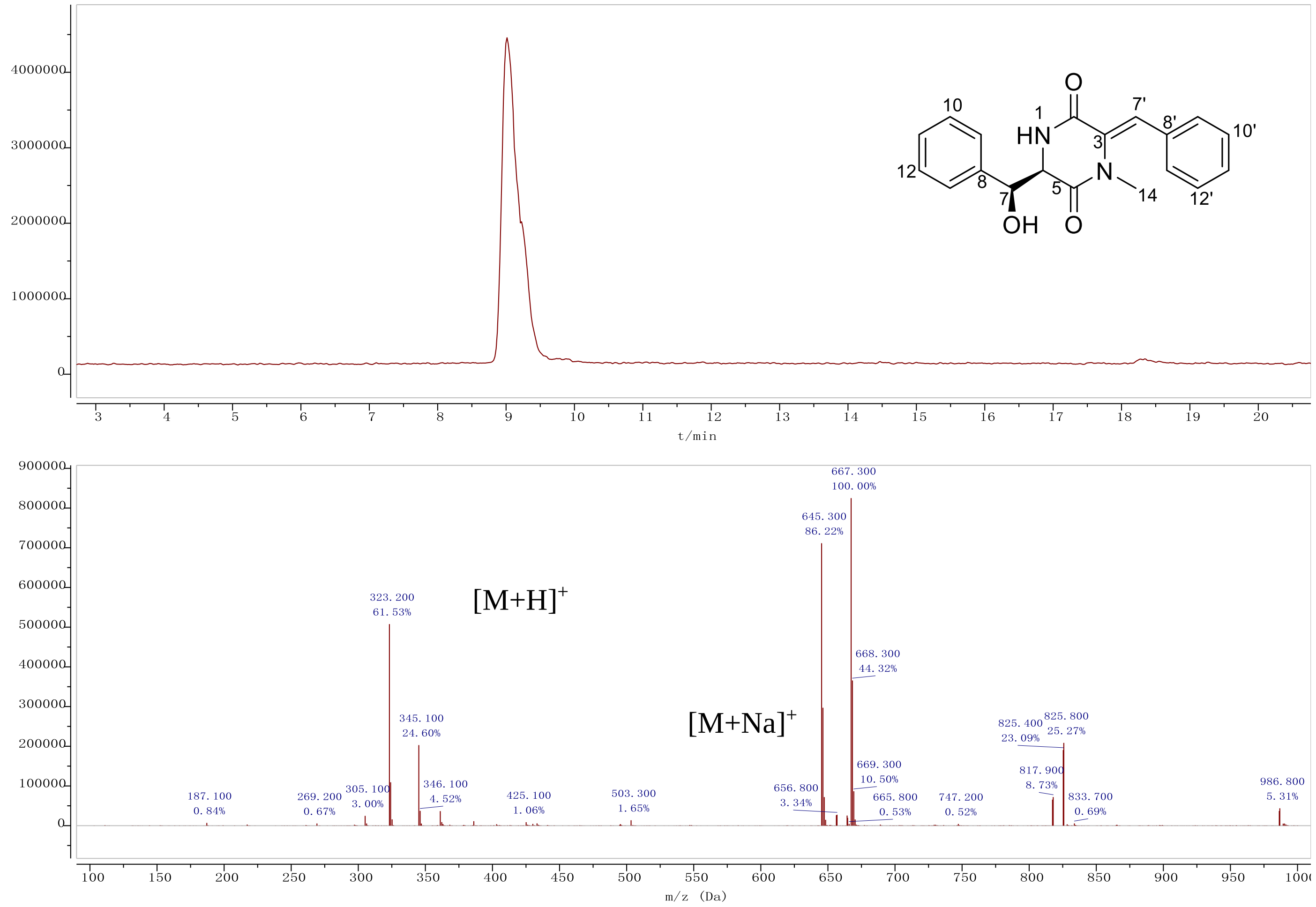

Figure S29. LC-MS spectrum of dendrobine A (**2**).

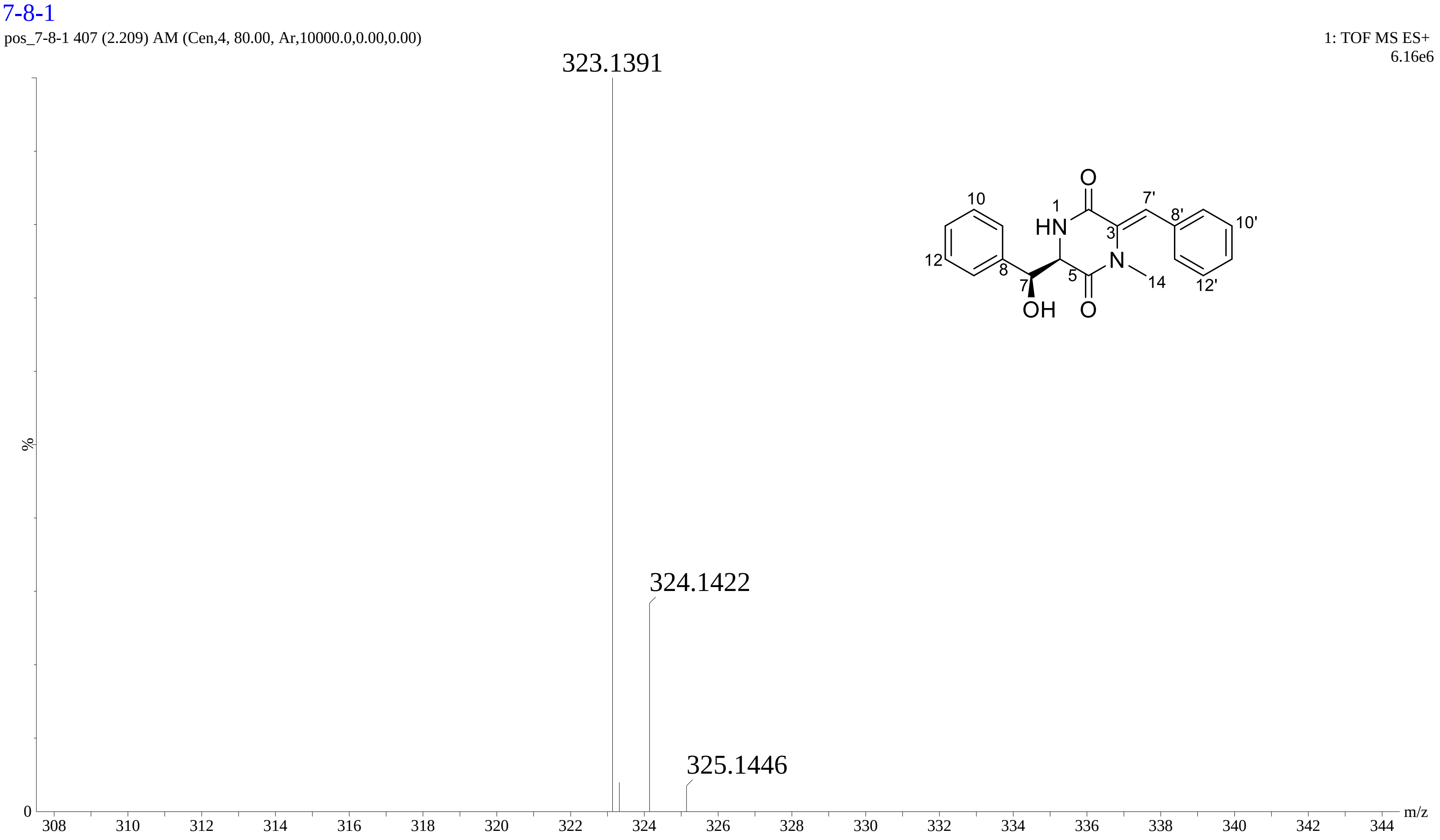

Figure S30. HRMS spectrum of dendrobine A (**2**).

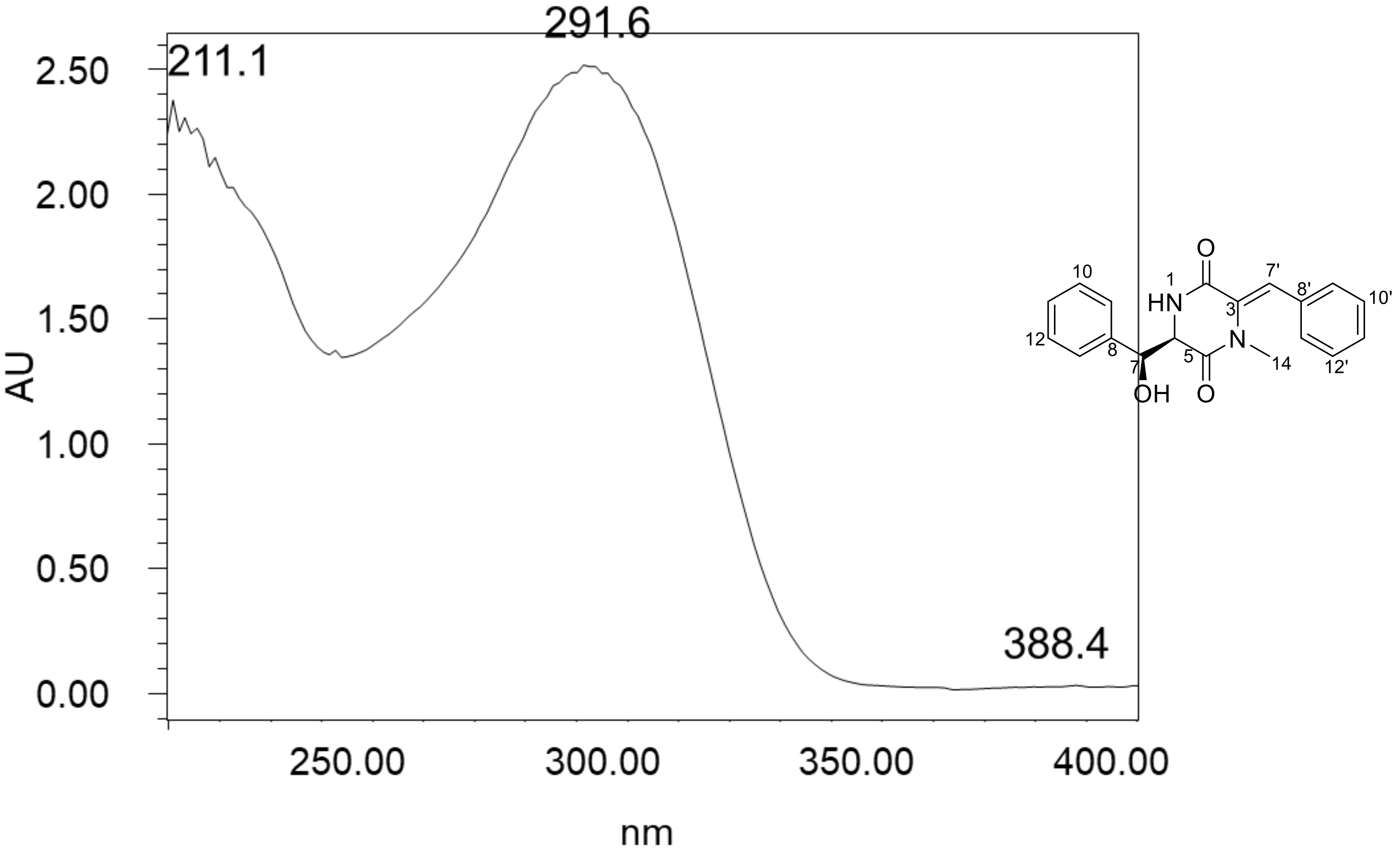

Figure S31. UV spectrum of dendrobine A (**2**) in CH_3OH.

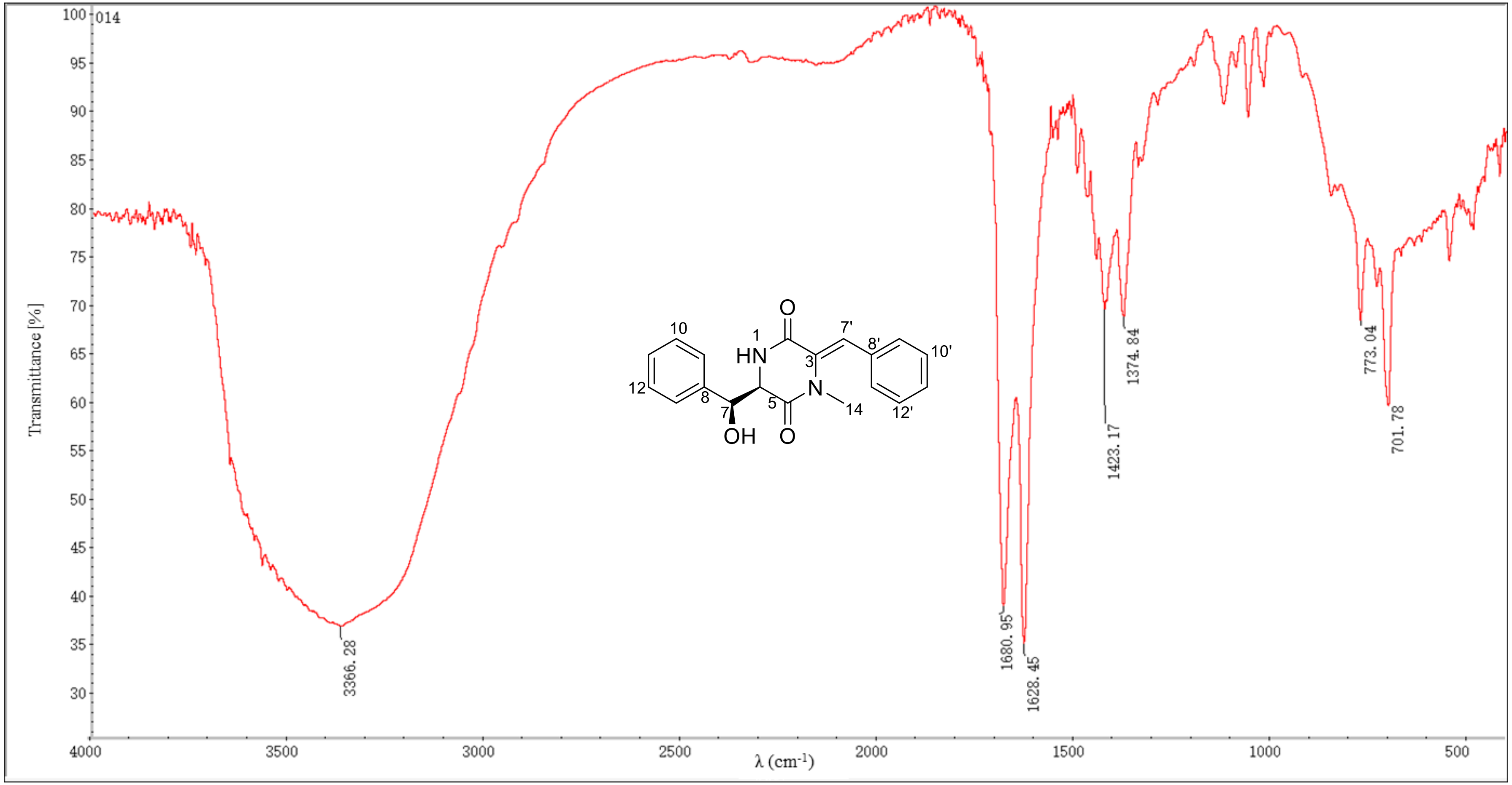

Figure S32. IR spectrum of dendrobine A (**2**).

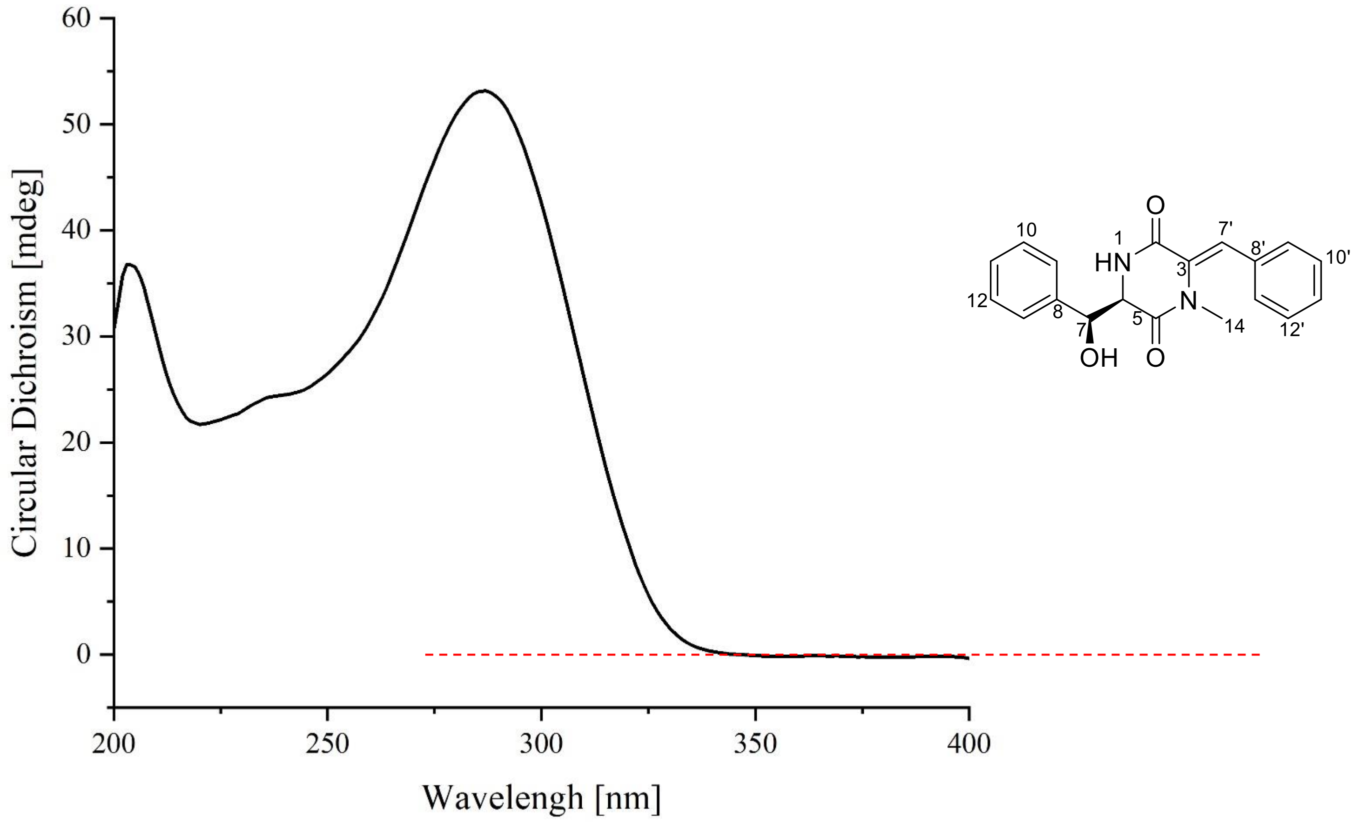

Figure S33. CD spectrum of dendrobine A (**2**) in CH_3OH.

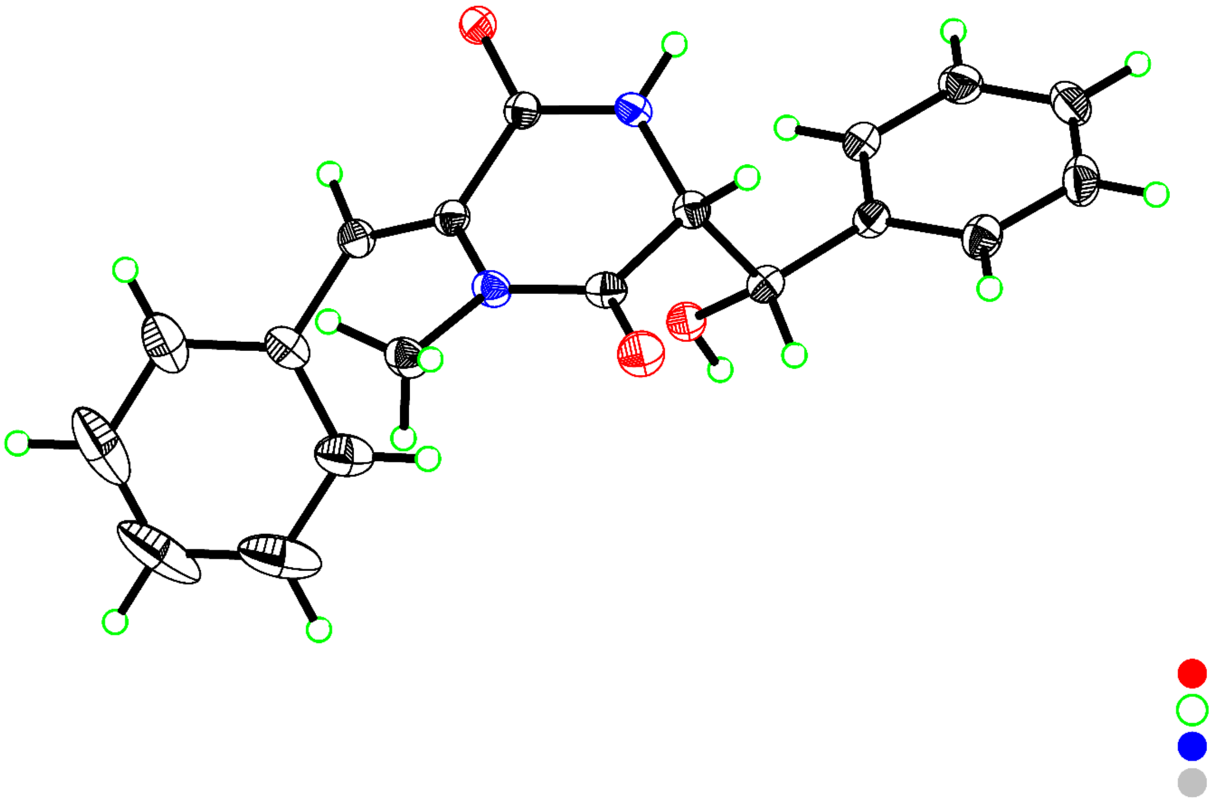

Figure S34. Thermal ellipsoid plot for dendrobine A (**2**) with full-page size.

checkCIF/PLATON report

Structure factors have been supplied for datablock(s) zax_7_8_1_auto

THIS REPORT IS FOR GUIDANCE ONLY. IF USED AS PART OF A REVIEW PROCEDURE FOR PUBLICATION, IT SHOULD NOT REPLACE THE EXPERTISE OF AN EXPERIENCED CRYSTALLOGRAPHIC REFEREE.

No syntax errors found. CIF dictionary Interpreting this report

Datablock: zax_7_8_1_auto

Bond precision: C-C = 0.0027 A Wavelength=1.54184

Cell:	a=9.1273(1)	b=18.7169(2)	c=20.1694(2)
	alpha=90	beta=90	gamma=90
Temperature:	100 K		

	Calculated	Reported
Volume	3445.64(6)	3445.64(6)
Space group	P 21 21 21	P 21 21 21
Hall group	P 2ac 2ab	P 2ac 2ab
Moiety formula	2(C19 H18 N2 O3), C H4 O	C19 H18 N2 O3, 0.5(C H4 O)
Sum formula	C39 H40 N4 O7	C19.50 H20 N2 O3.50
Mr	676.75	338.37
Dx,g cm-3	1.305	1.305
Z	4	8
Mu (mm-1)	0.737	0.737
F000	1432.0	1432.0
F000'	1436.47	
h,k,lmax	11,23,25	11,23,25
Nref	7297[4092]	7030
Tmin,Tmax	0.923,0.978	0.873,1.000
Tmin'	0.857	

Correction method= # Reported T Limits: Tmin=0.873 Tmax=1.000
AbsCorr = MULTI-SCAN

Data completeness= 1.72/0.96 Theta(max)= 77.084

R(reflections)= 0.0275(6787) wR2(reflections)= 0.0722(7030)

S = 1.031 Npar= 458

```
The following ALERTS were generated. Each ALERT has the format
       test-name_ALERT_alert-type_alert-level.
Click on the hyperlinks for more details of the test.

Alert level C
PLAT042_ALERT_1_C Calc. and Reported MoietyFormula Strings  Differ     Please Check
PLAT220_ALERT_2_C NonSolvent    Resd 2   C    Ueq(max)/Ueq(min) Range        3.9 Ratio
PLAT413_ALERT_2_C Short Inter XH3 .. XHn      H4        ..H42C      .       2.11 Ang.
                                              -x,1/2+y,1/2-z  =      3_555 Check
PLAT415_ALERT_2_C Short Inter D-H..H-X        H31       ..H49       .       2.14 Ang.
                                                       x,y,z  =      1_555 Check

Alert level G
PLAT007_ALERT_5_G Number of Unrefined Donor-H Atoms ..............          5 Report
PLAT045_ALERT_1_G Calculated and Reported Z Differ by a Factor ...      0.500 Check
PLAT143_ALERT_4_G s.u. on c - Axis Small or Missing .............. 0.00020 Ang.
PLAT912_ALERT_4_G Missing # of FCF Reflections Above STh/L=  0.600         81 Note
PLAT978_ALERT_2_G Number C-C Bonds with Positive Residual Density.         15 Info

   0 ALERT level A = Most likely a serious problem - resolve or explain
   0 ALERT level B = A potentially serious problem, consider carefully
   4 ALERT level C = Check. Ensure it is not caused by an omission or oversight
   5 ALERT level G = General information/check it is not something unexpected

   2 ALERT type 1 CIF construction/syntax error, inconsistent or missing data
   4 ALERT type 2 Indicator that the structure model may be wrong or deficient
   0 ALERT type 3 Indicator that the structure quality may be low
   2 ALERT type 4 Improvement, methodology, query or suggestion
   1 ALERT type 5 Informative message, check
```

It is advisable to attempt to resolve as many as possible of the alerts in all categories. Often the minor alerts point to easily fixed oversights, errors and omissions in your CIF or refinement strategy, so attention to these fine details can be worthwhile. In order to resolve some of the more serious problems it may be necessary to carry out additional measurements or structure refinements. However, the purpose of your study may justify the reported deviations and the more serious of these should normally be commented upon in the discussion or experimental section of a paper or in the "special_details" fields of the CIF. checkCIF was carefully designed to identify outliers and unusual parameters, but every test has its limitations and alerts that are not important in a particular case may appear. Conversely, the absence of alerts does not guarantee there are no aspects of the results needing attention. It is up to the individual to critically assess their own results and, if necessary, seek expert advice.

Publication of your CIF in IUCr journals

A basic structural check has been run on your CIF. These basic checks will be run on all CIFs submitted for publication in IUCr journals (*Acta Crystallographica*, *Journal of Applied Crystallography*, *Journal of Synchrotron Radiation*); however, if you intend to submit to *Acta Crystallographica Section C* or *E* or *IUCrData*, you should make sure that full publication checks are run on the final version of your CIF prior to submission.

Publication of your CIF in other journals

Please refer to the *Notes for Authors* of the relevant journal for any special instructions relating to CIF submission.

Figure S35. X-ray crystallographic analysis of dendrobine A (**2**).

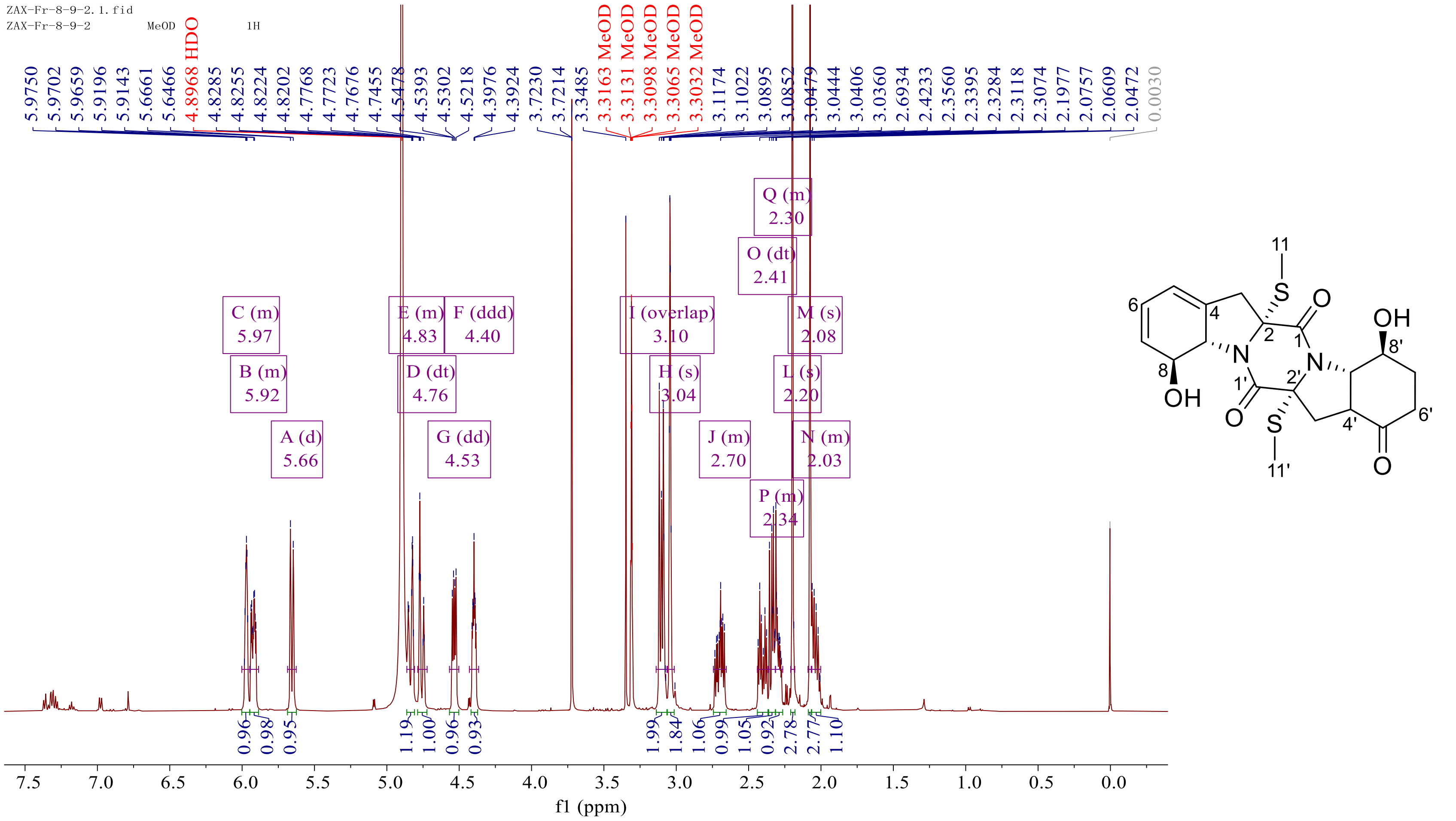

Figure S36. ¹H-NMR spectrum of (500 MHz) dendrobine B (**3**).

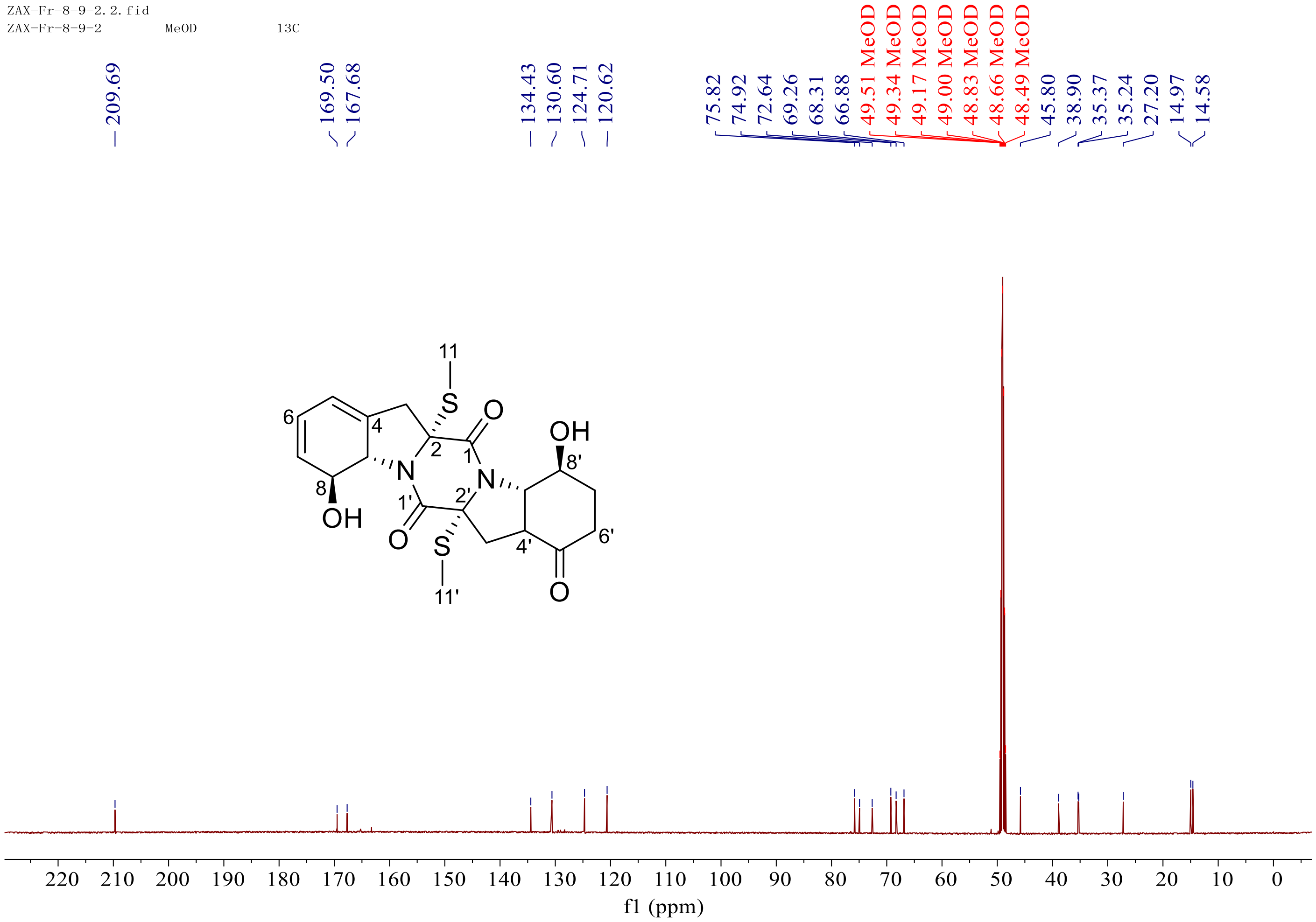

Figure S37. ^{13}C-NMR spectrum of (125 MHz) dendrobine B (**3**).

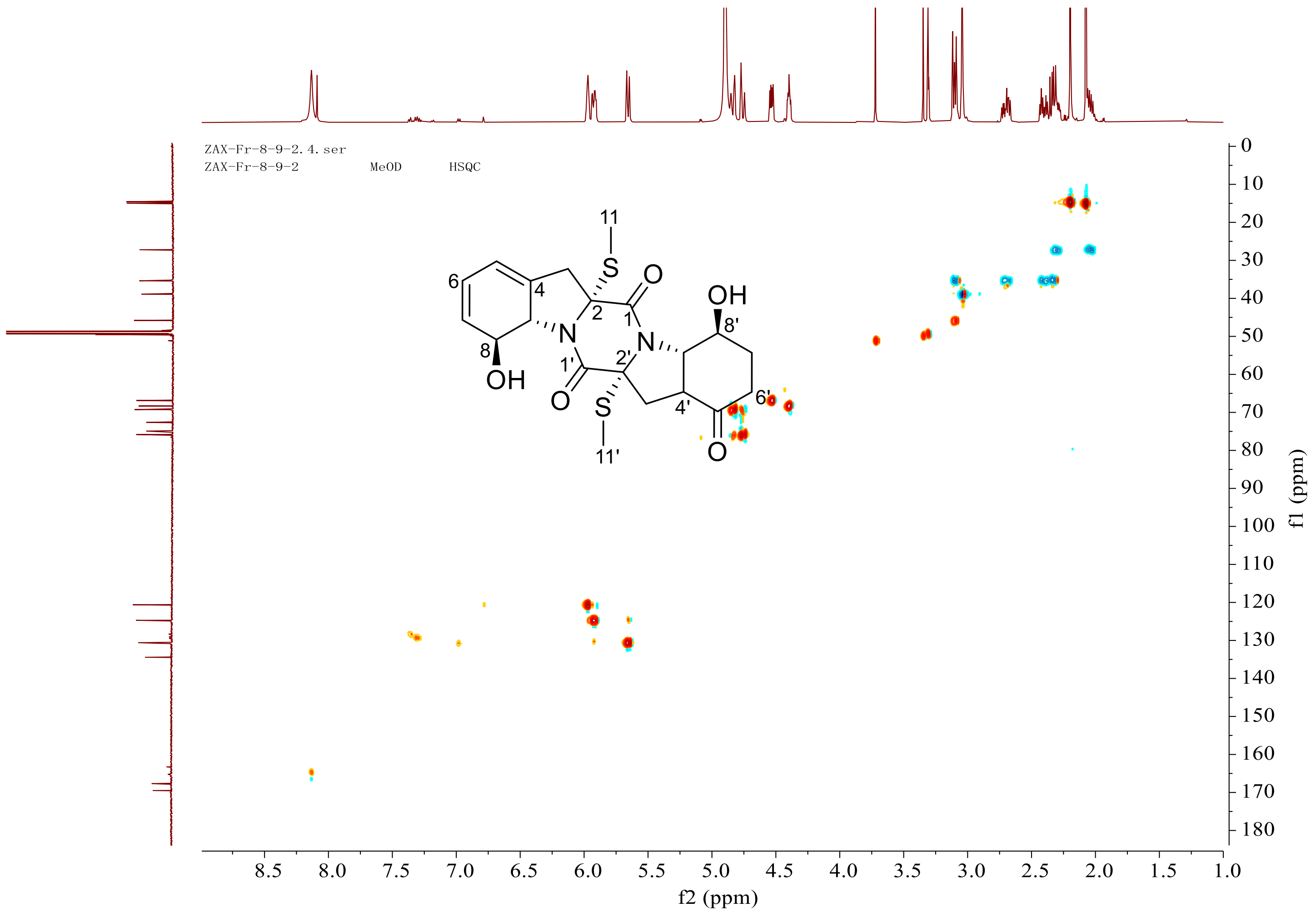

Figure S38. HSQC spectrum of dendrobine B (**3**).

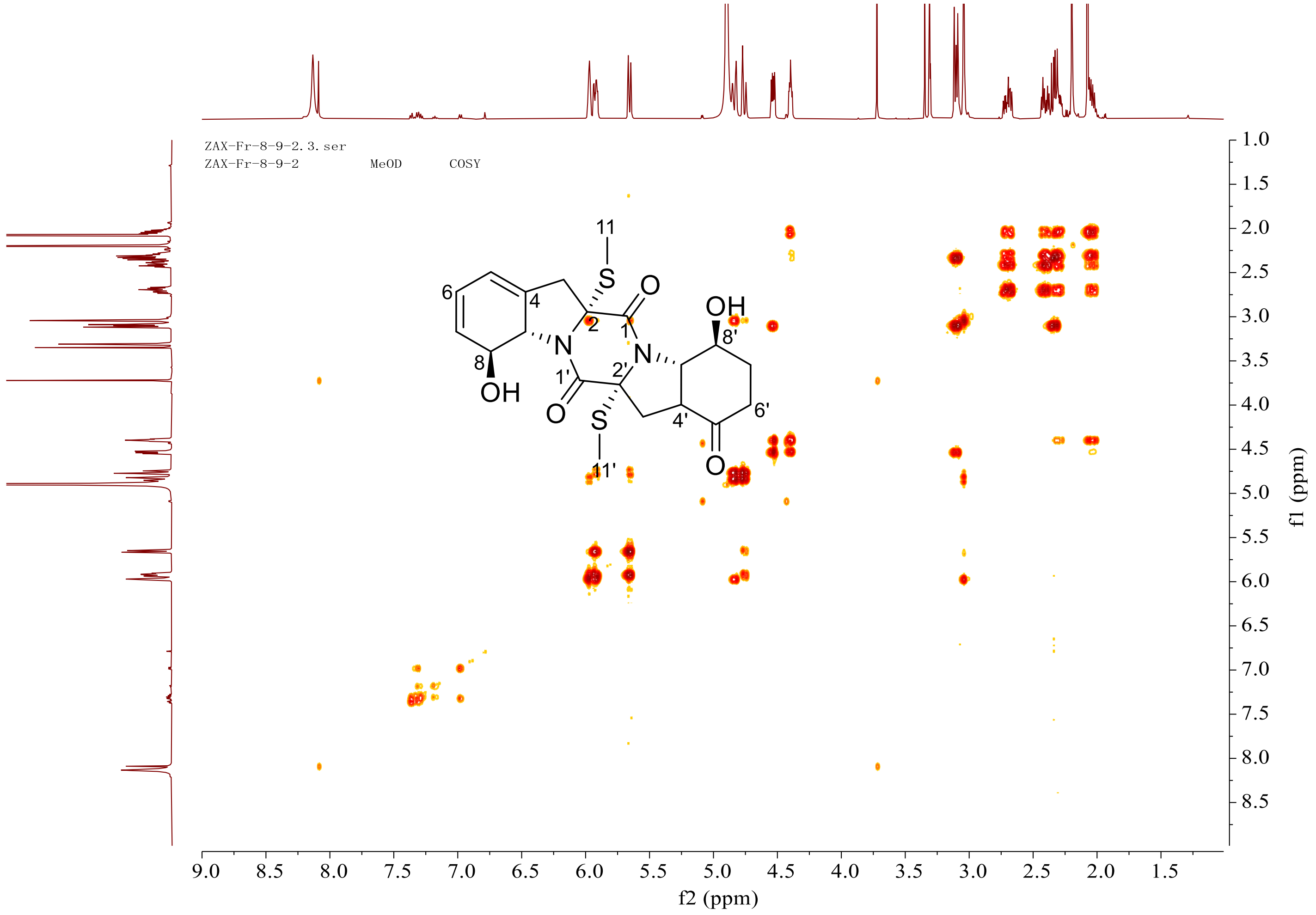

Figure S39. COSY spectrum of dendrobine B (**3**).

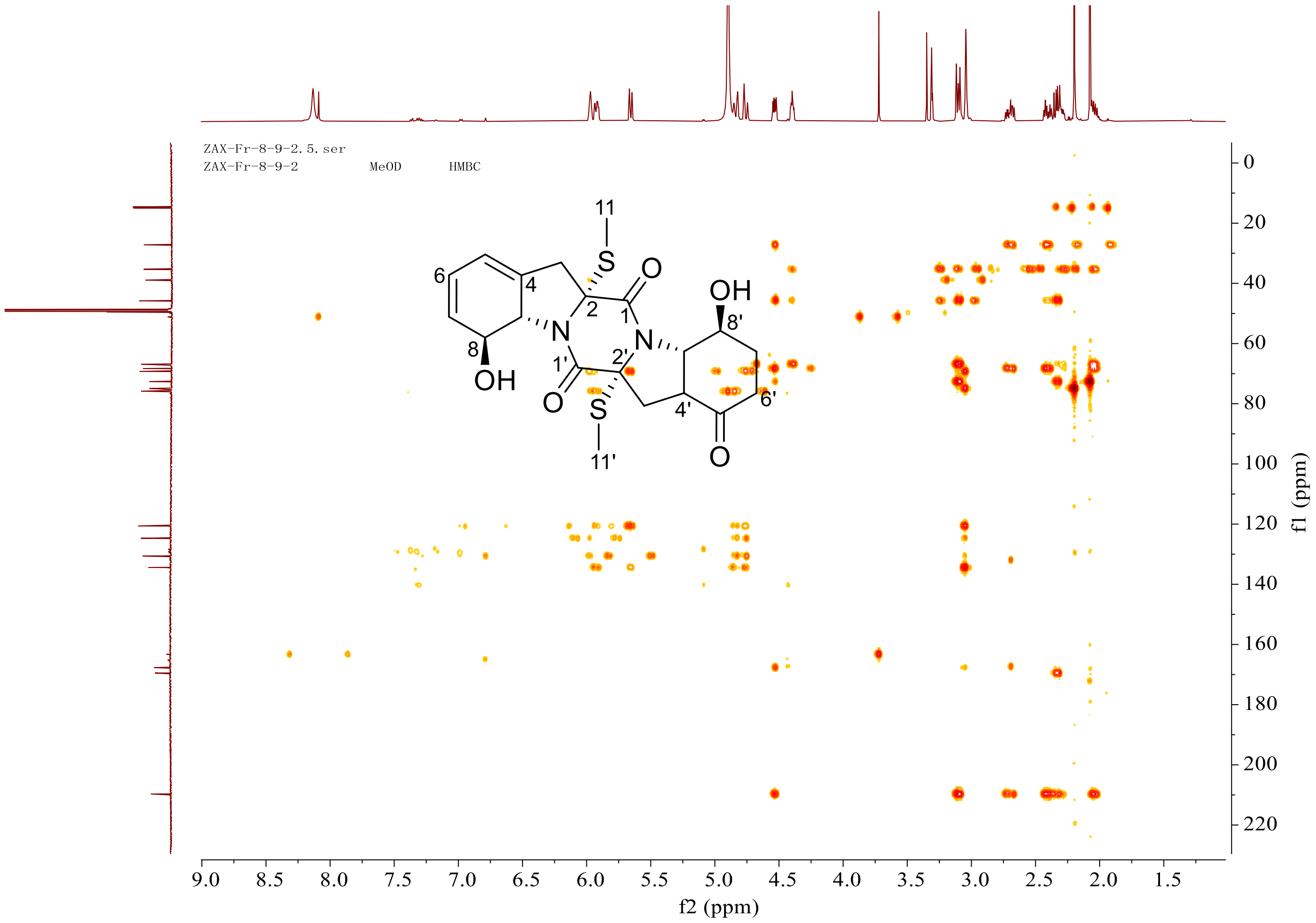

Figure S40. HMBC spectrum of dendrobine B (**3**).

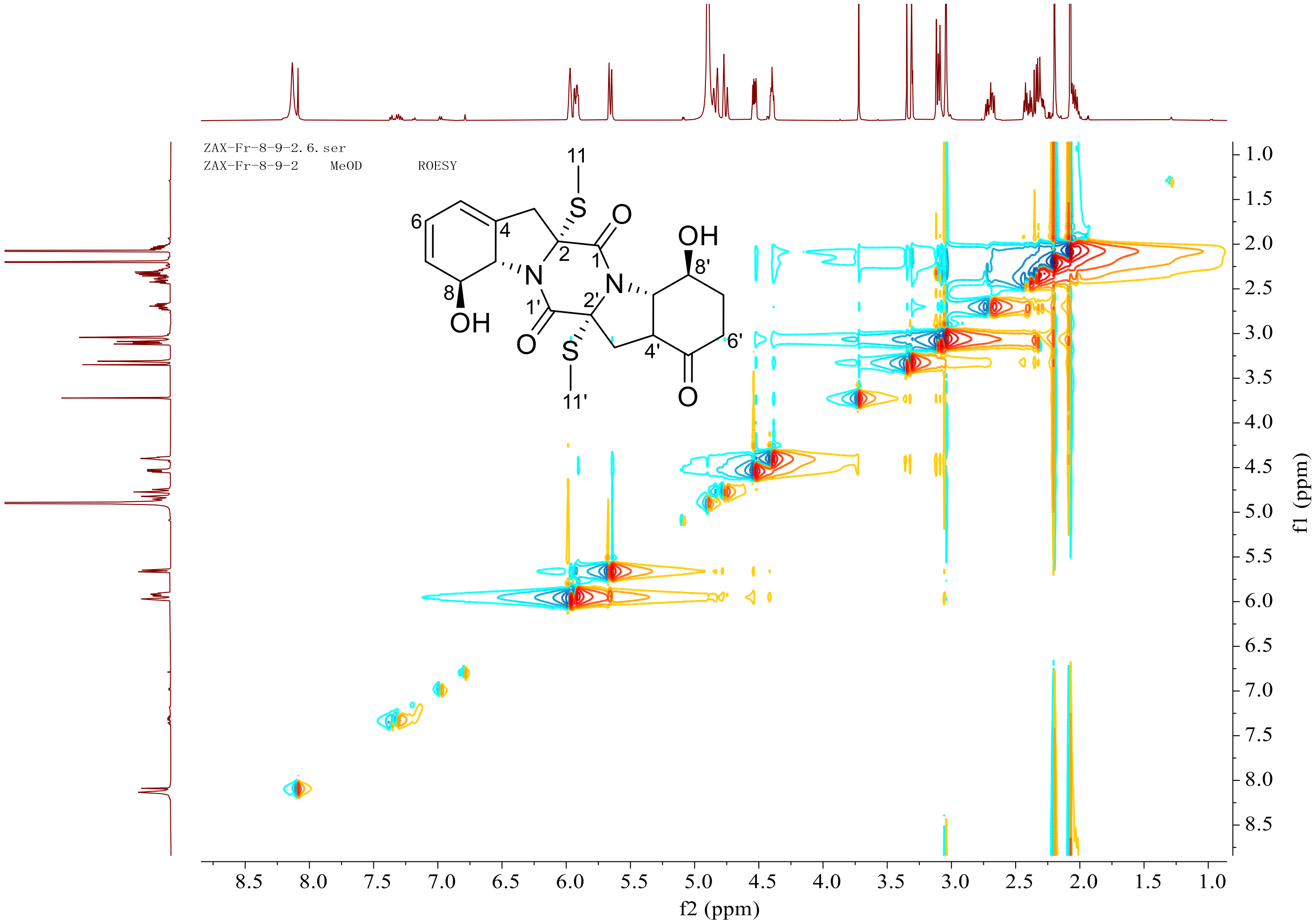

56

57 Figure S41. ROESY spectrum of dendrobine B (**3**)

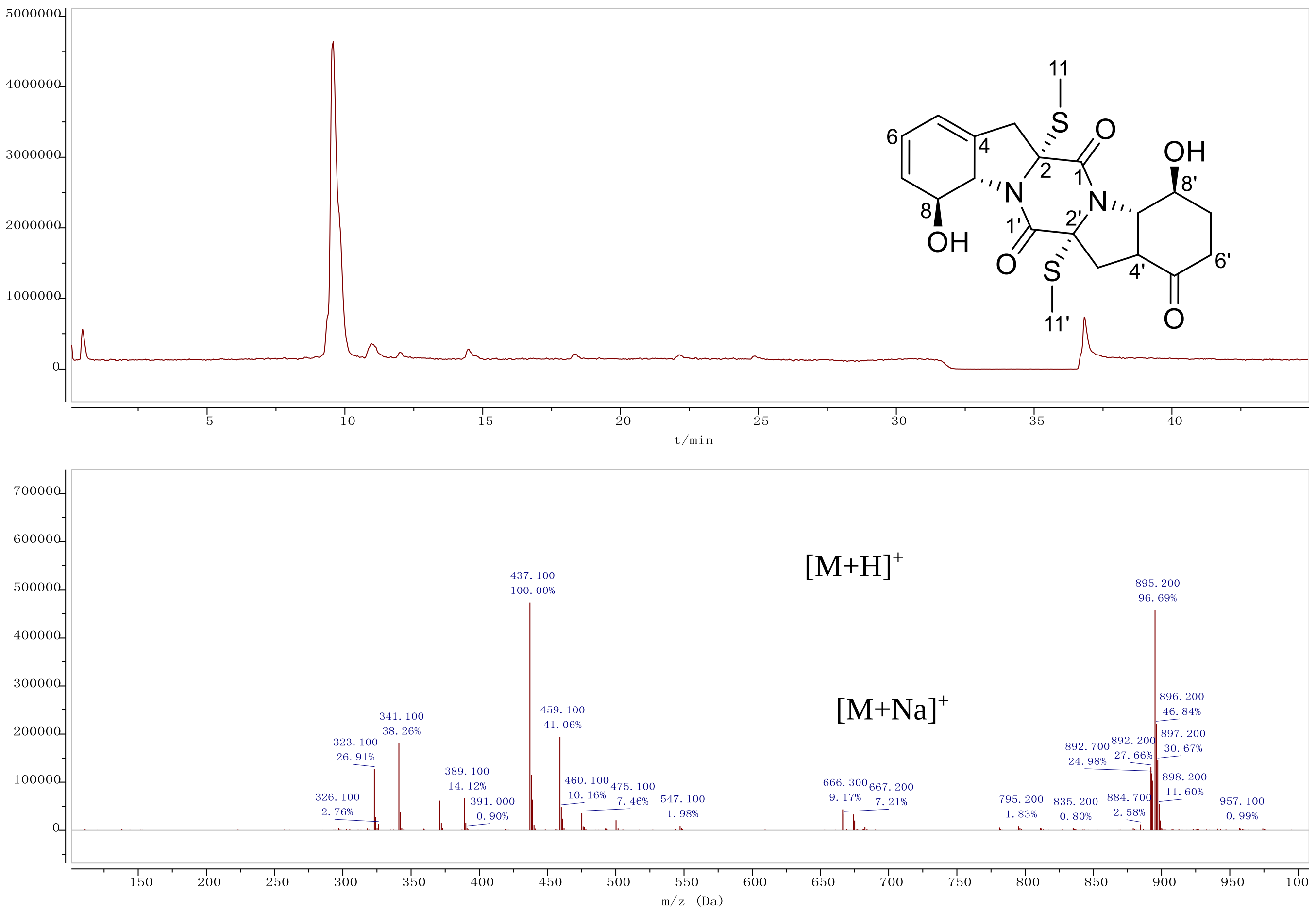

Figure S42. LC-MS spectrum of dendrobine B (**3**).

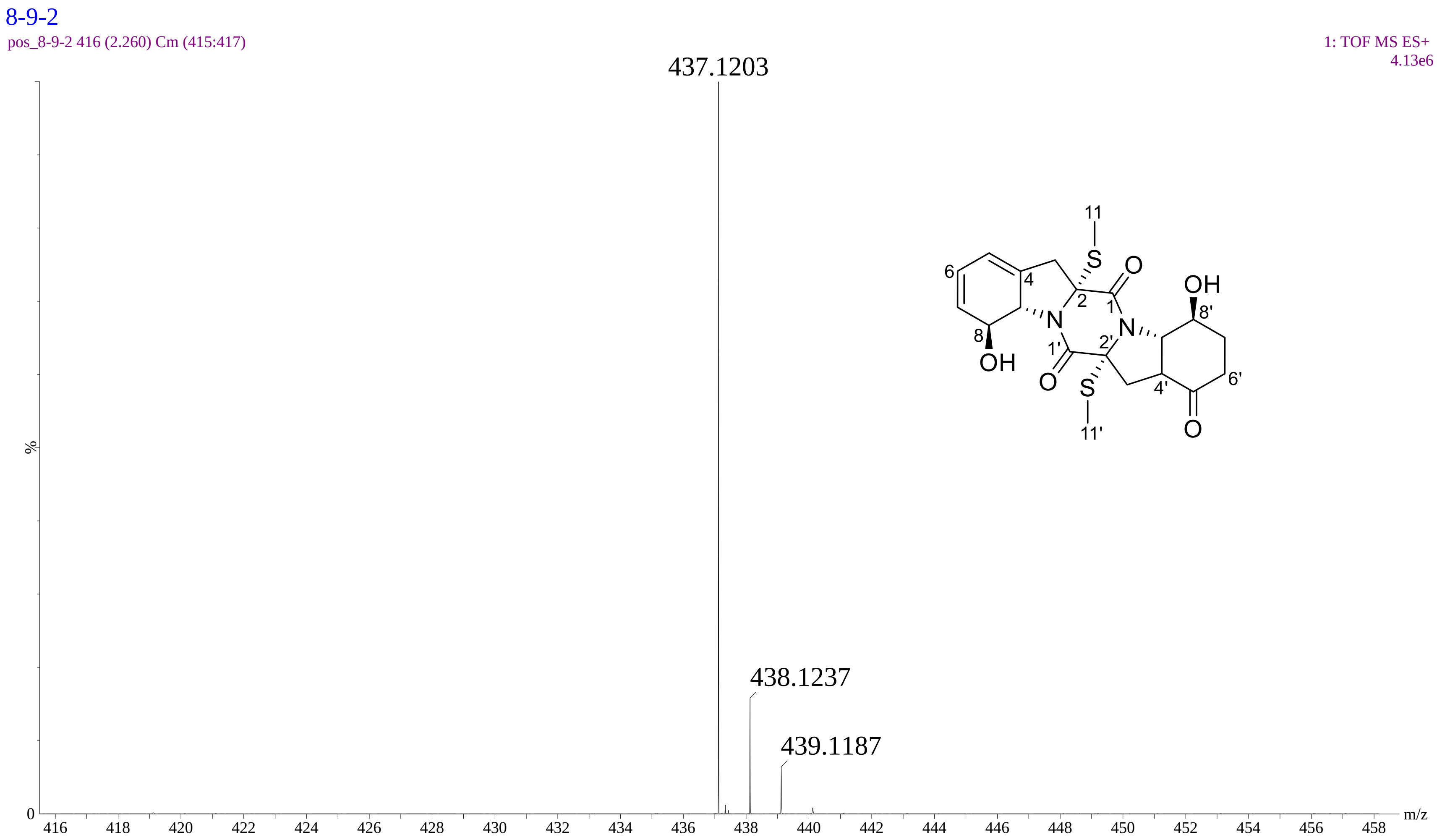

Figure S43. HRMS spectrum of dendrobine B (**3**).

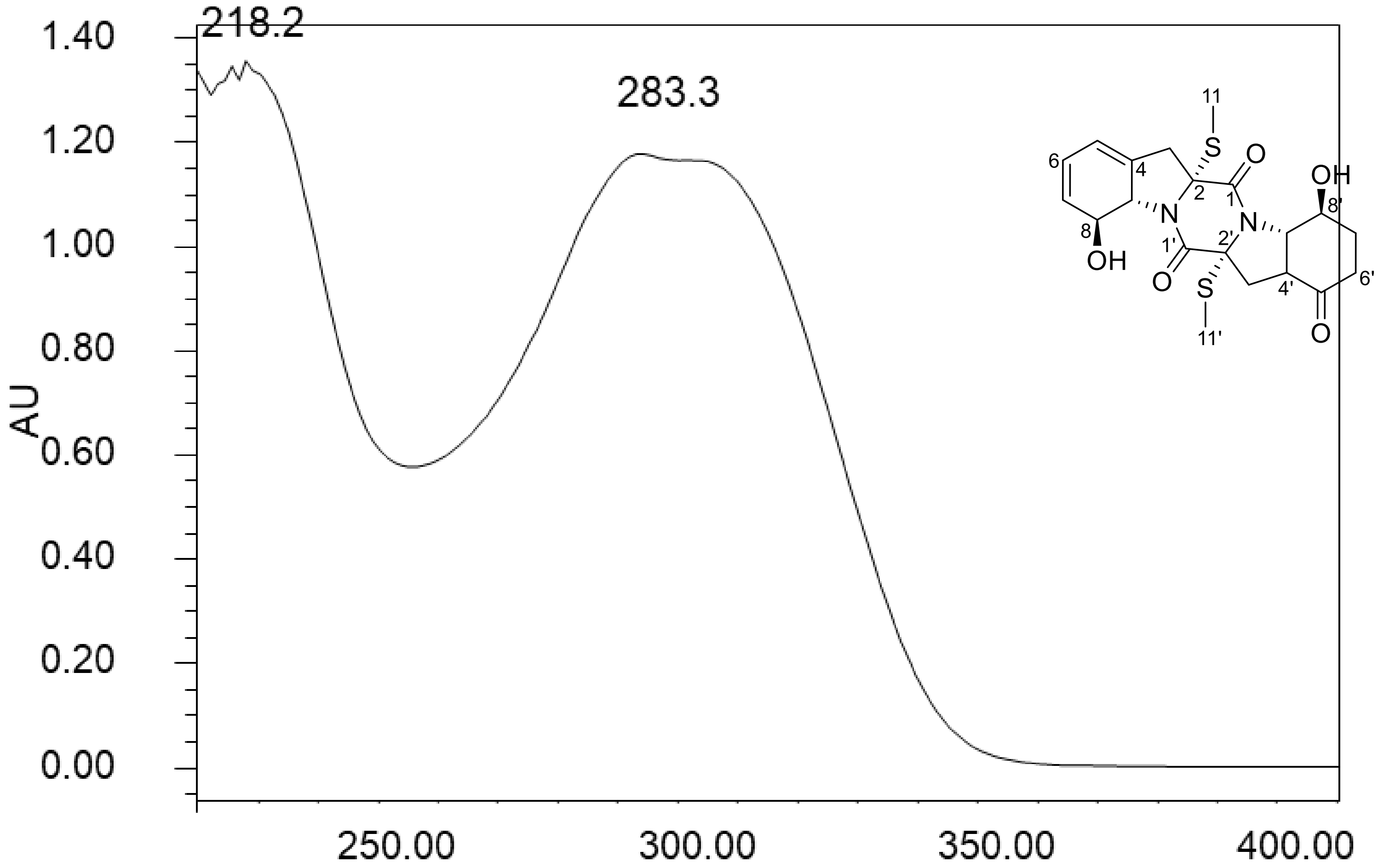

63
64 Figure S44. UV spectrum of dendrobine B (**3**) in CH_3OH.

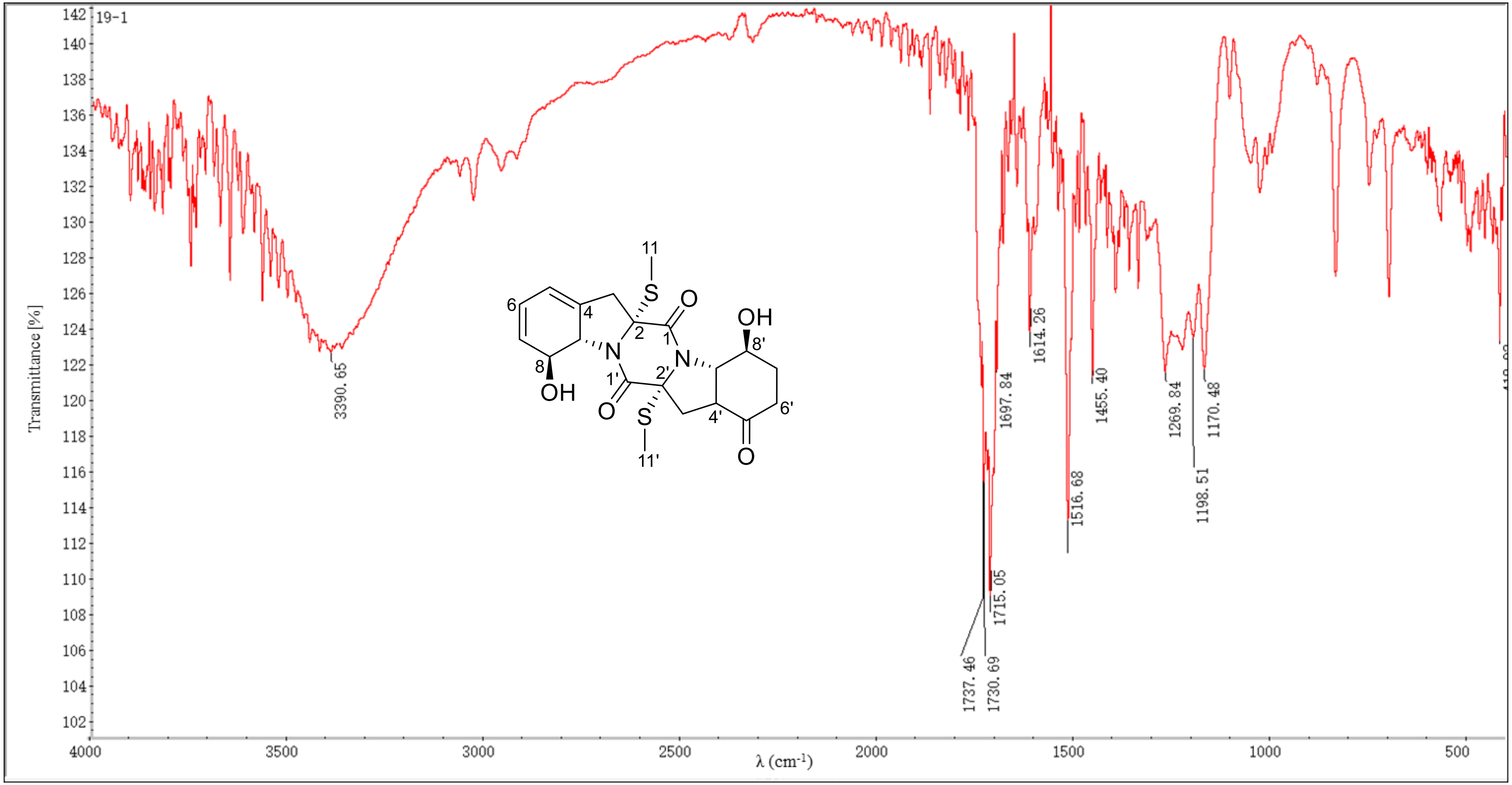

Figure S45. IR spectrum of dendrobine B (**3**).

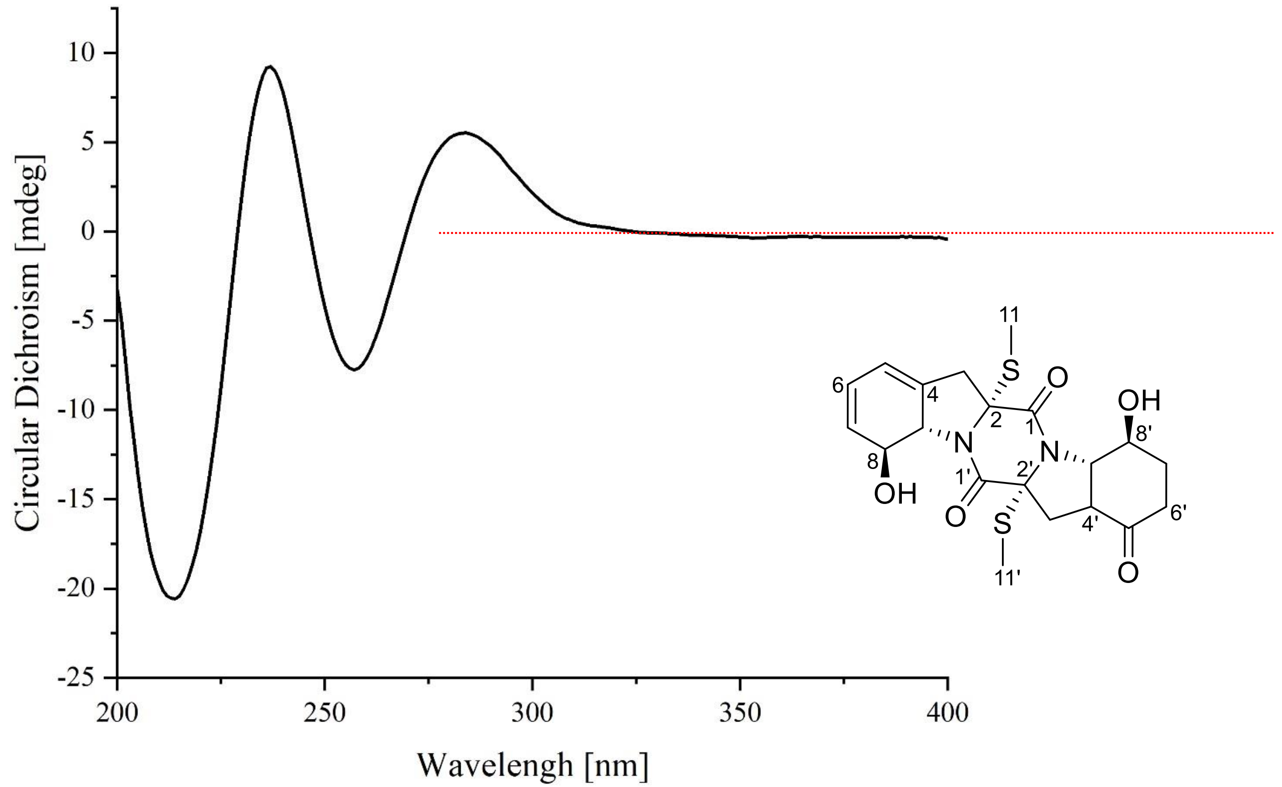

Figure S46. CD spectrum of dendrobine B (**3**) in CH_3OH.

Table S1. DNA assembly tool box constructed in this study

Plasmids	Description	
pYXR56	*pUC19-BsaI-mutation*	Destination vectors after removal of the type IIS restriction endonuclease recognition site using point mutation
pYXR57	*pET28a Esp3I mutation*	
pYXR81	*XW55-Cm*	
pYXR58	*XW55-Cm-BsaI+Esp3I mutation*	
pYXR59	*pYH-WA-BsaI mutation*	
pYXR60	*pYXR56 added BbsI and BsaI(forward)*	Level 0 destination vectors
pYXR61	*pYXR56 added BbsI and BsaI(reverse)*	
pYXR62	*pYXR57 added Esp 3I and BsaI(TGGA-GCTG)*	Level 1 destination vectors
pYXR63	*pYXR57 added Esp 3I and BsaI(GCTG-ATCT)*	
pYXR64	*pYXR57 added Esp 3I and BsaI(ATCT-TCAT)*	
pYXR65	*pYXR57 added Esp 3I and BsaI(TCAT-CTGG)*	
pYXR66	*pYXR57 added Esp 3I and BsaI(CTGG-TGTG)*	
pYXR67	*pYXR57 added Esp 3I and BsaI(TGTG-GAGT)*	
pYXR68	*pYXR57 added Esp 3I and BsaI(GAGT-TGGA)*	
pYXR69	*pYXR58 added BsaI and Esp 3I(TGGA-GCTG)*	Level 2 destination vectors
pYXR70	*pYXR58 added BsaI and Esp 3I(TGGA-ATCT)*	
pYXR71	*pYXR58 added BsaI and Esp 3I(TGGA-TCAT)*	
pYXR72	*pYXR58 added BsaI and Esp 3I(TGGA-CTGG)*	
pYXR73	*pYXR58 added BsaI and Esp 3I(TGGA-TGTG)*	
pYXR74	*pYXR58 added BsaI and Esp 3I(GCCT-TGGA-GAGT-GCTC)*	
pYXR75	*pYXR58 added BsaI and Esp 3I(GCTC-TGGA-GAGT-GCTG)*	
pYXR76	*pYXR58 added BsaI and Esp 3I(GCTG-TGGA-AGT-CGCT)*	
pYXR77	*pYXR57 added BsaI(GCTC-TGTG)*	Linker vectors
pYXR78	*pYXR57 added BsaI(GCTG-TGTG)*	
pYXR79	*pYXR57 added BsaI(CGCT-TGTG)*	
pYXR80	*pYXR59 added BsaI(GCCT-TGTG)*	Level 2i destination vectors
pYXR85	*GCTC*::P_{stcA}::*AATG*	Level 0 promoter library (These promoters were *forward* promoters in the AflR unit)
pYXR86	*GCTC*::P_{stcF}::*AATG*	
pYXR87	*GCTC*::P_{stcH}::*AATG*	
pYXR88	*GCTC*::P_{stcJ}::*AATG*	
pYXR89	*GCTC*::P_{stcL}::*AATG*	
pYXR90	*GCTC*::P_{stcM}::*AATG*	
pYXR91	*GCTC*::P_{stcO}::*AATG*	
pYXR92	*GCTC*::P_{stcP}::*AATG*	
pYXR93	*GCTC*::P_{stcQ}::*AATG*	
pYXR94	*GCTC*::P_{stcS}::*AATG*	
pYXR95	*GCTC*::P_{stcU}::*AATG*	
pYXR96	*GCTC*::P_{stcV}::*AATG*	
pYXR97	*CATT*::P_{stcB}::*CGCT*	Level 0 promoter library (These promoters were *reverse* promoters in the AflR unit)
pYXR98	*CATT*::P_{aflS}::*CGCT*	
pYXR99	*CATT*::P_{stcE}::*CGCT*	
pYXR100	*CATT*::P_{stcG}::*CGCT*	
pYXR101	*CATT*::P_{stcI}::*CGCT*	
pYXR102	*CATT*::P_{stcK}::*CGCT*	
pYXR103	*CATT*::P_{stcN}::*CGCT*	
pYXR104	*CATT*::P_{stcR}::*CGCT*	
pYXR105	*CATT*::P_{stcT1}::*CGCT*	
pYXR106	*CATT*::P_{stcT}::*CGCT*	
pYXR107	*CATT*::P_{stcW}::*CGCT*	
pYXR108	*GCTC*::P_{pfmaE}::*AATG*	Level 0 promoter library (These promoters were *forward* promoters in the PfmaH unit)
pYXR110	*GCTC*::P_{pfmaD}::*AATG*	
pYXR111	*CATT*::P_{pfmaG} ::*CGCT*	Level 0 promoter library (These promoters were *reverse* promoters in
pYXR112	*CATT*::P_{pfmaJ} ::*CGCT*	
pYXR113	*CATT*::P_{pfmaI} ::*CGCT*	

the PfmaH unit)

Table S2. Fungal strains used in this study

Strains	Description	Uses
Aspergillus nidulans LO8030	pyroA4, riboB2, pyrG89, nkuA::argB, sterigmatocystin cluster (AN7804-AN7825)Δ, emericellamide cluster (AN2545-AN2549)Δ, asperfuranone cluster (AN1039-AN1029)Δ, monodictyphenone cluster (AN10023-AN10021) Δ, terrequinone cluster (AN8512-8520)Δ, austinol cluster part 1 (AN8379-AN8384)Δ, austinol cluster part 2 (AN9246-9259)Δ, F9775 cluster (AN7906-7915)Δ, asperthecin cluster (AN6000-AN6002)Δ	Starting strain of *Aspergillus nidulans* cell factory [92]
Pestalotiopsis fici CGMCC3.15140	Wild type	Amplification of target fragment [93]
Penicillium brevicompactum	Wild type	Amplification of gene cluster of mycophenolic acid
Fusarium proliferatum	Wild type	Amplification of gene cluster of beauvericin
Epicoccum dendrobii	Wild type	Amplification of *eda* cluster of beauvericin
TWY1.1	pyroA4, pyrG89, ΔwApks::P_{gpdA}::TESG_06706 to 6702::AfpyrG, ΔnkuA::argB, veA	Strain overexpressing *nscR* [70]
TYWL8	wA flanking; pyrG	As a control for the white spore of *A. nidulans*
TYXR27	yA flanking; ribo，P_{gpdA} ::pfmaH	
TYXR203	yA flanking; ribo，P_{gpdA} ::aflR	
TYXR114	ST flanking, P_{alcA}::aflR::mcherry::TrpC::PyroA; wA flanking, nsc cluster (TESG_6702-TESG_6705)	Strain expressing *nsc* gene cluster without PSTF
TYXR115	ST flanking, P_{alcA}::aflR::mcherry::TrpC::PyroA; wA flanking, PstcA::TESG_6702::TESG_6703::PstcW::TESG_6704::PstcI::PstcV::TESG_6705	Strain expressing recombinant *nsc* gene clusters
TYXR140	Pyr4; fpbeas::kivr::AMAI	
TYXR143	yA flanking, AfRibo:: P_{gpdA} ::PfmaH; WA flanking, PyrG, P_{PfmaE}::fpbeas::kivr::P_{PfmaG}	
TYXR144	yA flanking, AfRibo:: P_{gpdA}::PfmaH; WA flanking, PyrG, fpbeas:: P_{PfmaG} :: P_{PfmaE} ::kivr	
TYXR145	yA flanking, AfRibo:: P_{gpdA}::PfmaH; WA flanking, PyrG, kivr:: P_{PfmaG}:: P_{PfmaD}::fpbeas	
TYXR146	yA flanking, AfRibo:: P_{gpdA} ::PfmaH; WA flanking, PyrG, kivr:: P_{PfmaJ}:: P_{PfmaE} ::fpbeas	
TYXR147	yA flanking, AfRibo:: P_{gpdA} ::PfmaH; WA flanking, PyrG, kivr:: P_{PfmaI}:: P_{PfmaD}::fpbeas	
TYLSJ13	yA flanking; ribo，P_{gpdA} ::AflR; WA flanking, PyrG, kivr:: P_{stcA}:: P_{stcN}::fpbeas	
TYLSJ14	yA flanking; ribo，P_{gpdA} ::AflR; WA flanking, PyrG, kivr:: P_{stcN}:: P_{stcA}::fpbeas	
TYLSJ15	yA flanking; ribo，P_{gpdA} ::AflR; WA flanking, PyrG, kivr:: P_{stcN}:: P_{stcW}::fpbeas	
TYLSJ16	yA flanking; ribo，P_{gpdA} ::AflR; WA flanking, PyrG, kivr:: P_{stcW}:: P_{stcN}::fpbeas	
TYLSJ17	yA flanking; ribo，P_{gpdA} ::AflR; WA flanking, PyrG, kivr:: P_{stcN}:: P_{stcB}::fpbeas	
TYLSJ18	yA flanking; ribo，P_{gpdA} ::AflR; WA flanking, PyrG, kivr:: P_{stcB}:: P_{stcN}::fpbeas	
TYLSJ5	yA flanking; ribo，P_{gpdA} ::AflR; WA flanking, PyrG, P_{stcA}::ataP	
TYLSJ6	yA flanking; ribo，P_{gpdA} ::AflR; WA flanking, PyrG, P_{stcA}::edaF	
TYLSJ11	wA flanking, pyroA, P_{stcE}::mpaA:: P_{stcM}::mpaB:: P_{stcN}::mpaC:: P_{stcB}::mpaDE:: P_{stcW}::mpaF:: P_{stcI}::mpaG:: P_{stcA}::mpaH	

TYLSJ12	*wA flanking, pyroA,* P_{stcI}*::mpaA::* P_{stcE}*::mpaB::* P_{stcW}*::mpaC::* P_{stcA}*::mpaDE::* P_{stcM}*::mpaF::* P_{stcB}*::mpaG::* P_{stcN}*::mpaH*
TYXR276	*yA flanking; ribo,* P_{gpdA} *::AflR; WA flanking, PyrG,* P_{stcA}*::ataP;* P_{stcI}*::edaJ::*P_{stcM}*::edaB::*P_{stcN}*::edaG::*P_{stcV}*::edaI::*P_{stcW}*::edaC::*P_{stcB}*::edaA; AMAI, pyrG,* P_{stcM}*::edaM::*P_{stcN}*::edaT::*P_{stcA}*::edaZ::*P_{stcB}*::edaS::*P_{stcV}*::edaF::*P_{stcW}*::edaN*
TYLSJ19	*yA flanking, AfRibo,* P_{alcA}*::PfmaH; wA flanking, PyrG,* P_{pfmaD}*::sfGFP*
TYLSJ20	*yA flanking, AfRibo,* P_{alcA}*::PfmaH; wA flanking, PyrG,* P_{pfmaE}*::sfGFP*

Table S3. Dual-fluorescent labeled strains constructed in this study

Strains	Description	Uses
TYXR113	*yA flanking, AfRibo, P_{alcA}::PfmaH::mCherry*	Expression strains of PfmaH labeled with red fluorescence
TYXR111	*ST flanking, P_{alcA}::aflR::mCherry::TrpC::PyroA*	Expression strains of AflR labeled with red fluorescence
TYXR128	*WA flanking, PyrG, P_{pfmaD}::sfGFP*	Control strains, fusing the promoter of the PfmaH system with sfGFP without expressing the transcription factor PfmaH
TYXR129	*WA flanking, PyrG, P_{pfmaE}::sfGFP*	
TYXR131	*WA flanking, PyrG, P_{pfmaG}::sfGFP*	
TYXR134	*WA flanking, PyrG, P_{pfmaI}::sfGFP*	
TYXR74	*yA flanking, AfRibo, P_{alcA}::PfmaH::mCherry; wA flanking, PyrG, P_{pfmaD}::sfGFP*	Dual-fluorescence labeled strains of the PfmaH transcriptional regulatory unit
TYXR75	*yA flanking, AfRibo, P_{alcA}::PfmaH::mCherry; wA flanking, PyrG, P_{pfmaE}::sfGFP*	
TYXR77	*yA flanking, AfRibo, P_{alcA}::PfmaH::mCherry; wA flanking, PyrG, P_{pfmaG}::sfGFP*	
TYXR78	*yA flanking, AfRibo, P_{alcA}::PfmaH::mCherry; wA flanking, PyrG, P_{pfmaI}::sfGFP*	
TYXR79	*yA flanking, AfRibo, P_{alcA}::PfmaH::mCherry; wA flanking, PyrG, P_{pfmaJ}::sfGFP*	
TYXR84	*ST flanking, PyroA, P_{alcA}::aflR::mCherry; P_{stcA}::sfGFP*	Dual-fluorescence labeled strains of the AflR transcriptional regulatory unit
TYXR85	*ST flanking, PyroA, P_{alcA}::aflR::mCherry; P_{stcB}::sfGFP*	
TYXR86	*ST flanking, PyroA, P_{alcA}::aflR::mCherry; P_{stcC}::sfGFP*	
TYXR87	*ST flanking, PyroA, P_{alcA}::aflR::mCherry; P_{stcD}::sfGFP*	
TYXR88	*ST flanking, PyroA, P_{alcA}::aflR::mCherry; P_{stcE}::sfGFP*	
TYXR89	*ST flanking, PyroA, P_{alcA}::aflR::mCherry; P_{stcF}::sfGFP*	
TYXR90	*ST flanking, PyroA, P_{alcA}::aflR::mCherry; P_{stcG}::sfGFP*	
TYXR91	*ST flanking, PyroA, P_{alcA}::aflR::mCherry; P_{stcH}::sfGFP*	
TYXR92	*ST flanking, PyroA, P_{alcA}::aflR::mCherry; P_{stcI}::sfGFP*	
TYXR93	*ST flanking, PyroA, P_{alcA}::aflR::mCherry; P_{stcJ}::sfGFP*	
TYXR94	*ST flanking, PyroA, P_{alcA}::aflR::mCherry; P_{stcK}::sfGFP*	
TYXR95	*ST flanking, PyroA, P_{alcA}::aflR::mCherry; P_{stcL}::sfGFP*	
TYXR96	*ST flanking, PyroA, P_{alcA}::aflR::mCherry; P_{stcM}::sfGFP*	
TYXR97	*ST flanking, PyroA, P_{alcA}::aflR::mCherry; P_{stcN}::sfGFP*	
TYXR98	*ST flanking, PyroA, P_{alcA}::aflR::mCherry; P_{stcO}::sfGFP*	
TYXR99	*ST flanking, PyroA, P_{alcA}::aflR::mCherry; P_{stcP}::sfGFP*	
TYXR100	*ST flanking, PyroA, P_{alcA}::aflR::mCherry; P_{stcQ}::sfGFP*	
TYXR101	*ST flanking, PyroA, P_{alcA}::aflR::mCherry; P_{stcR}::sfGFP*	
TYXR102	*ST flanking, PyroA, P_{alcA}::aflR::mCherry; P_{stcS}::sfGFP*	
TYXR103	*ST flanking, PyroA, P_{alcA}::aflR::mCherry; P_{stcT}::sfGFP*	
TYXR104	*ST flanking, PyroA, P_{alcA}::aflR::mCherry; P_{stcU}::sfGFP*	

TYXR105	*ST flanking, PyroA, P_{alcA}::aflR::mCherry; P_{stcV}::sfGFP*
TYXR106	*ST flanking, PyroA, P_{alcA}::aflR::mCherry; P_{stcW}::sfGFP*
TYXR107	*ST flanking, PyroA, P_{aflS}::aflR::mCherry; P_{aflS}::sfGFP*
TYXR108	*ST flanking, PyroA, P_{alcA}::aflR::mCherry; P_{stcT1}::sfGFP*

Table S4. Plasmids used in this study

Plasmids	Description	Uses
pYH-wA	*URA3, WA flanking, AfpyrG, Amp*	Original plasmid, template plasmids for amplification of screening marker genes
pYWB2	*URA3, WA flanking, AfRibo, Amp*	
pYW25.16	*URA3, WA flanking, Amp, AfpyroA*::P_{gpdA}	
pYZM6	*URA3, yA flanking, Afribo, Amp*	Departure vector for constructing fixed-point inserts
pYZM8	*URA3, WA flanking, AfpyrG, sfGFP ::trpC Amp*	
pYXR27	*URA3, WA flanking, AfpyrG, Amp,* P_{stcA}::*sfGFP*	Promoters in the AflR unit were fused to sfGFP
pYXR28	*URA3, WA flanking, AfpyrG, Amp,* P_{stcB}::*sfGFP*	
pYXR29	*URA3, WA flanking, AfpyrG, Amp,* P_{stcC}::*sfGFP*	
pYXR30	*URA3, WA flanking, AfpyrG, Amp,* P_{stcD}::*sfGFP*	
pYXR33	*URA3, WA flanking, AfpyrG, Amp,* P_{stcE}::*sfGFP*	
pYXR32	*URA3, WA flanking, AfpyrG, Amp,* P_{stcF}::*sfGFP*	
pYXR33	*URA3, WA flanking, AfpyrG, Amp,* P_{stcG}::*sfGFP*	
pYXR34	*URA3, WA flanking, AfpyrG, Amp,* P_{stcH}::*sfGFP*	
pYXR35	*URA3, WA flanking, AfpyrG, Amp,* P_{stcI}::*sfGFP*	
pYXR36	*URA3, WA flanking, AfpyrG, Amp,* P_{stcJ}::*sfGFP*	
pYXR37	*URA3, WA flanking, AfpyrG, Amp,* P_{stcK}::*sfGFP*	
pYXR38	*URA3, WA flanking, AfpyrG, Amp,* P_{stcL}::*sfGFP*	
pYXR39	*URA3, WA flanking, AfpyrG, Amp,* P_{stcM}::*sfGFP*	
pYXR40	*URA3, WA flanking, AfpyrG, Amp,* P_{stcN}::*sfGFP*	
pYXR41	*URA3, WA flanking, AfpyrG, Amp,* P_{stcO}::*sfGFP*	
pYXR42	*URA3, WA flanking, AfpyrG, Amp,* P_{stcP}::*sfGFP*	
pYXR43	*URA3, WA flanking, AfpyrG, Amp,* P_{stcQ}::*sfGFP*	
pYXR44	*URA3, WA flanking, AfpyrG, Amp,* P_{stcR}::*sfGFP*	
pYXR45	*URA3, WA flanking, AfpyrG, Amp,* P_{stcS}::*sfGFP*	
pYXR46	*URA3, WA flanking, AfpyrG, Amp,* P_{stcT}::*sfGFP*	
pYXR47	*URA3, WA flanking, AfpyrG, Amp,* P_{stcU}::*sfGFP*	
pYXR48	*URA3, WA flanking, AfpyrG, Amp,* P_{stcV}::*sfGFP*	
pYXR49	*URA3, WA flanking, AfpyrG, Amp,* P_{stcW}::*sfGFP*	
pYXR50	*URA3, WA flanking, AfpyrG, Amp,* P_{aflS}::*sfGFP*	
pYXR51	*URA3, WA flanking, AfpyrG, Amp,* P_{stcT1}::*sfGFP*	
pYPZ77	*URA3, wA flanking, Afribo, Amp,* P_{pfmaI}::*sfGFP*	Promoters in the AflR unit were fused to sfGFP
pYPZ78	*URA3, wA flanking, Afribo, Amp,* P_{pfmaD}::*sfGFP*	
pYPZ79	*URA3, wA flanking, Afribo, Amp,* P_{pfmaE}::*sfGFP*	
pYPZ81	*URA3, wA flanking, Afribo, Amp,* P_{pfmaG}::*sfGFP*	
pYXR52	*URA3, yA flanking, Afribo, Amp,* P_{pfmaJ}::*sfGFP*	
pYXR121	*ST flanking, PyroA,* P_{alcA}::*aflR*::*mCherry*::*TrpC*	Expression vector for aflR fusion mCherry
pYXR84	*URA3, yA flanking, Afribo, Amp* P_{alcA}::*pfmaH*::*mCherry*::*TrpC*	Expression vector for pfmaH fusion mCherry
pYXR114	P_{stcA}::*TESG_6702*	Level 1 vectors for *nsc* gene cluster recombination
pYXR115	P_{stcW}::*TESG_6703*	
pYXR116	P_{stcI}::*TESG_6704*	
pYXR117	P_{stcV}::*TESG_6705*	
pYXR118	*URA3, WA flanking, pyrG, Amp, TESG_6702-TESG_6705*	Heterologous expression vector for *nsc* gene cluster
pYXR119	*URA3, Cm,* P_{stcA}::*TESG_6702*::*TESG_6703*::P_{stcW}::*TESG_6704*::P_{stcI}::P_{stcV}::*TESG_6705*	Level 2 vectors for *nsc* gene cluster recombination
pYXR120	*URA3, wA flanking, PyrG,* P_{stcA}::*TESG_6702;* P_{stcW}::*TESG_6703;* P_{stcI}::*TESG_6704;* P_{stcV}::*TESG_6705*	Heterologous expression vector for recombination *nsc* gene cluster
pYXR145	*URA3, WA flanking, PyrG,* P_{PfmaE}::*fpbeas*::*kivr*::P_{PfmaG}	
pYXR146	*URA3, WA flanking, PyrG, fpbeas*::P_{PfmaG}:: P_{PfmaE}::*kivr*	
pYXR151	*URA3, WA flanking, PyrG, kivr*::P_{PfmaJ}:: P_{PfmaE}::*fpbeas*	
pYXR153	*URA3, WA flanking, PyrG, kivr*::P_{PfmaI}:: P_{PfmaD}::*fpbeas*	
pYLSJ13	*URA3, WA flanking, PyrG, kivr*::P_{stcA}:: P_{stcN}::*fpbeas*	
pYLSJ15	*URA3, WA flanking, PyrG, kivr*::P_{stcN}:: P_{stcW}::*fpbeas*	
pYLSJ16	*URA3, WA flanking, PyrG, kivr*::P_{stcW}:: P_{stcN}::*fpbeas*	

pYLSJ17	*URA3, WA flanking, PyrG, kivr*::P_{stcN}:: P_{stcB}::*fpbeas*
pYLSJ1	*URA3, WA flanking, PyrG, 15HA,* P_{stcA}::*ataP*
pYLSJ2	*URA3, WA flanking, PyrG, 15HA,* P_{stcA}::*edaF*
pYXR209	*AMAI, pyrG,* P_{stcM}::*edaM*::P_{stcN}::*edaT*::P_{stcA}::*edaZ*::P_{stcB}::*edaS*::P_{stcV}::*edaF*::P_{stcW}::*edaN*
pYXR210	*URA3, WA flanking, PyroA, 15HA,* P_{stcI}::*edaJ*::P_{stcM}::*edaB*::P_{stcN}::*edaG*::P_{stcV}::*edaI*::P_{stcW}::*edaC*::P_{stcB}::*edaA;*
pYLSJ5	*wA up, 15 HA, pyroA,* P_{stcE}::*mpaA*:: P_{stcM}::*mpaB*:: P_{stcN}::*mpaC*:: P_{stcB}::*mpaDE*
pYLSJ6	*wA flanking, pyrG,* P_{stcW}::*mpaF*:: P_{stcI}::*mpaG*:: P_{stcA}::*mpaH*
pYLSJ7	*wA up, 15 HA, pyroA,* P_{stcI}::*mpaA*:: P_{stcE}::*mpaB*:: P_{stcW}::*mpaC*:: P_{stcA}::*mpaDE*
pYLSJ8	*wA flanking, pyrG,* P_{stcM}::*mpaF*:: P_{stcB}::*mpaG*:: P_{stcN}::*mpaH*

Table S5. Primers used in this study

Name	Oligonucleotide sequence (5'-3')	Uses
ST-5F-F	GTGGTCCCTCCAGGAAGATAG	Construction of aflR/S expression cassette
ST-5F-R	GCTAGAGGTGCGATACGAACCGTCTGCTAGAAAGCACTAACCGCAAGCCAGTTTTATCG	
aflR/S-F	TTAGTGCTTTCTAGCAGACGGTTCG	
aflR/S-R	TCAGGCGTGGCGGAGGATG	
aflR/S-PyroA-F	GCGAAACGATCAGCATCCTCCGCCACGCCTGAGTAATGTAAGGTCAGTTCGAGACCATC	
aflR/S-PyroA-R	GATCGTAACTCCGTAGGATGTGTAC	
ST-3F-F	AGACGTCAAGGTACACATCCTACGGAGTTACGATCGTTTGGCTGGCTTAGTTCACAATG	
ST-3F-R	CCTGCTTGTCACGCATATCAG	
aflR/S-Nest-F	GCCAATCTAGCAATCAATGCTCG	
aflR/S-Nest-R	CGAGTACCGCATGGAACGTTG	
aflR/S-detect-5F-F	GACCTCATCCGGTCGTCAG	
aflR/S-detect-5F-R	CTTCCCGCCTGATGATAGC	
aflR/S-detect-F	GTCGACTGCCCAAGACAGTC	
aflR/S-detect-R	GGTGGAATTCTGGAGGATGGAG	
aflR/S-detect-3F-F	CAATGACGATCTCTTCCATGAAGAC	
aflR/S-detect-3F-R	CTAACACCCGGAGATCGC	
alcA-F	TTTGAGGCGAGGTGATAGGATTG	Primers for the construction of pYXR84
alcA-R	TAAGTCCCTTCGTATTTCTCCGC	
mcherry-F	ATGGTGAGCAAGGGCGAG	
mcherry-R	CTACTTGTACAGCTCGTCCATG	
pYXR84-trpC-F	TCCTATAAATTGGGAAAACTAGAGACCCCGGTCGCGGTAAACGACTCATAGGAGAGTTG	
pYXR84-7104-F	ATGATGGCCATGTTATCCTCCTCGCCCTTGCTCACCATGTGCCAGTTATTGCCTAGAGC	
pYXR84-7104-R	TCCACACAGGCGGAGAAATACGAAGGGACTTAATGCTGATGATTTCAAATTGTTCAAGG	
pYXR84-alcA-R	AGAATCCCAAACATCAACCCCGTCCAACAAGCGGCCTTTGAGGCGAGGTGATAGGATTG	
pYXR27-QC-F	CTCCTTCTCCTGATCGCTAGCTGGACTCGCAATCAGAGGG	Primers for the construction of sfGFP fusion plasmid
pYXR27-QC-R	CAGCTCCTCGCCCTTGCGCATCCTGGAGCCGTTTATTGCG	
pYXR28-QC-F	CTCCTTCTCCTGATCGCTAGCCCTGGAGCCGTTTATTGCG	
pYXR28-QC-R	CAGCTCCTCGCCCTTGCGCATTGGACTCGCAATCAGAGGG	
pYXR29-QC-F	TCTCCTTCTCCTGATCGCTAGCTTTGAAGTATTGTTTAACGGATTGGTGG	
pYXR29-QC-R	AGCTCCTCGCCCTTGCGCATTGTATAGCGAAAGTTGTGTC	
pYXR30-QC-F	CTTCTCCTGATCGCTAGCTGTATAGCGAAAGTTGTGTCGC	
pYXR30-QC-R	AACAGCTCCTCGCCCTTGCGCATTTTGAAGTATTGTTTAACGGATTGGTG	
pYXR31-QC-F	CTTCTCCTGATCGCTAGCACGACTAGTGTCGAGTTGAATC	
pYXR31-QC-R	ACAGCTCCTCGCCCTTGCGCATGGCGGCAGTACAGAACTG	
pYXR32-QC-F	TCCTTCTCCTGATCGCTAGCATTGAGTCCGGGTGAGTAGC	
pYXR32-QC-R	ACAGCTCCTCGCCCTTGCGCATGCCGAATAATGTTGGATTTTGGC	
pYXR33-QC-F	TCTCCTTCTCCTGATCGCTAGCGCCGAATAATGTTGGATTTTGGC	

pYXR33-QC-R	AGCTCCTCGCCCTTGCGCATATTGAGTCCGGGTGAGTAGC
pYXR34-QC-F	CTTCTCCTGATCGCTAGCTGTTGAAGCGATCTGTTGTCTG
pYXR34-QC-R	ACAGCTCCTCGCCCTTGCGCATACTGAGGACTCAGGGGGG
stc promoter QC-detect-F	GGCAAAAAGGACTGCATCAG
stc promoter QC-detect-R	GGTGGTGCAGATGAACTTGAG
pYXR35-QC-F	TCTCCTTCTCCTGATCGCTAGCACTGAGGACTCAGGGGGG
pYXR35-QC-R	CTCCTCGCCCTTGCGCATTGTTGAAGCGATCTGTTGTCTG
pYXR36-QC-F	ACTCTCCTTCTCCTGATCGCTAGCTCTCTTTACAGGTCGTCAAACTGTAG
pYXR36-QC-R	AGCTCCTCGCCCTTGCGCATTCCAATAAGCGTCTTGGCTG
pYXR37-QC-F	TCCTTCTCCTGATCGCTAGCTCCAATAAGCGTCTTGGCTG
pYXR37-QC-R	ACAGCTCCTCGCCCTTGCGCATTCTCTTTACAGGTCGTCAAACTG
pYXR38-QC-F	CCTTCTCCTGATCGCTAGCCTGAATGTACATAGGGCTGCC
pYXR38-QC-R	ACAGCTCCTCGCCCTTGCGCATCCTGCAAGACCATGGACC
pYXR39-QC-F	CTCCTTCTCCTGATCGCTAGCAGTGACGATGGTCTGTCTG
pYXR39-QC-R	TGAACAGCTCCTCGCCCTTGCGCATGGTTTTGAAGAGTTCCAGGAGTATG
pYXR40-QC-F	TCCTTCTCCTGATCGCTAGCGGTTTTGAAGAGTTCCAGGAGTATG
pYXR40-QC-R	CAGCTCCTCGCCCTTGCGCATAGTGACGATGGTCTGTCTG
pYXR41-QC-F	TGACTCTCCTTCTCCTGATCGCTAGCATTATTGTACTTCTACTTTAGGGTAGAATGGAG
pYXR41-QC-R	GGTGAACAGCTCCTCGCCCTTGCGCATCCTGATGTAGGATTAGGATAGAATGTATAGAG
pYXR42-QC-F	TTGACTCTCCTTCTCCTGATCGCTAGCCCTGATGTAGGATTAGGATAGAATGTATAGAG
pYXR42-QC-R	GTGAACAGCTCCTCGCCCTTGCGCATATTATTGTACTTCTACTTTAGGGTAGAATGGAG
pYXR43-QC-F	TCTCCTTCTCCTGATCGCTAGCGGCTACTGCATGCCATTCTATTC
pYXR43-QC-R	GTGAACAGCTCCTCGCCCTTGCGCATTGTTTGGTTCTGTGAGGGTATTTG
pYXR44-QC-F	CTCCTTCTCCTGATCGCTAGCTGTTTGGTTCTGTGAGGGTATTTG
pYXR44-QC-R	AGCTCCTCGCCCTTGCGCATGGCTACTGCATGCCATTCTATTC
pYXR45-QC-F	TCCTTCTCCTGATCGCTAGCGGTGGAGTGAAAGTGCTGTG
pYXR45-QC-R	AGCTCCTCGCCCTTGCGCATGGGCTTGTATCACCAGTTAATCG
pYXR46-QC-F	CTTGACTCTCCTTCTCCTGATCGCTAGCTTGATTTGCTGTCAGTATTTGTATTTAGCAG
pYXR46-QC-R	ACACCGGTGAACAGCTCCTCGCCCTTGCGCATTGCAGAAATATTCTCAATTCAGAGCTG
pYXR47-QC-F	TCCTTCTCCTGATCGCTAGCTTCTCGGTAGCATTAGCCAAGC
pYXR47-QC-R	ACCGGTGAACAGCTCCTCGCCCTTGCGCATGGTTGATTCCTATACCTTATGGATACTGG
pYXR48-QC-F	TCTCCTTCTCCTGATCGCTAGCTGCGGGGGGTATTGTGTG
pYXR48-QC-R	CACCGGTGAACAGCTCCTCGCCCTTGCGCATATTGTTCTTGCTGAGTACAGATATGATG
pYXR49-QC-F	GGACTTGACTCTCCTTCTCCTGATCGCTAGCATTGTTCTTGCTGAGTACAGATATGATG

pYXR49-QC-R	ACAGCTCCTCGCCCTTGCGCATTGCGGGGGGT ATTGTGTG	
pYXR50-QC-F	AGCGGACTTGACTCTCCTTCTCCTGATCGCTAG CGATATTTGCATATGATACAGGCCCG	
pYXR50-QC-R	CCGGTGAACAGCTCCTCGCCCTTGCGCATTACT AAAAAGTCTGTACAGCAAAGACAAAG	
pYXR51-QC-F	TCCTTCTCCTGATCGCTAGCGGGCTTGTATCAC CAGTTAATCG	
pYXR51-QC-R	AGCTCCTCGCCCTTGCGCATGGTGGAGTGAAA GTGCTGTG	
pYXR52-QC-F	TCTCCTTCTCCTGATCGCTAGCTCTGGTCCCTG CTCGATG	
pYXR52-QC-R	AGCTCCTCGCCCTTGCGCATGATGGCGGTATTT TGATGTTGTC	
pYXR53-QC-F	TCCTTCTCCTGATCGCTAGCCTTGTATTTTCTCA CCTGGCAAG	
pYXR53-QC-R	ACACCGGTGAACAGCTCCTCGCCCTTGCGCAT GATGATGTTTGAATACGAGATGCAATG	
17-stcB-F	CAACGGTCAGACGATCGGACAGGCTGGACTGA TTTCGCATTGGACTCGCAATCAGAGGG	
17-stcB-R	TATATACACAACATATTTCGTCAGACACAGAA TAACTCTCCCTGGAGCCGTTTATTGCG	
18-stcC-F	CGGTCAGACGATCGGACAGGCTGGACTGATTT CGCATTGTATAGCGAAAGTTGTGTCGC	
18-stcC-R	AACATATTTCGTCAGACACAGAATAACTCTCTT TGAAGTATTGTTTAACGGATTGGTGG	
19-stcD-F	GACGATCGGACAGGCTGGACTGATTTCGCATT TTGAAGTATTGTTTAACGGATTGGTGG	
19-stcD-R	ATACACAACATATTTCGTCAGACACAGAATAA CTCTCTGTATAGCGAAAGTTGTGTCGC	Primers for the construction of genetic circuits
20-stcE-F	TCAACGGTCAGACGATCGGACAGGCTGGACTG ATTTCGCATGGCGGCAGTACAGAACTG	
20-stcE-R	TACACAACATATTTCGTCAGACACAGAATAAC TCTCACGACTAGTGTCGAGTTGAATCG	
21-afls-F	CGATCGGACAGGCTGGACTGATTTCGCATTACT AAAAAGTCTGTACAGCAAAGACAAAG	
21-afls-R	CACAACATATTTCGTCAGACACAGAATAACTC TCGATATTTGCATATGATACAGGCCCG	
22-stcF-F	GGTCAGACGATCGGACAGGCTGGACTGATTTC GCATGCCGAATAATGTTGGATTTTGGC	
22-stcF-R	ATATACACAACATATTTCGTCAGACACAGAAT AACTCTCATTGAGTCCGGGTGAGTAGC	
23-stcG-F	AACGGTCAGACGATCGGACAGGCTGGACTGAT TTCGCATATTGAGTCCGGGTGAGTAGC	
23-stcG-R	TACACAACATATTTCGTCAGACACAGAATAAC TCTCGCCGAATAATGTTGGATTTTGGC	
24-stcH-F	TTCAACGGTCAGACGATCGGACAGGCTGGACT GATTTCGCATACTGAGGACTCAGGGGG	
24-stcH-R	ATACACAACATATTTCGTCAGACACAGAATAA CTCTCTGTTGAAGCGATCTGTTGTCTG	
25-stcI-F	CGGTCAGACGATCGGACAGGCTGGACTGATTT CGCATTGTTGAAGCGATCTGTTGTCTG	
25-stcI-R	TATATATACACAACATATTTCGTCAGACACAG AATAACTCTCACTGAGGACTCAGGGGG	
26-stcJ-F	AACGGTCAGACGATCGGACAGGCTGGACTGAT TTCGCATTCCAATAAGCGTCTTGGCTG	
26-stcJ-R	ACAACATATTTCGTCAGACACAGAATAACTCT CTCTCTTTACAGGTCGTCAAACTGTAG	
27-stcK-F	CAGACGATCGGACAGGCTGGACTGATTTCGCA TTCTCTTTACAGGTCGTCAAACTGTAG	
27-stcK-R	ATATACACAACATATTTCGTCAGACACAGAAT AACTCTCTCCAATAAGCGTCTTGGCTG	
28-stcL-F	TCAACGGTCAGACGATCGGACAGGCTGGACTG ATTTCGCATCCTGCAAGACCATGGACC	
28-stcL-R	TATACACAACATATTTCGTCAGACACAGAATA ACTCTCCTGAATGTACATAGGGCTGCC	
29-stcM-F	TCAGACGATCGGACAGGCTGGACTGATTTCGC ATGGTTTTGAAGAGTTCCAGGAGTATG	

29-stcM-R	TATATACACAACATATTTCGTCAGACACAGAA TAACTCTCAGTGACGATGGTCTGTCTG	
30-stcO-F	TCAGACGATCGGACAGGCTGGACTGATTTCGC ATCCTGATGTAGGATTAGGATAGAATG	
30-stcO-R	ACAACATATTTCGTCAGACACAGAATAACTCT CATTATTGTACTTCTACTTTAGGGTAG	
31-stcP-F	CGATCGGACAGGCTGGACTGATTTCGCATATT ATTGTACTTCTACTTTAGGGTAGAATG	
31-stcP-R	CATATTTCGTCAGACACAGAATAACTCTCCCTG ATGTAGGATTAGGATAGAATGTATAG	
32-stcQ-F	GTCAGACGATCGGACAGGCTGGACTGATTTCG CATTGTTTGGTTCTGTGAGGGTATTTG	
32-stcQ-R	TACACAACATATTTCGTCAGACACAGAATAAC TCTCGGCTACTGCATGCCATTCTATTC	
33-stcS-F	GGTCAGACGATCGGACAGGCTGGACTGATTTC GCATGGGCTTGTATCACCAGTTAATCG	
33-stcS-R	ATATACACAACATATTTCGTCAGACACAGAAT AACTCTCGGTGGAGTGAAAGTGCTGTG	
34-stcT1-F	AACGGTCAGACGATCGGACAGGCTGGACTGAT TTCGCATGGTGGAGTGAAAGTGCTGTG	
34-stcT1-R	TACACAACATATTTCGTCAGACACAGAATAAC TCTCGGGCTTGTATCACCAGTTAATCG	
35-stcT-F	AGACGATCGGACAGGCTGGACTGATTTCGCAT TGCAGAAATATTCTCAATTCAGAGCTG	
35-stcT-R	ACATATTTCGTCAGACACAGAATAACTCTCTTG ATTTGCTGTCAGTATTTGTATTTAGC	
36-stcU-F	GACGATCGGACAGGCTGGACTGATTTCGCATG GTTGATTCCTATACCTTATGGATACTG	
36-stcU-R	ATACACAACATATTTCGTCAGACACAGAATAA CTCTCTTCTCGGTAGCATTAGCCAAGC	
37-stcV-F	GACGATCGGACAGGCTGGACTGATTTCGCATA TTGTTCTTGCTGAGTACAGATATGATG	
37-stcV-R	ATATATACACAACATATTTCGTCAGACACAGA ATAACTCTCTGCGGGGGGTATTGTGTG	
38-stcW-F	TCAACGGTCAGACGATCGGACAGGCTGGACTG ATTTCGCATTGCGGGGGGTATTGTGTG	
38-stcW-R	AACATATTTCGTCAGACACAGAATAACTCTCAT TGTTCTTGCTGAGTACAGATATGATG	
6705-F	ATCGGTCTCTAATGGATCAATCATCAAGCGGA G	Primers for the construction of *nsc* gene cluster recombination plasmid
6705-R	ATCGGTCTCAAGCGTATGTTATTGGTATAGCCA TACTTTTTTCTCC	
6704-F	ATCGGTCTCTGCTCTCAAGCTGGCTTTTTCTTTT GC	
6704-R	ATCGGTCTCTAATGGGGAAGCAGCAAGAAAC	
6703-F	ATCGGTCTCTGCTCGCATTGCTGCGTACGGAAG	
6703-R	ATCGGTCTCTAATGACTTCCGTCCCTATTTTTG AG	
6702-1F	ATCGGTCTCTAATGGACAACAGTATGATGGAT TCAAC	
6702-1R	ATCGGTCTCTGAGAGAAAGTGGGCATTACCC	
6702-2F	ATCGGTCTCATCTCTAAGACCGGACAGTGC	
6702-2R	ATCGGTCTCTCGTTCCATCGGTACTTTGGC	
6702-3F	ATCGGTCTCTAACGAAACCGATTATTGGATTCC G	
6702-3R	ATCGGTCTCAAGCGCGATGATATCAATCACTCT GCCTG	
6702-1R1	ATCGGTCTCTGTGACCATCATACACAGGCAG	
6702-2F1	ATCGGTCTCATCACTGCCATGCGTCACATC	
6702-2R1	ATCGGTCTCTGGCCTCGACGGGTGAGTTG	
6702-3F1	ATCGGTCTCTGGCCTGGTGAGCTGGATTAG	
6702-3R2	ATCGGTCTCTGACACGACGATAGGTGGCG	
6702-4F1	ATCGGTCTCTTGTCTAGTCCTGTTGTAAGCAGC	

6703-1R	ATCGGTCTCTGAAACGCAGAAAGGTGTCCTAG	
6703-2F	ATCGGTCTCATTTCCAACTCCTCAACTGGGG	
6704-1R	ATCGGTCTCTGCTCTCTCATTATCGCCGC	
6704-2F	ATCGGTCTCTGAGCCCTTGTATTTGGCACC	
6704-QC-F	GACGATATTGGGTATTCCATTGTTTGTAAGGAG TCGGGAAGTTGCAAGGCCAGAAATAC	
6704-QC-R	GTATTTCTGGCCTTGCAACTTCCCGACTCCTTA CAAACAATGGAATACCCAATATCGTC	
kivr-F	AGCTTGGCATCACGCATCAGTGCCTCCTCTCAG ACAGAATGCGGTCGCAGATTGGCGTG	Primers for the construction of *beas* and kivr recombination plasmid
13-kivr-R	ACTGCAGACCATACTATTTTCGCAATAAACGG CTCCAGGATGACATCCCAAGAACACCC	
13-stcA-F	GGAGGGCCGTGAGCCAGTTTGGGTGTTCTTGG GATGTCATCCTGGAGCCGTTTATTGCG	
13-stcA-R	CCCCAAGCATAAGTTCATACTCCTGGAACTCTT CAAAACCTGGACTCGCAATCAGAGGG	
13-stcN-F	TGATGCAGATTGTCCCCCTCTGATTGCGAGTCC AGGTTTTGAAGAGTTCCAGGAGTATG	
13-stcN-R	GTATGACTGGCGTACCGCTCTTGGTGTTGAGTG ATGTCATAGTGACGATGGTCTGTCTG	
13-beas-F	TAACAGACGAGCACCATCAGACAGACCATCGT CACTATGACATCACTCAACACCAAGAG	
beas-R	TCGCCCTTTGTCATAGTAAAGTGATTCGCGTCA TGCGGCCCTCGTATACCAACGGAGGC	
PyrG-detect-F	GAGCTCGTCCGTATTGGC	
kivr-detect-R	CTGTCGTCTTTGCCCAGAAC	
14-kivr-R	AGCTAACAGACGAGCACCATCAGACAGACCAT CGTCACTATGACATCCCAAGAACACCC	
14-stcN-F	GGAGGGCCGTGAGCCAGTTTGGGTGTTCTTGG GATGTCATAGTGACGATGGTCTGTCTG	
14-stcA-R	GTATGACTGGCGTACCGCTCTTGGTGTTGAGTG ATGTCATCCTGGAGCCGTTTATTGCG	
14-beas-F	GCAGACCATACTATTTTCGCAATAAACGGCTCC AGGATGACATCACTCAACACCAAGAG	
15-stcN-R	CAACAGCATCATATCTGTACTCAGCAAGAACA ATGGTTTTGAAGAGTTCCAGGAGTATG	
15-stcW-F	TAAGTTCATACTCCTGGAACTCTTCAAAACCAT TGTTCTTGCTGAGTACAGATATGATG	
15-stcW-R	GGTATGACTGGCGTACCGCTCTTGGTGTTGAGT GATGTCATTGCGGGGGGTATTGTGTG	
15-beas-F	CCGTTTTAGACCATACAGCACACAATACCCCCC GCAATGACATCACTCAACACCAAGAG	
16-kivr-R	TATCCGTTTTAGACCATACAGCACACAATACCC CCCGCAATGACATCCCAAGAACACCC	
16-stcW-F	AGGAGGGCCGTGAGCCAGTTTGGGTGTTCTTG GGATGTCATTGCGGGGGGTATTGTGTG	
17-stcN-R	AGACCATACTATTTTCGCAATAAACGGCTCCA GGGGTTTTGAAGAGTTCCAGGAGTATG	
17-stcB-F	CCCCAAGCATAAGTTCATACTCCTGGAACTCTT CAAAACCCCTGGAGCCGTTTATTGCG	
17-stcB-R	GTATGACTGGCGTACCGCTCTTGGTGTTGAGTG ATGTCATTGGACTCGCAATCAGAGGG	
17-beas-F	CATGATGCAGATTGTCCCCCTCTGATTGCGAGT CCAATGACATCACTCAACACCAAGAG	
18-kivr-R	ATTCATGATGCAGATTGTCCCCCTCTGATTGCG AGTCCAATGACATCCCAAGAACACCC	
18-stcB-F	GGAGGGCCGTGAGCCAGTTTGGGTGTTCTTGG GATGTCATTGGACTCGCAATCAGAGGG	
1-13.1-R	TCGCCCTTTGTCATAGTAAAGTGATTCGCGTCA TGCGGCCCTACAACTCGGCAATTGCC	Primers for the construction of *eda* gene cluster recombination plasmid
1-short-2R	CCTCGGGAAATCGTGTCTTGAACACTGGCTCG ACATCCATGGTGATGTCTGCTCAAGCG	
2-15HA-R	TCATGATGCAGATTGTCCCCCTCTGATTGCGAG TCCAGCTGCAATCATTACGTCATGAC	

2-stcA-F	CTGTAAAGCGACAATATTGTCATGACGTAATG ATTGCAGCTGGACTCGCAATCAGAGGG
2-shortstcA-R	CCTCGGGAAATCGTGTCTTGAACACTGGCTCG ACATCCATCCTGGAGCCGTTTATTGCG
2-short13.1-F	GAACTGCAGACCATACTATTTTCGCAATAAAC GGCTCCAGGATGGATGTCGAGCCAGTG
2-detect-R	GCATCTGCCGATTGCAGTC
ataP-gpdA-F	TGACTAACAGCTACCCCGCTTGAGCAGACATC ACCATGCAAATCAATATTCGCAATGAG
gpdA-ataP-R	TGAGCTCACGAGCAATCTCATTGCGAATATTG ATTTGCATGGTGATGTCTGCTCAAGCG
ataP-R	GCCCTTTGTCATAGTAAAGTGATTCGCGTCATG CGGCCGGTAAACGACTCATAGGAGAG
DJ9-1-R	TGTTGAAGCGATCTGTTGTCTG
DJ9-2-F	CTCGCGATGAAAGTCCGTAGCAGACAACAGAT CGCTTCAACAATGGAGGCCAGCGTCAC
DJ9-2-R	GATAGCAGAAGAATTTAAATTAGTACACG
DJ9-3-F	TATTCGTGTACTAATTTAAATTCTTCTGCTATC ATCACGAATAGTTTCGTGTAGAATCG
DJ9-3-R	CAAGCATAAGTTCATACTCCTGGAACTCTTCAA AACCATGTGGAAGAGCGTAAGTAACG
DJ10-1-F	CTTGTGCAGGTAAACTTGTGTTG
DJ10-1-R	CTGCGAAATGATATCTGAAGAGC
DJ10-2-F	TATAAGAAGTTGAGGTGCTCTTCAGATATCATT TCGCAGCGTTCAACATGCTTGCAACC
DJ10-2-R	TTCATATCCGTTTTAGACCATACAGCACACAAT ACCCCCCGCAATGACCGCCATGTCCG
DJ10-3-F	TGCGGGGGGTATTGTGTG
DJ11-1-R	GGAACTGCAGACCATACTATTTTCGCAATAAA CGGCTCCAGGGATTGCACGCATCTGCG
DJ11-1-F	GGAGACGACAAGATAGCAGTACAC
DJ11-2-F	CCTGGAGCCGTTTATTGCG
DJ11-2-R	TGGACTCGCAATCAGAGGG
DJ11-3-F	TCATGATGCAGATTGTCCCCCTCTGATTGCGAG TCCAATGTTTGTGGCCAATCTCGATG
DJ11-3-R	GACGTGGTTATCACAAGCCAC
DJ12-1-F	GATTCAGTGTTACCTGGCACAG
DJ12-1-R	AGCATAAGTTCATACTCCTGGAACTCTTCAAAA CCATGACTACAAACGGAGTCAAACAC
DJ12-2-R	GACCTGCTAAGGCCGTGCCGTTGGTTGAAAGA CCGTTCATAGTGACGATGGTCTGTCTG
DJ12-3-F	ATGAACGGTCTTTCAACCAACG
DJ12-3-R	GACGCTAAAAGCAGTGGC
DJ13-1-F	GAAGCCTGTGGATGGAAGTC
DJ13-1-R	AATTCATGATGCAGATTGTCCCCCTCTGATTGC GAGTCCAATGTACGCAACCCAATGGC
DJ13-3-F	ACTGCAGACCATACTATTTTCGCAATAAACGG CTCCAGGATGTCTTCCTCCGAAACCAC
DJ13-3-R	CATCGAGAACGAGACGTGC
DJ14-1-F	GCGAAACATCATGCAGGC
DJ14-1-R	CATATCCGTTTTAGACCATACAGCACACAATAC CCCCCGCAATGGCGAACGAAGCACTG
DJ14-2-R	ATTGTTCTTGCTGAGTACAGATATGATG
DJ14-3-F	TGCTCAACAGCATCATATCTGTACTCAGCAAG AACAATATGACTACCTCATCCGGAACG
DJ14-3-R	CTGGCTGTTTAAGGGCGG
DJ9-nest-F	AACGACGGCCAGTGAATTCGAGCTCGGTACCC GGGGATCCGCACTGAGGACTCAGGGGG
DJ9-nest-R	AGCACCCGGTACACGGACCGAAGACGGTGTCC GGAGACATAGTGACGATGGTCTGTCTG

DJ10-nest-F	TAAGCTAACAGACGAGCACCATCAGACAGACCATCGTCACTATGTCTCCGGACACCGTC	
DJ10-nest-R	CCGGTGCGGCCGTAGTGTTCGCGAAGTGATTCATATTGTTCTTGCTGAGTACAGATATG	
DJ11-nest-F	CTCAACAGCATCATATCTGTACTCAGCAAGAACAATATGAATCACTTCGCGAACACTAC	
DJ11-nest-R	TCACAAAACAGTATACAAAAAATAAGCTTGCATGCGCGGCCCGCGCTGAGTATTCGGAG	
DJ12-nest-F	TTTACAAACGCCGAACTTGAATACACACCCTGAGCCTTCTGCTATCTCAAATAGGTCGC	
DJ12-nest-R	CGAGTGCAAAATAGTACCTCTAGCACTTGCATTTGTTCCGCTGGAAACTCGACACATAG	
Dj13-nest-F	CGGCAGAAATCAAAAACTATGTGTCGAGTTTCCAGCGGAACAAATGCAAGTGCTAGAGG	
Dj13-nest-R	GTCTCCTCGGTGCCTGGAACCCAACAGACGCTAGCTCATCCGACATGGAGCAGCTGCTG	
Dj14-nest-F	GCCGTCCAGCACCATCATCTCCAGCAGCTGCTCCATGTCGGATGAGCTAGCGTCTGTTG	
DJ14-nest-R	CGGGATACTAAGAGGCTTCATGTGGAAGAGCGCGGCCGCTGGGTGTGATGTGAAGATTG	
210-detect-R	GGAGTCATTGGGTGGAACTG	
209-detect-R	CCATTCTTCGTCCGAACCAG	
1-RT-F	GCATGAACCACTCTATCATGTCG	Primers for RT-PCR of *eda* cluster
1-RT-R	ATGGCGAACGAAGCACTG	
2-RT-F	GGACAAACCGACATGGCT	
2-RT-R	CACTCGTCGCTGAGCATC	
3-RT-F	CTGGGCAGGTCGTAAACG	
3-RT-R	GGGTGGAGTCATTGGGTG	
4-RT-F	GCGCGAACAATTCATCCG	
4-RT-R	GTAACGCTGGCCAGGAATC	
5-RT-F	CCGCTTCTTCACTTCCCG	
5-RT-R	CTGCTGCAGACATGGCTC	
7-RT-F	GGGTATCTCCGAGGCTCC	
7-RT-R	GATTGACCTGGGAACGGC	
8-RT-F	CATCATTGGAGGTGGCCC	
8-RT-R	CCCAACGCAACCTGTTCC	
9-RT-F	CTCTCTTCCCGCTCGTAC	
9-RT-R	CGTTGAGGAGACCTTGCC	
10-RT-F	GGCGCTTGATGCTCGTAC	
10-RT-R	CTTCTACGTCTCGCAGGC	
11-RT-F	CAATCCCAGGCATCACCG	
11-RT-R	GTGGCTGGTGGTATGGTG	
12-RT-F	CCTCATCCGGAACGTTGG	
12-RT-R	GGCGTAGCACCGTCAATC	
EDgtmA-RT-F	GCCCTGTCCTCTTCCTCC	
EDgtmA-RT-R	GGGCAGCATGAAGACTGC	
actin-RT-F	CCTCACACAACCACACCG	
actin-RT-R	CATCCTCATCGTCGCGTC	
DJ-1-5F-F	CTTGATTGATCGGAACGCCG	Primers for the construction of *mpa* gene cluster recombination plasmid
DJ-1-5F-R	ATGACCAACGCAGTGGAG	
DJ-1-stcE-F	AGCAGGTCCCTAGGACCAGAGTCCTCCACTGCGTTGGTCATGGCGGCAGTACAGAACTG	
DJ-1-stcE-R	ACGACTAGTGTCGAGTTGAATCG	

DJ-1-stcM-F	GGCGAACCCTTACCCGATTCAACTCGACACTA GTCGTAGTGACGATGGTCTGTCTGATG
DJ-1-stcM-R	CTGGAGGCAAAGACAAAAACATGCTGCTATTC ATGGTTTTGAAGAGTTCCAGGAGTATG
DJ-1-3F-F	ATGAATAGCAGCATGTTTTTGTCTTTG
DJ-1-3F-R	GTTAGATTCAGTCTGTGTATGTTTTCAC
DJ-2-5F-F	GACGGTGAAAACCTCTGACAC
DJ-2-5F-R	AGTGACGATGGTCTGTCTGATG
DJ-2-stcB-F	AAGCTAACAGACGAGCACCATCAGACAGACCA TCGTCACTCCTGGAGCCGTTTATTGCG
DJ-2-stcB-R	AGATGGTCGGTATAACACGAGGATCAGAGACT GAAACCATTGGACTCGCAATCAGAGGG
DJ-2-3F-F	ATGGTTTCAGTCTCTGATCCTCG
DJ-2-3F-R	GAAAGTCTCTGTGCGTGTCG
DJ-3-5F-F	GCTCTTCCACATGAAGCCTC
DJ-3-5F-R	TCTTCACAGCCACGAGAAGAAGTTATACTCTTC TTAAGCTGCAATCATTACGTCATGAC
DJ-3-mpaF-F	TTAAGAAGAGTATAACTTCTTCTCGTGGC
DJ-3-mpaF-R	ATGGTCGAAATTCTGGACTACAC
DJ-3-3F-F	AGGACCTCCAAGGCCTTGGTGTAGTCCAGAAT TTCGACCATTGCGGGGGGTATTGTGTG
DJ-3-3F-R	GAGTCAGTGAGCGAGGAAGC
DJ-4-5F-F	CGCCTCTCATGTACGATCTTTG
DJ-4-5F-R	ATGAGTGCCGCATCCC
DJ-4-stcI-F	GCTCCGGGATAATGGAGGCTGGGGATGCGGCA CTCATTGTTGAAGCGATCTGTTGTCTG
DJ-4-stcI-R	AGTTCAACTTCAATTTCGAAGTGGCGAAGCCA ATGTAACCAGACTGAGGACTCAGGGGG
DJ-4-mpaH-F	CTGGTTACATTGGCTTCGC
DJ-4-mpaH-R	ATGTCAACTGAGAAATTTACCATCACTG
DJ-4-3F-F	GGACCAGGTGCTCAGTGATGGTAAATTTCTCA GTTGACATCCTGGAGCCGTTTATTGCG
DJ5-stcI-F	GGTCCCTAGGACCAGAGTCCTCCACTGCGTTG GTCATTGTTGAAGCGATCTGTTGTCTG
DJ-5-stcI-R	GCGCAGGCGAACCCTTACCCGATTCAACTCGA CACTAGTCGTACTGAGGACTCAGGGGG
DJ-5-stcE-R	GAAAGTGCTGGAGGCAAAGACAAAAACATGCT GCTATTCATGGCGGCAGTACAGAACTG
DJ-6-5F-R	ATTGTTCTTGCTGAGTACAGATATGATG
DJ-6-stcA-F	ACTGCTCAACAGCATCATATCTGTACTCAGCAA GAACAATTGGACTCGCAATCAGAGGG
DJ-6-stcA-R	AGATGGTCGGTATAACACGAGGATCAGAGACT GAAACCATCCTGGAGCCGTTTATTGCG
DJ-7-stcM-F	CCAAGGCCTTGGTGTAGTCCAGAATTTCGACC ATGGTTTTGAAGAGTTCCAGGAGTATG
DJ-8-stcB-F	CAAGCTCCGGGATAATGGAGGCTGGGGATGCG GCACTCATTGGACTCGCAATCAGAGGG
DJ-8-stcB-R	TTCAACTTCAATTTCGAAGTGGCGAAGCCAAT GTAACCAGCCTGGAGCCGTTTATTGCG
DJ-8-3F-F	GGTGCTCAGTGATGGTAAATTTCTCAGTTGACA TGGTTTTGAAGAGTTCCAGGAGTATG
DJ-1-nest-F	AGAATCCTATAAATTGGGAAAACTAGAGACCC CGGTCGCCACAATCCCAATCCTCGGAC
DJ-1-nest-R	GAAGGATATGAGTTCTTGAGAAGACATGTCCA GCTTTAGGTAGCCCGCGGTAATGATTC
mpaC-nest-F	GGAGTTTCGGCCTTGAATCATTACCGCGGGCTA CCTAAAGCTGGACATGTCTTCTCAAG
mpaC-nest-R	TCCGTTTTAGACCATACAGCACACAATACCCCC CGCAATGAATTTCCACAAAGGGCAAC
DJ-6-nest-F	GGACCCGAAGATCTTCCTTCGGTTGCCCTTTGT GGAAATTCATTGCGGGGGGTATTGTG

Dj-2-nest-R	AATCGGGATACTAAGAGGCTTCATGTGGAAGA<br>GCGCGGCCGCCAGTAGAGCCCATATCG
DJ-3-nest-F	GGCATCACGCATCAGTGCCTCCTCTCAGACAG<br>AATGCCATTGTCTCGCGGTATAGCATC
DJ-7-nest-R	CAGATCAAGCGTCATCCTTCGTTCCACACCGAC<br>GATCAGTGACGATGGTCTGTCTGATG
DJ-4-nest-F	AACTGCTCAACAGCATCATATCTGTACTCAGCA<br>AGAACAATGATCGTCGGTGTGGAACG
DJ-8-nest-R	CCCTTTGTCATAGTAAAGTGATTCGCGTCATGC<br>GGCCAGTGACGATGGTCTGTCTGATG
DJ-4-nest-R	TCGCCCTTTGTCATAGTAAAGTGATTCGCGTCA<br>TGCGGCCTGGACTCGCAATCAGAGGG
DJ-8-nest-F	TAAGCTAACAGACGAGCACCATCAGACAGACC<br>ATCGTCACTGATCGTCGGTGTGGAACG
DJ-3-nest-R	AAGCGTCATCCTTCGTTCCACACCGACGATCAT<br>TGTTCTTGCTGAGTACAGATATGATG
mpaC-nest-R-LSJ5	CAAGCATAAGTTCATACTCCTGGAACTCTTCAA<br>AACCATGAATTTCCACAAAGGGCAAC
DJ-2-nest-F	GATCTTCCTTCGGTTGCCCTTTGTGGAAATTCA<br>TGGTTTTGAAGAGTTCCAGGAGTATG

Table S6. Enzyme function predicted through deep bioinformatic annotation of *eda* cluster

Protein	Identity/coverage (%) to genes of				putative function
Epicoccum dendrobii	*Aspergillus fumigatus* AF293	*Aspergillus terreus* NIH2624	*Aspergillus oryzae* RIB40	*Trichoderma hypoxylon*	
EdaM	GliM (41/99)	AtaM (34/78)	AclM (43/100) AclU (34/98)	TdaU (45/98)	O-methyltransferase
EdaG	GliG (38/97)	AtaG (55/83)	AclG (64/95)	TdaL (55/99)	glutathione S-transferase
EdaS	GtmA (25/100)	AtaS (48/100)	-	-	SAM-methyltransferase
EdaI	GliI (33/99)	-	-	TdaT (30/99)	carbon−sulfur lyase
EdaT	GliT (59/99)	-	AclT (32/99)	-	sulfhydryl oxidase
EdaF	GliF (48/86)	-	-	-	cytochrome P450
EdaA	GliA (39/96)	-	-	-	MFS transporter
EdaP	-	AtaP (37/99)	AclP (41/99)	-	NRPS
EdaC	-	AtaC (36/99)	-	-	cytochrome P450
EdaB	-	-	-	-	cytochrome P450
EdaJ	-	-	-	-	dipeptidase
EdaZ	-	-	-	-	Zn(2)-C6 transcription factor
EdaN	-	-	-	-	SAM-methyltransferase

Table S7. NMR data of boydines A (**1**) (500 MHz for ^{1}H-NMR and 125 MHz for ^{13}C-NMR)

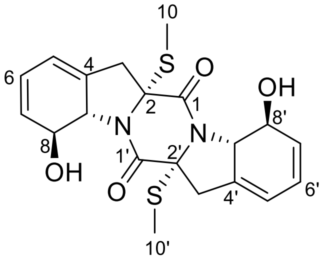

Position	δ_C, type	δ_H, muti., J in Hz
1	169.29, C	/
2	75.85, C	/
3	38.90, CH_2	3.05, (d, J = 15.8, 1H)<br>3.11, (d, J = 15.8, 1H)
4	134.25, C	/
5	120.72, CH	5.99, (m, 1H)
6	124.74, CH	5.94, (m, 1H)
7	130.77, CH	5.68 (d, J = 9.8, 1H)
8	74.56, CH	4.89 (d, J = 13.4, 1H)
9	69.38, CH	4.81 (d, J = 13.4, 1H)
10	14.75, CH_3	2.25 (s, 3H)
1'	169.29, C	/
2'	75.85, C	/
3'	38.90, CH_2	3.05, (d, J = 15.8, 1H)<br>3.11, (d, J = 15.8, 1H)
4'	134.25, C	/
5'	120.72, CH	5.99, (m, 1H)
6'	124.74, CH	5.94, (m, 1H)
7'	130.77, CH	5.68 (d, J = 9.8, 1H)
8'	74.56, CH	4.89 (d, J = 13.4, 1H)
9'	69.38, CH	4.81 (d, J = 13.4, 1H)
10'	14.75, CH_3	2.25 (s, 3H)

NMR data for boydines A (**1**) was recorded in CD_3OD.

Table S8. NMR data of dendrobine A (**2**) (500 MHz for ^{1}H-NMR and 125 MHz for ^{13}C-NMR)

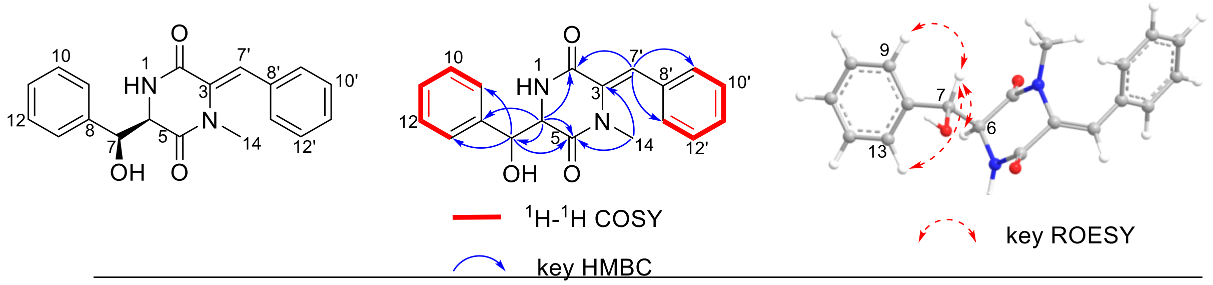

Position	δ_C, type	δ_H, muti., *J* in Hz	HMBC correlation	^{1}H-^{1}H COSY	^{1}H-^{1}H ROESY
1	/	/	/	/	/
2	165.75, C	/	/	/	/
3	133.61, C	/	/	/	/
4	/	/	/	/	/
5	168.97, C	/	/	/	/
6	64.07, CH	4.19, (d, *J* = 2.8, 1H)	C-2, 5, 7, 8	H-7	H-7
7	76.26, CH	5.16 (d, *J* = 2.8, 1H)	C-5, 9, 13	H-6	H-6, 9, 13
8	141.72, C	/	/	/	/
9	127.55, CH	7.37 (overlap, 1H)	/	H-10	/
10	129.36, CH	7.37 (overlap, 1H)	/	H-9, 11	/
11	129.00, CH	7.37 (overlap, 1H)	/	H-10, 12	/
12	129.36, CH	7.37 (overlap, 1H)	/	H-11, 13	/
13	127.55, CH	7.37 (overlap, 1H)	/	H-12	/
14	36.10, CH_3	/	C-3, 5	/	/
7'	120.47, CH	7.10 (s, 1H)	C-2, 9', 13'	/	/
8'	135.75, C	/	/	/	/
9'	130.52, CH	7.37 (overlap, 1H)	/	H-10'	/
10'	129.53, CH	7.37 (overlap, 1H)	/	H-9', 11'	/
11'	129.36, CH	7.37 (overlap, 1H)	/	H-10', 12'	/
12'	129.53, CH	7.37 (overlap, 1H)	/	H-11', 13'	/
13'	130.52, CH	7.37 (overlap, 1H)	/	H-12'	/

NMR data for dendrobine A (**2**) was recorded in CD_3OD.

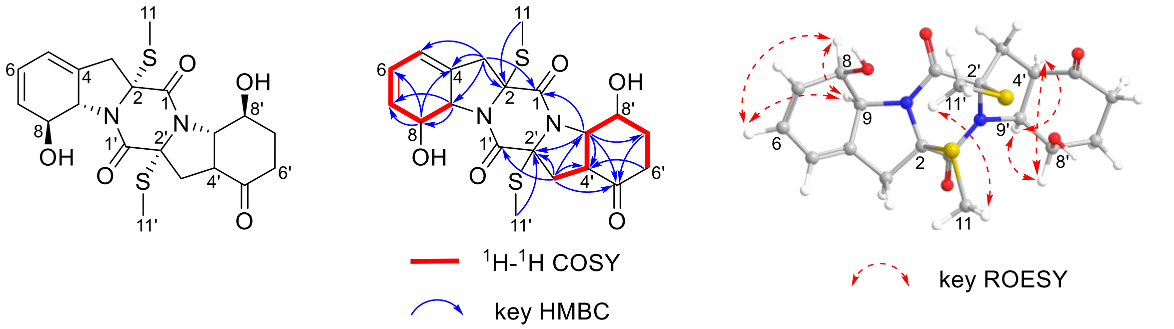

Table S9. NMR data of dendrobine B (**3**) (500 MHz for ^{1}H-NMR and 125 MHz for ^{13}C-NMR)

Position	δ_C, type	δ_H, muti., *J* in Hz	HMBC correlation	^{1}H-^{1}H COSY	^{1}H-^{1}H ROESY
1	167.68, C	/	/	/	/
2	74.92, C	/	/	/	/
3	38.90, CH_2	3.04 (s, 2H)	C-1, 2, 4, 5, 9	/	/
4	134.43, C	/	/	/	/
5	120.62, CH	5.97 (m, 1H)	/	H-6	/
6	130.60, CH	5.66, (d, *J* = 9.7, 1H)	/	H-5, 7	H-8
7	124.71, CH	5.92 (m, 1H)	/	H-6, 8	/
8	75.82, CH	4.76 (dt, *J* = 13.4, 2.5, 1H)	C-4, 6, 7	H-7, 9	H-6, 9
9	69.26, CH	4.83 (m, 1H)	C-7, 8	H-8	H-6, 8
10	/	/	/	/	/
11	14.58, CH_3	2.20 (s, 3H)	C-2	/	H-11'
1'	169.50, CH	/	/	/	/
2'	72.64, C	/	/	/	/
3'	35.24, CH_2	2.34 (m, 1H)<br>3.10 (overlap, 1H)	C-1', 2', 4', 5', 9'	H-4'	/
4'	45.80, CH	3.10 (overlap, 1H)	/	C-3', 9'	H-8', 9'
5'	209.69, C	/	/	/	/
6'	35.37, CH_2	2.41 (dt, *J* = 18.1, 5.7, 1H)<br>2.70 (m, 1H)	C-4'	H-7'	/
7'	27.20, CH_2	2.03 (m, 1H)<br>2.30 (m, 1H)	C-5'	H-6', 8'	/
8'	68.31, CH	4.40 (ddd, *J* = 7.0, 4.3, 2.6, 1H)	/	H-7', 9'	H-4', 9'
9'	66.88, CH	4.53 (dd, *J* = 8.8, 4.3, 1H)	C-4', 5', 7'	H-4', 8'	H-4', 8'
10'	/	/	/	/	/
11'	14.97, CH_3	2.08 (s, 3H)	C-2'	/	H-11

NMR data for dendrobine B (**3**) was recorded in CD_3OD.

References:

92 J. Yaegashi, B. R. Oakley, C. C. Wang, J. Ind. Microbiol. Biotechnol. 2014, 41, 433.

93 L. Liu, S. Liu, L. Jiang, X. Chen, L. Guo, Y. Che, Org. Lett. 2008, 10, 1397.
